# Pathogenesis of Systemic Sclerosis

**DOI:** 10.3389/fimmu.2015.00272

**Published:** 2015-06-08

**Authors:** Debendra Pattanaik, Monica Brown, Bradley C. Postlethwaite, Arnold E. Postlethwaite

**Affiliations:** ^1^Department of Medicine, Division of Connective Tissue Diseases, The University of Tennessee Health Science Center, Memphis, TN, USA; ^2^Department of Veterans Affairs Medical Center, Memphis, TN, USA; ^3^Section of Pediatric Rheumatology, Department of Pediatrics, The University of Tennessee Health Science Center, Memphis, TN, USA

**Keywords:** systemic sclerosis, scleroderma, innate immunity, adaptive immunity, vasculopathy, fibrosis, animal models

## Abstract

Systemic scleroderma (SSc) is one of the most complex systemic autoimmune diseases. It targets the vasculature, connective tissue-producing cells (namely fibroblasts/myofibroblasts), and components of the innate and adaptive immune systems. Clinical and pathologic manifestations of SSc are the result of: (1) innate/adaptive immune system abnormalities leading to production of autoantibodies and cell-mediated autoimmunity, (2) microvascular endothelial cell/small vessel fibroproliferative vasculopathy, and (3) fibroblast dysfunction generating excessive accumulation of collagen and other matrix components in skin and internal organs. All three of these processes interact and affect each other. The disease is heterogeneous in its clinical presentation that likely reflects different genetic or triggering factor (i.e., infection or environmental toxin) influences on the immune system, vasculature, and connective tissue cells. The roles played by other ubiquitous molecular entities (such as lysophospholipids, endocannabinoids, and their diverse receptors and vitamin D) in influencing the immune system, vasculature, and connective tissue cells are just beginning to be realized and studied and may provide insights into new therapeutic approaches to treat SSc.

## Introduction

Systemic sclerosis (SSc, scleroderma) is a complex connective tissue disease of unknown etiology with multiorgan involvement and heterogeneous clinical manifestations. The clinical and pathologic manifestations of the disease are the result of three distinct processes: (1) innate and adaptive immune system abnormalities leading to production of autoantibodies and cell-mediated autoimmunity, (2) microvascular endothelial cells (MVEC) and fibroproliferative vasculopathy of small vessels, and (3) fibroblast dysfunction leading to excessive collagen (CI) and other matrix components accumulation in skin, blood vessels, and internal organs (1, 2).

The incidence of SSc is about 20 cases per million populations per year and the prevalence is more than 250 patients per million populations in USA (3). Major organ involvement leads to decreased survival in SSc. Pulmonary fibrosis [interstitial lung disease (ILD)] and pulmonary arterial hypertension (PAH) cause more than half of all SSc-related deaths (3). However, patients with SSc live longer and cardiac deaths are increasing.

Progressive thickening and fibrosis of skin secondary to excessive CI accumulation is the most evident and universal finding and can be associated with varying degrees of fibrosis of internal organs. Vascular dysfunction and abnormalities are often seen, and can precede organ involvement by several years (4).

Disease manifestations vary from limited skin involvement with minimal systemic involvement [limited cutaneous (lc) SSc] to widespread skin involvement accompanied by internal organ involvement [diffuse cutaneous (dc) SSc]. These two forms differ mainly in regards to extent of skin involvement, autoantibody association, and the pattern of organ involvement (Table 1) (5). Given the heterogeneity of clinical symptoms and signs, American College of Rheumatology (ACR)/EULAR recently developed new classification criteria (6). The new classification criteria would improve sensitivity, which would lead to earlier diagnosis, and it also incorporates the autoantibodies that are commonly used for diagnostic purposes.

**Table 1:** **Important differences between limited cutaneous systemic sclerosis (lcSSc) and diffuse cutaneous systemic sclerosis (dcSSc)**.

Features	lcSSc	dcSSc
Skin	Skin thickening occurs late, limited to the distal part of upper and lower extremities, face, neck, and upper chest. Telangiectasias and calcinosis are common. Tendon friction rub not seen	Skin thickening occurs early, moves up to proximal part of extremities and trunk. Telangiectasias and calcinosis may occur late in disease. Tendon friction rub present
GI	Esophageal dysmotility is more common than small and large intestine involvement	Esophageal dysmotility is frequently seen. Small and large intestinal involvement is more common
Pulmonary	Pulmonary fibrosis is less frequent and less severe. Frequent and severe pulmonary hypertension is more common	Pulmonary fibrosis is more common and severe. Pulmonary hypertension is less frequent
Kidney	Renal crisis uncommon	Renal crisis is more frequent
Autoantibody association	Anticentromere antibodies (ACA) are predominant	Anti-DNA topoisomerase I antibody (ATA) (Anti-Scl-70) antibody is predominant
		Anti-RNA polymerase antibody is more common

It is widely believed that SSc develops in an individual with a “permissive” genetic makeup. Genetic associations of SSc are summarized below. A triggering event such as an infection or environmental toxin has been implicated as starting the processes that lead eventually to SSc in individuals with a permissive genetic background. The realization that an “interferon (IFN) signature” exists in most patients with SSc implies activation of the innate immune system and lends validity to the long-held suspicion that infections (such as with cytomegalovirus, Epstein-Barr virus, and more recently *Toxoplasma gondii*) could be SSc triggers in receiving more attention and a re-examination (7, 8). There is mounting evidence that the microbiota may play a role in development of autoimmunity, an area that is unexplored in SSc (9). Analysis of skin transcriptome has identified high levels of *Rhodotorula* sequences in dcSSc patients (10).

No animal model develops SSc that faithfully replicates human SSc, and this has impeded our understanding of the disease. There are many unresolved questions related to the etiopathogenesis of SSc. For example, it is unclear whether the innate/adaptive immune system abnormalities, vasculopathy, and fibroblast dysfunctions are separate, unrelated processes or are mechanistically linked, which of the three processes is of utmost importance and how interaction among the three processes leads to the development of the disease. These three processes will be discussed.

**Figure 1:**
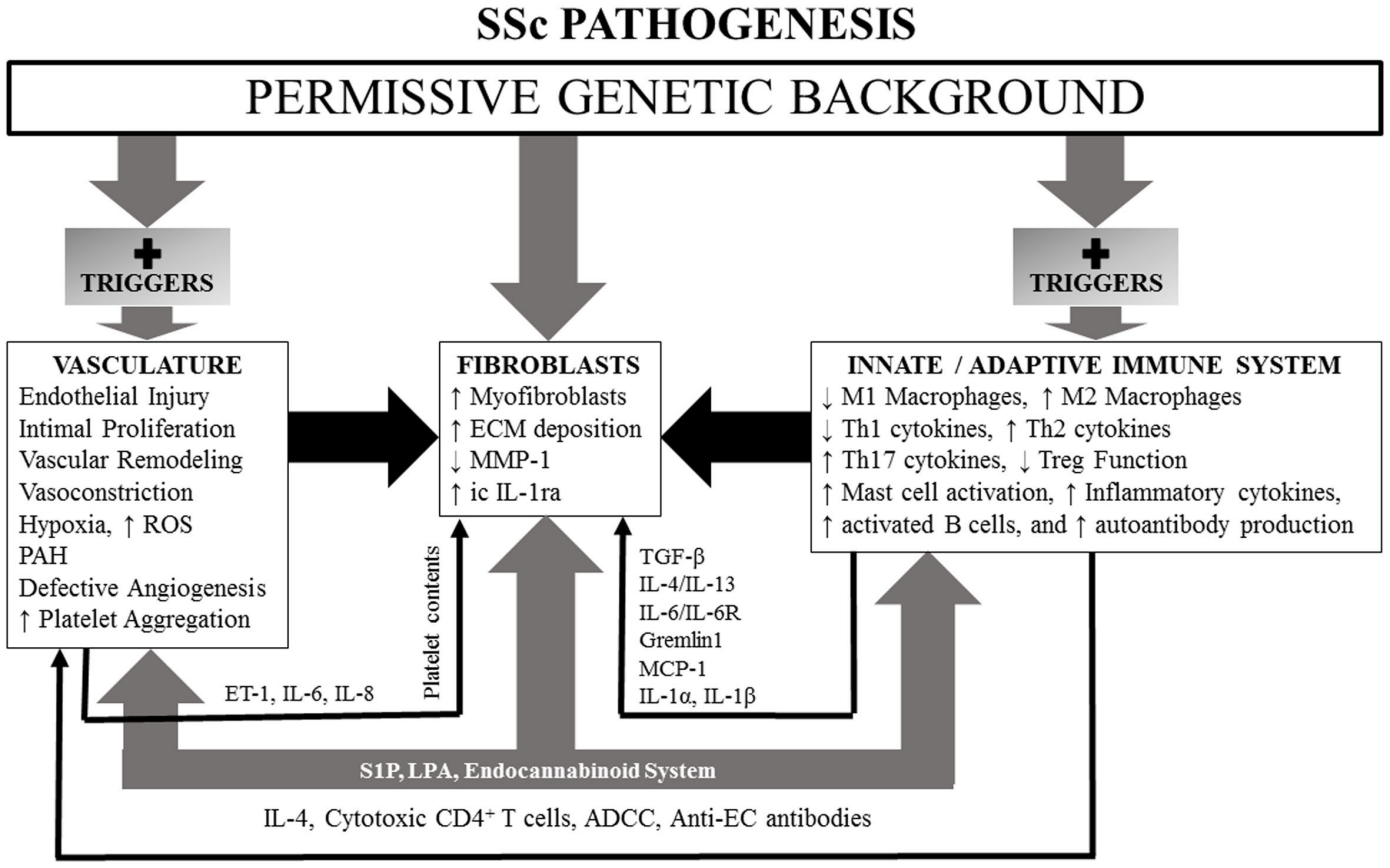
**A simplified schematic of SSc pathogenesis, illustrating influences of a permissive genetic background and lysophospholipids and endocannabinoid system participation which have the capacity, if dysregulated, to effect changes in vasculature, fibroblasts, and innate and adaptive immune systems**. See text for details.

We first review evidence for genetic abnormalities in SSc since they can influence responses of the innate and adaptive immune systems, vascular function, connective tissue metabolism, and fibroblast function. Since the innate and adaptive immune systems are the first to respond to environmental triggers, be they infections or toxins in nature, and through generations of cytokines, chemokines, and growth factors that can affect function of vascular and connective tissue cells, we discuss them next. The vascular abnormalities and fibrosis in SSc are then discussed. The endocannabinoid system (ECS) (which influences functions of the immune system, vasculature, and fibroblasts) may be dysregulated in SSc as suggested by recent studies of SSc dermal fibroblasts. We have included a discussion of this important system with special emphasis on potential ECS targets that might offer new therapeutic approaches for management of SSc. Lysophospholipids [lysophosphatidic acid (LPA) and sphingosine 1-phosphate (S1P)] and their different receptors (which regulate immunity, vascular physiology, and fibrosis) are dysregulated in SSc and likely contribute to the pathogenesis of the disease. Vitamin D (VitD) status also impacts function of most cell types and likely influences pathogenesis and clinical features of SSc. An overall scheme of SSc pathogenesis is illustrated in Figure 1.

## Genetics of SSC

Genetic influences have long been suspected to impact SSc. In families with a history of SSc, the incidence of disease can range from 1.5 to 1.7% (11). Having a family history of SSc increases the risk of developing disease 15–19-fold in siblings and 13–15-fold in first-degree relatives (11–13). Over the last decade, candidate gene study (CGS) approach and genome-wide association studies (GWAS) have been used to identify genetic associations that confer susceptibility to SSc. CGS and GWAS have allowed for the identification of genetic variations [single nucleotide polymorphisms (SNPs)] that are likely to be involved with the pathogenesis of scleroderma. CGS analyses SNPs to determine if the gene has association with a disease or a disease trait. The SNPs being studied have been selected based on their known association with other autoimmune diseases or on their possible functional relevance in the disease pathogenesis. GWAS arrays on the other hand, use tagSNPs to scan the entire genome to identify millions of SNPs. It takes into consideration the haplotype structure of the population being studied. Unlike CGS, GWAS identifies SNPs in a non-hypotheses-driven manner and allows for the identification of newly identifiable genes that were not previously identified in the disease. As regards to SSc, GWAS has confirmed major histocompatibility complex (MHC) II region as being most significant in this disease. Both CGS and GWAS have identified multiple genes that have been found to have firm associations in the pathogenesis of SSc.

Performing a GWAS can be very costly. Recently, the immunochip consortium was developed and implemented the immunochip analysis assay. The immunochip array provides high-density mapping of autoimmune diseases-associated loci using a custom SNP genotyping array (14). It was designed to increase efficiency of mapping autoimmunity risk loci and to reduce the cost of mapping (15). The immunochip uses variants from across 186 known autoimmunity risk loci and places them on an Illumina Infinium array platform. The platform contains 196,524 different variants of autoimmunity risk loci that may have functional significant effects in diseases like SSc. It also identifies variants with lower penetrance using a cost efficient strategy (14). Many of these genes have been firmly established in the pathogenesis of SSc. In this review, we will focus on genetic associations in MHC – human leukocyte antigen (HLA, Table 2), non-HLA genetic SNP (Table 3), and microRNAs (miRNAs) (Table 4). We will focus on the most relevant associations first and then discuss others that may have modest effects on SSc.

**Table 2:** **HLA genes associated with SSc**.

HLA-associated genes	Population (*n* = SSc)	Disease phenotype and/or clinical features	Autoantibody association	Reference
**HLA CLASS I GENES ASSOCIATED WITH SSc**				
A*30	Brazil (141)	Pulmonary fibrosis	ATA	(16, 17), (16, 18–22), (17), (17), (16), (18), (17), (23)
B*13	European ancestry (95)	PAH		
B*35	Brazil	PAH		
	Native Indian	dcSSc		
	Hispanic			
B*62	European ancestry (95)	dcSSc and high skin scores, pulmonary fibrosis		
B*65		PAH		
C*04	Brazil	PAH		
Cw4	Native Indian	Pulmonary fibrosis	dcSSc pulmonary fibrosis ATA	
Cw*0602	European ancestry (95)	Pulmonary fibrosis	ARA	
G	European ancestry	Lower vascular cutaneous ulcers telangiectasias arthropathy	dcSSc	
	African			
	Brazilian			
**HLA CLASS-II GENES ASSOCIATED WITH SSc**				
DPA1/B1	European ancestry (5,471)	Pulmonary fibrosis	ATA	(24–26)
DPB1*1301	Korean Chinese (338)		ACA, ATA, and dcSSc	
*0901			ATA	
DPB1/B2 SNPs				
rs3128930				
rs7763822/rs7764491	European ancestry (1,107)		ACA	
rs3117230/rs3128965				
rs3117230	Caucasian			
rs7763822	African American		ACA	
rs7764491	Hispanic		ATA	
rs3117230				
rs312965				
rs3128965A				
DPA1/B1				
DQA1	African American	Pulmonary fibrosis	ATA/ACA	(16, 18, 19, 26–30)
*0501	Native American		dcSSc	
DQB1			ACA	
*03:01	European ancestry		Anti-U1RNP/	
*03:03	Korean/Chinese		ATA	
*04:00	Hispanic			
*05:01	Italian			
*06:11	Brazilian		ATA	
*26:00	Native American			
^71^TRAELDT^77^	French (282)		dcSSc ATA	
DRB1	European ancestry			(17–19, 29–34)
	Hispanic			
	Italian			
*01			ACA	
*0404			ACA	
*05			ACA	
*07				
*0804	African American (278)		AFA/ARA	
*11		Increased skin score, pulmonary fibrosis	ATA	
*1104			ACA	
			ATA	
*1502	Thai (50)	No association PF, DU, sclerodactyly, myositis, SICCA	ATA	
*1602	Native American (32)	dcSSc PF	ATA	
*0407	European ancestry	Renal crisis dcSSc		
	African American			
	Hispanic (1,517)			
*1304	European ancestry			
	African American			
	Hispanic (1,517)			
DRB5*01:02	Thai (50)		ATA	
^67^FLEDR^71^	French (282)	dcSSc	ATA	
NOTCH4	European ancestry (5,471)		ACA	(24)
			ATA	
**GENES WITH NEGATIVE ASSOCIATION FOR SSc**				
C*03	Brazilian	PAH	ATAq	(16, 27, 30, 33)
	Spanish			
DQA1	European ancestry (1,300)			
*0201	Hispanic			
*0501	Italian Spanish (940)	dcSSc		
DQB1	European ancestry			
*0202	Hispanic			
*04	Brazilian	dcSSc		
DRB1	Brazilian			
*01	Spanish			
*04	Thai			
*0701				
*1501				

## HLA Association with SSc

The HLA-1 complexes HLA-A, -B, -C, and -G and HLA class-II complexes HLA-DP, -DQ, and -DR have all been identified in SSc susceptibility (11, 17, 108, 109) (Table 2). HLA class-II is the most significant region associated with SSc (14). HLA-DRB1*01, HLA-DRB1*11, HLA-A*30, and HLA-A*32 have SSc susceptibility, while HLA-DRB1*07, HLA-B*57, and HLA-Cw*14 are protective against SSc (17). HLA alleles DRB1*0802 and DQA1*0501 are associated with increased mortality (110). Clinical features of disease, disease phenotype, and SSc-specific autoantibodies have been distinguished based on HLA subtypes (Table 1). In a GWAS study that included 5471 SSc patients of European ancestry, *HLA-DQB1* locus was associated with anticentromere antibodies (ACA), *HLA-DPA1/B1* loci with anti-DNA topoisomerase I antibody (ATA), and neurogenic locus notch homolog 4 (NOTCH4) with ACA and ATA (24). In another study that included SSc patients of African American (AA) and Hispanic descent, DRB1*1104, DQA1*0501, DQB1*0301, and *DQB1* had strong positive association in SSc patients of Hispanic and of European ancestry (24, 31). DRB1*0404, DRB1*11, and DQB1*03 alleles are associated with anti-U3 ribonucleoprotein (ARA) in this subpopulation (24). In this same subpopulation, DRB1*0701, DQA1*0201, DQB1*0202, and DRB1*1501 had a negative or protective association against SSc (27). These studies have also identified DRB1*11 with association with ATA and DRB1*01, DRB1*04, and DRB1*0501 have association with ACA (31). *HLA-DPB1* and *HLA-DPB2* SNPs rs7763822/rs7764491 and rs3117230/rs3128965 have strong association with ATA or ACA positivity (25). In AA patients with SSc, DRB1*0804, DQA1*0501, and DQB1*0301 are associated with SSc (28), and have a higher frequency of ARA or anti-fibrillarin antibody (AFA) positivity (111).

HLA-DRB1*1101, *1104, *1501, and *0802 (commonly associated with the dcSSc subset) show the amino acid sequence ^67^FLEDR^71^ in their β chain, whereas *HLA-D Q* β*1* alleles *D301, *0302, *0401, *0402, *0601, and *0602 (commonly associated with SSc) show a ^71^TRAELDT^77^ motif on their β chain (29). In a study in French SSc patients with European ancestry, both FLEDR and, to a lesser degree, TRAELDT were associated with dcSSc (29). Addition of a tyrosine at position 30 strengthened the TRAELDT association with dcSSc (29). Further analysis showed that the FLEDR motif had the highest association with SSc patients who were ATA positive, while TRAELDT had lesser association in this subset (29). The TRAELDT association with ATA positivity and dcSSc were not dependent entirely on FLEDR (29). The authors concluded that double dose of the shared epitope, as well as compound heterozygosity, may confer a higher risk for development of SSc.

*HLA-DPB1* and *-DPB2* are reported to have strong susceptibility with SSc in the Korean and Chinese populations (25). Subtypes DPB1*1301 and DPB1*0901 were most common in Korean patients with SSc, while DPB1*03:01, DPB1*13:01, DQB1*03:03, DQB1*05:01, and DQB1*06:11 were significantly increased in the Chinese SSc patient population (26). Those who carried the DPB1*03:01 had a higher chance of developing pulmonary fibrosis verses those who carried DPB1*04, and those SSc patients were more likely to be ACA positive (112). DQB1*03:03 and DQB1*05:01 were strongly associated with ACA, while DQB1*06:11 was associated with ATA positivity and a marginal association with pulmonary fibrosis. DQB1*03:01 had an increase frequency of anti-U1RNP positivity in Chinese patients with SSc (26).

The role of HLA II in Italian and Spanish SSc patients has also been examined. HLA-DRB1*1104, DQA1*0501, and DQB1*0301 haplotypes are overexpressed in this patient population (30). Carrying the HLA-DQB1*03 and HLA-DRB1*11 alleles are risk factors for developing SSc in this subset of patients. Having the HLA-DRB1*0701 allele was protective (30). HLA-DRB1*1104 allele has association with ATA, while HLA-DQB1*0501 in ATA patients is protective (30). ACA-positive patients expressed HLA-DRB1*01 and -DQB1*05. Patients who had pulmonary fibrosis were found to have an association with DRB1*11 (32).

HLA-A*30 and -DQB1*04 alleles were found to relate to SSc susceptibility in a subset of Brazilian patients (16). In patients who had PAH, HLA-B*35, and C*04 were associated as risk genes for this complication, while C*03 was protective (16). HLA-DRB1*15:02 and DRB5*01:02 are associated with ATA positivity in SSc Thai patients. There were no associations seen between these genes and other clinical manifestations of disease including pulmonary fibrosis, digital pits, sclerodactyly, myositis, or SICCA symptoms. DRB1*04 was protective in this patient population (33).

In a population of French SSc patients of European ancestry, amino acid sequences ^67^FLEDR^71^ shared by *HLA-DRB* was associated with ATA positive and dcSSc. Amino acid sequence ^71^TRAELDT^77^ shared by *HLA-DQB1* showed weak association in dcSSc patients with positive ATA (29). A higher prevalence of SSc has been identified in the Choctaw Indian population in comparison to non-full-blooded Choctaws, other Native Americans, as well as the general population (18, 19). Multiple genetic loci located on chromosome 6 near the HLA complex have been identified and may contribute to the high prevalence of disease (19). *HLA-B35*, *Cw4*, DRB1*1602, DQA1*0501, and DQB1*0301 are strongly associated with SSc in the Choctaw Indian population who present clinically with dcSSc, pulmonary fibrosis, and ATA positivity (18).

HLA-B*62 and HLA-DRB1*07 correlate with diffuse skin involvement while high skin scores correlate with HLA-DRB1*11 (17). HLA-B*62 and HLA-Cw*0602 has association with pulmonary fibrosis, while HLA-B*13 and HLA-B*65 with PAH (17). HLA-B*35 is associated with a high risk of developing PAH in systemic sclerosis by influencing the production of endothelin-1 (ET-1) and decreasing endothelial nitric oxide synthase (eNOS) (18, 20–22). HLA-G is expressed in skin of patients with systemic sclerosis. Its presence is associated with having lower vascular cutaneous ulcers, telangiectasias, and inflammatory arthropathy (23).

## Non-HLA-Associated Genes

Multiple studies including GWAS, meta-analysis, and recently immunochip array analysis have repeatedly shown that modifications in *CD247*, interferon regulatory factor 5 (*IRF5)*, and signal transducer and activator of transcription protein 4 (*STAT4*) genes are associated with SSc susceptibility (Table 3). Many autoimmune disorders share a common genetic background. Both systemic lupus erythematosus (SLE) and SSc share many clinical features and genetic components. Disease sample size and lack of statistical power limits the ability to determine which genes may contribute to autoimmunity. Combined analysis of different autoimmune diseases increase sample size and allows for statistical power to identify genetic variants that effect disease. Using a GWAS pan-meta-analysis approach allows for the detection of new genetic susceptibility loci, as determined by Martin et al. (47). In the Martin et al. study, GWAS pan-meta-analysis approach for SSc and SLE identified and validated three new susceptibility genes for SSc [*KIAA0319L*, paraxylene–orthoxylene domain containing serine/threonine kinase (*PXK*), and *JAZF1*] (47). Genes related to cellular response to IFNγ and the nervous system was overrepresented in both SLE and SSc. In SSc, genes related to cell signaling, migration, and adhesion were over-represented (47). In this section of the review, we will discuss Non-HLA-associated genes reported to be associated with SSc. In Table 3, we have listed the non-HLA SSc-associated genes in order of decreasing SSc sample size analyzed.

**Table 3:** **Non-HLA genes associated with SSc listed from largest to smallest SSc population analyzed**.

No.	Non-HLA-associated genes	Other diseases identified	Study type	Population (*n* = SSc)	Polymorphism/SNPs (OR)	Disease association	Proposed function	Reference
1	*BANK1*	SLE	Case–control meta-analysis cohort	European ancestry (21,568)	rs10516487*G (1.12), rs17266594*T (1.14), rs3733197* A (0.73), AA (0.41), AG (0.85)	dcSSc ATA+	Mobilization of calcium from intracellular stores in B-cell receptor	(35–37)
2	*IRF5*	SLE, RA, UC	GWAS meta-analysis immunochip array case–control cohorts	European and Asian ancestry (15,251)	rs2004640 (0.84), rs2004640*TT (1.56), rs10488631 (1.63), rs4728142 (1.22)	dcSSc, lcSSc, ATA, ACA, interstitial lung, longer survival mild PF	Regulate IFN-gene expression and inflammatory cytokine production. Stimulate TLR expression	(38–42), (42–44), (14, 45, 46)
3	*PXK*	SLE	Immunochip pan-meta-analysis GWAS	European ancestry (12,685)	rs2176082 (1.21), rs4681851 (1.58)	SSc, ACA	Degradation and trafficking of epidermal growth factor	(14, 47)
4	*STAT4*	SLE, RA, primary biliary cirrhosis	GWAS immunochip meta-analysis cohort, case–control cohorts	Multi-ethnic ancestry (10,696)	rs7574865 (1.29), additive effect with IRF5 rs2004640 (1.72-2.752), rs11889341 (1.33), rs7574865*, TT vs. GG (0.49), TT vs. TG (0.48), TT + TG vs. GG (0.74), T vs. G (0.72), rs10168266*, CC (0.69), T (1.44)	lcSSc, ACA, fibrosing alveolitis, increased in patients who carry TBX21 CC genotype, dcSSc, ATA, pulmonary fibrosis	T-cell signaling and differentiation; signaling IFN1; regulate cytokine signals	(38, 48–54)	
5	*PTPN22*	DM-1, RA, SLE	Meta-analysis case–control cohorts	Multi-ethnic and European ancestry (10,204)	PTPN22 CT/TT (2.21) higher risk of SSc than PTPN22 CC (1.70), PTPN22, C1858T [rs2476601* T (1.15)], [1858 T (1.147)], 1858 C	ATA and ACA, ACA, protective	T-cell receptor signaling	(55–58)
6	*TNFSF4*	SLE	Meta-analysis cohort	European ancestry (10,093)	rs1234314 (1.15), rs12039904 (1.18), rs2205960*AA (1.33), rs844648 (1.10), rs844644 (0.91)	dcSSc, lcSSc and ACA+, protective in all sub-groups of SSc except ARA+	B-cell proliferation and differentiation T-cell stimulation and survival	(48, 59–61)
7	*BLK*	SLE	Case–control meta-analysis	European and Asian ancestry (9,305)	rs2736340 (1.27), rs13277113 (1.16), *C8orf13-BLK*, and *BANK1* additive effect, *FAM167-BLK*	dcSSc, dcSSc, dcSSc, lcSSc	Disruption in B-cell gene expression and abnormal NFκB signaling	(48, 62–64)
8	*IL-2*, *IL-2RA*	SSc	Case–control cohort	European (7,516)	IL-2: rs6822844 (0.86), rs907715 (0.91), rs2069762*A- (1.06), rs6822844*T- (0.86), rs683545*G (0.93), rs907715*T (0.91); IL-2RA genes: rs11594656, rs2104286 (1.30), rs12722495	SSc, lcSSc, dcSSc, and lcSSc when ACA+	T-cell proliferation and turning off T-cell response	(65, 66)
9	*DNASE1L3*	SLE, RA	Immunochip	European ancestry (7,169)	rs35677470 (2.03)	ACA	Defective apoptotic DNA breakdown	(14, 46)
10	*JAZF1*	SLE	GWAS pan-meta-analysis	European ancestry (6,835)	rs1635852 (1.13)	SSc	Repress transcription	(47)
11	*KIAA0319L*	SLE, dyslexia	GWAS pan-meta-analysis	European ancestry (6,835)	rs2275247 (1.49)	lcSSc	Protein coding	(47)
12	*IL-12R*β*2*	Psoriasis, Behcet’s disease, primary biliary cirrhosis	GWAS	European ancestry (6,250)	rs3790567 (1.17), rs2305743* A/G (0.81), rs8109496* C/G (0.82), rs436857* A/G (0.81), rs11668601* C/T (0.84)	SSc	Stimulates IFN production and TH1 differentiation	(67)
13	*IRF8*	SLE	Cohort meta-analysis	European and Asian ancestry (6,201)	rs11642873 (0.75), rs2280381 (1.36)	lcSSc	Regulate IFN-gene expression and inflammatory cytokine production. Stimulate TLR expression	(24, 68)
14	*CD247*	SLE	GWAS	European ancestry (6,080)	rs2056626 (0.78)	SSc-G minor allele protective effect	T-cell signaling and activation	(49, 69)
15	*ATG5*	SLE	Immunochip	European ancestry (5,850)	Intron rs9373839* G (1.19)	SSc	Autophagy vesicle formation	(14)
16	*IL-12A-SCHIP*	Primary biliary cirrhosis, idiopathic pulmonary fibrosis	Immunochip	European ancestry (5,850)	rs77583790 (2.81), intergenic between SCHIP1 and IL-12A	LcSSc	Stimulates IFN production and TH1 differentiation	(14)
17	*CSK*	SLE, RA	GWAS	European ancestry (5,270)	rs1378942 (1.2)	SSc	Cell regulation, differentiation, migration, and immune response	(70)
18	*PSD3*	Hepato-cellular carcinoma	GWAS	European ancestry (5,270)	rs10096702 (1.18)	SSc	Binding protein for signal transduction	(66)
19	*IL-12R*β*1*	Psoriasis, Behcet’s disease, primary biliary cirrhosis	Immunochip	European ancestry (5,052)	rs2305743 (0.81), rs436857	SSc	Stimulates IFN production and TH1 differentiation	(71)
20	*IRAK1*	SLE	Cohort meta-analysis	European ancestry (4,873)	rs1059702*TT (1.43)	dcSSc, ATA, SSc-related fibrosis alveolitis	Influence T-cell receptor signaling and TLR activation. Regulator of NFκB by way of X chromosome	(42, 48, 72)
21	*IL-21*	RA, SLE, DM-type 1, Graves” disease, celiac disease	Case–control	European ancestry (4,493)	rs6822844 (0.86), rs907715 (0.91), allelic combination: rs2069762* A- (1.06), rs6822844* T- (0.97), rs6835457*G- (0.93), rs907715* T (0.91), rs6822844* T (0.84)	lcSSc, ACA+, global SSc, dcSSc, lcSSc, protection: SSc, lcSSc, and ACA+	B-cell differentiation and regulates TH17 development	(65)
22	*TNIP1*	RA, SLE, psoriatic arthritis	GWAS cohort	European ancestry (4,389)	rs9275224 (0.69), rs6457617 (0.69), rs9275245, rs3130573 (1.12) located in *PSORC1C1* gene, rs2233287 (1.19), rs4958881 (1.19), rs3792783 (1.19)	Global SSc, global SSc except ACA+: dependent on HLA II, global SSc	Negative regulatory of NFκB	(48, 73, 74)
23	*MIF*	SLE, psoriatic arthritis, Lofgren’s syndrome, inflammatory bowel disease	Cohort	European ancestry (4,286)	MIF-173 (1.10)	dcSSc	Activates innate immunity and sustains cellular response	(60, 75)
24	*NFk*β*1*	Behcet’s disease, Grave’s disease, Hashimoto thyroiditis	GWAS	European ancestry (4,156)	rs1598859 (1.19)	SSc	Controls inflammation, transcription regulator	(66, 76)
25	*CD226*	SLE, DM-type 1, multiple sclerosis	Cohort	European ancestry (4,131)	rs763361* T (1.02), rs34794968 (0.90), rs727088 (1.02)	dcSSc, ATA+, ILD, pulmonary fibrosis	Co-stimulator of T cells and T-cell adhesion	(77–80)
26	*PPAR*γ**	RA, psoriatic arthritis, DM-type 1	GWAS	European ancestry (3,989)	rs310746 (1.28)	SSc	Blocks TGF-β, mediated fibrosis	(81–83)
27	*ITGAM*	SLE	Case–control cohort meta-analysis	European ancestry (3,735)	rs1143679* A (1.12)	SSc	Regulates neutrophil and monocyte cell activation and adhesion. Phagocytosis of complement-coated particles	(47, 84, 85)
28	*TNFAIP3*	SLE, RA, celiac disease, multiple sclerosis	Cohorts case–control	European and Asian ancestry (3,365)	rs5029939* G (2.08), rs6932056 (1.69), rs117480515* A (3.20), rs117480515* A (3.94)	dcSSc, fibrosing alveolitis, PAH, SSc, polyautoimmune subset	Regulate immune system signaling by regulating ubiquitin	(43, 48, 68, 86)
29	*IRF7*	SLE	Case–control	European ancestry (2,316)	rs1131665 (0.87)	ACA	Regulate IFN-gene expression and inflammatory cytokine production. Stimulate TLR expression	(87)
30	*IL-23R*	Inflammatory bowel disease, psoriasis, AS	Case–control	US – multi-ethnic and European ancestry (2,134)	rs11209026*GG (0.81), (Arg381 Gln), rs11465804*TT (0.83)	ATA, protection against PAH, dcSSc, ATA, protection against PAH	Promotes TH17 expansion	(28, 88, 89)
31	*TLR2*	Inflammatory bowel disease, multiple infections	Cohort	European ancestry (1,170)	rs5743704 (2.24), (Pro63 His)	dcSSc, ATA+, PAH	Pathogen recognition and direct immune response	(90)
32	*CD87(UPAR)*	Vascular disease, paranasal disease	Cohort	European ancestry (732)	rs344781*GG (1.96)	SSc-associated digital ulceration, PAH, ACA, dcSSc, lcSSc	Promotes extracellular matrix and vascular remodeling	(91)
33	*PLD4*	RA	Cohort	Japanese (730)	rs2841277 (1.29), rs2841280* G (1.29), (minor)	lcSSc, dcSSc, protective SSc	Phagocytosis of microglia	(68)
34	*MMP-12*	Kidney disease, skin diseases, arthro-sclerosis	Cohort	Italian ancestry (250)	rs2276109*AA (2.44)	dcSSc, lcSSc, ATA+, pulmonary fibrosis	Inhibits endothelial cell proliferation and angiogenesis	(92)

### Autophagy protein-5

Autophagy protein-5 (ATG5) is an ubiquitin ligase protein that assists in autophagosomal elongation that mediates pathogen clearance; allowing for the degradation of unwanted cytoplasmic material. It has a role in the development of both the innate and adaptive immune system (14). Variations in *ATG5* are associated with susceptibility in SLE and childhood and adult asthma (14). Variants located within *ATG5* intron rs9373839 G minor allele have been identified as SSc susceptibilities (14). The location of this variant may suggest that distant genes may affect downstream the function of *ATG5*.

### B-cell scaffold protein with ankyrin repeats 1

B-cell scaffold protein with ankyrin repeats 1 (BANK1) exerts influence in B-cell receptor-induced calcium mobilization from intracellular (IC) stores. It has been identified in SLE as a susceptibility gene. There is an increased risk for developing SSc with *BANK1* haplotype G–C compared to A–T haplotype (35). *BANK1* variants rs3733197 G alleles, rs10516487, rs10516487*G, and rs17266594*T are strongly associated with diffuse dcSSc and ATA autoantibodies (36).

### B-lymphocyte kinase

B-lymphocyte kinase (BLK) encodes B-cell signal transducer and functional variant *C8orf13-BLK*. Disruption in *BLK* may result in abnormal B-cell gene expression and altered NFκB signaling (48). *C8orf13-BLK* has been identified in multiple studies as a risk gene for SSc (62–64). *C8orf13-BLK* variant rs2736340 and rs13277113 are associated with SSc and dcSSc (63). An additive effect between *C8orf13-BLK* and *BANK1* increases susceptibility to dcSSc (62). Two haplotype blocks (*FAM167A* and *BLK*) have also been identified. Allele rs13277113*A in the *BLK* block is significantly associated with SSc (64). This association was observed despite autoantibody profile or disease classification (dcSSc or lcSSc) (64).

### CD247

*CD247* encodes T-cell receptor zeta (CD3ζ), which functions in the assembly of TCR–CD3 complex and its transport to the cell surface, thereby playing a crucial role in cell signaling (49). Variants of *CD247* may lead to impaired immune response and dysregulation of T-cell activation. *CD247* has been associated with susceptibility to SLE. *CD247* rs2056626 (in addition to *IRF5*, MHC, and *STAT4*) were identified as susceptibility genes for SSc in multiple studies. The G minor allele of this variant has a protective effect (49, 69). This variant was not found to have an association with SSc or disease subtypes in a Hans Chinese cohort, suggesting that the association may be ethnicity-dependent (113).

### c-SRC tyrosine kinase

c-SRC tyrosine kinase (CSK) is important for cell regulation, differentiation, migration, and immune response. *CSK* inactivates src kinases by phosphorylating tyrosine at the C-terminus. In fibrosis, srk kinases regulate FAK needed for integrin signaling and fibroblast adhesion to extracellular matrix (ECM). Incubating fibroblasts with inhibitors of *CSK* decreases COLIAI and COLIA2. Polymorphisms in *CSK* prevent or inhibit the phosphorylation of src leading to fibrosis (70). Polymorphism in the intron of the *CSK* gene is associated with SSc. Variant rs1378942 is associated with overall SSc (70).

### Deoxyribonuclease 1-like 3

A member of the human DNase 1 family, deoxyribonuclease 1-like 3 (DNASE1L3) is secreted by macrophages and is found in the liver and spleen (14). During apoptosis, *DNASE1L3* has a role in the fragmentation of DNA. It also generates double-strand breaks in immunoglobulin-encoding genes. In regards to autoimmune susceptibility, *DNASE1L3* is found to be associated with susceptibility to SLE and rheumatoid arthritis (RA). Using the immunochip array, *DNASE1L3* SNP rs35677470 was identified as a risk for SSc and ACA positivity. These authors identified a substitution in amino acid Arg to Cys at position 206 on exon 8 of *DNASE1L3* protein resulted in the loss of a hydrogen bond. The amino acid substitution in this position may cause the protein to become inactive suggesting a potential role for SNP rs35677470 in autoimmunity due to defective apoptotic DNA breakdown (14).

## Interferon-Regulated Genes and PAH

Multiple studies using GWAS, meta-analysis, and immunochip analysis assays have confirmed the involvement of IFN in SSc susceptibility. The identification of multiple variants in IFN genes in association with SSc, SSc lung disease, and SSc mortality highlights the significance of the IFN pathway in the development and progression of SSc. IFN modulate differentiation, survival, proliferation, and cytokine production by T and B cells and dendritic cells. IFN stimulate the expression of toll-like receptors (TLRs) 3, 7, and 9. IFN genes were overexpressed in peripheral blood mononuclear cells (PBMCs) from patients with SSc and SLE. Higher IFN scores correlated with ATA, anti-U1RNP, lymphopenia, and IFNα/IFNβ receptor 2 (*IFNAR2*) missense mutation rs7279064 GG or GT (114). Other variants in the IFN pathway have also been well established in SSc. Polymorphisms in *IRF5*, *IRF7*, and *IRF8* have been identified. *IRF5* mediates IFN activity and is an important inflammatory signaling pathway. Polymorphisms in IRF5 are associated with SLE, RA, ulcerative colitis, and others. Regulation in immune reaction to infections by *IRF5* is activated by TLRs 7 and 9. In SLE, *IRF5-transportin-3* gene (*TPO*) rs4728142 correlates with *IRF5* expression leading to increased binding of zinc-finger BD 3 (ZBTB3) affecting both RNA transcription and DNA binding (115). In SSc, *IRF5* rs200460 is associated with dcSSc, lcSSc, ATA, and ACA. The strongest association is with ATA and ILD (38). It is linked to overall mortality independent of disease type or serology (39). A Han Chinese cohort of 424 SSc patients identified rs2004640*TT genotype as being significant in this population. This variant is associated with pulmonary fibrosis and ATA positivity (40). *IRF5* rs4728142 is predictive of longer survival and milder pulmonary fibrosis. The association is independent of age of disease onset, autoantibody profile, or disease type (41). *IRF7*: Interferon regulatory factor 7 (IRF7) activates type IFN genes in response to DNA/RNA immune complexes and viral infections. *IRF7* associates with susceptibility to SLE. Multiple variants in the *IRF7* genes confer susceptibility to SSc. *IRF7* rs1131665 is associated with SSc-associated ACA positivity. The variants identified were replicated in a Spanish cohort (87). *IRF8*: Multiple studies have identified *IRF8* association with SSc and rs11642873 with lcSSc (24). *IRF8* rs2280381 has been identified as SSc susceptible gene in a Japanese cohort (68).

Attention has focused on the possible contribution of the immune system to pathogenetic processes in PAH, especially innate immunity and IFNs (116, 117). Type I IFNs are implicated by the association of use of IFNα in the treatment of hepatitis and of IFNβ in the treatment of multiple sclerosis (MS) with development of PAH (118, 119). Diseases in which there is an “IFN signature” (such as SLE, SSc, and infection with HIV) are associated with development of PAH (120–124). Furthermore, IFNα and IFNγ added to cultures of human pulmonary artery smooth muscle cells (PASMC) primed with TNFα or to cultures of human lung MVEC or human lung fibroblasts, cause release of the potent vasoconstrictor, ET-1, and of IFN-inducible protein-10 (IP-10) (117). In a series of 128 SSc patients with PAH and 35 patients with no PAH, the SSc patients with PAH had higher levels of IP-10 and ET-1 in their sera compared to SSc patients without PAH or compared to healthy controls. More SSc patients with PAH had detectable levels of IFNα and IFNγ in their sera than SSc patients without PAH (117). In this series of SSc patients, levels of TNFα, IL-12p70, IL-6, IL-1α, and IL-8 were significantly higher in sera in SSc patients with PAH when compared to SSc patients without PAH (117). Additional studies of this patient group revealed that serum levels of IP-10 in the SSc-PAH patients correlated with pulmonary vascular resistance, and levels of brain natriuretic peptide in serum, and serum IP-10 levels in the SSc-PAH patients inversely correlated with cardiac index and 6-min walks test (117). Sections of lung from patients with idiopathic PAH (IPAH) or with SSc-PAH expressed higher levels of type I interferon receptor 1 (IFNR1) in endothelium, smooth muscle layer, vascular interstitium, and in intravascular inflammatory cells as assessed by immunohistochemistry and Western blotting (117). While the above studies strongly implicated type I IFN as playing a pathogenic role in SSc-PAH and IPAH, further evidence was substantiated in the type I interferon α receptor 1 knockout mouse which was found to be resistant to experimental hypoxic PAH induction. These mice did not have elevated serum levels of ET-1 when compared to wild-type (WT) control mice (117). Analysis of PBMC from patients with SSc revealed CD169/sialoadhesin (*Siglec-1)* and other IFN-regulated genes were overexpressed in patients with dcSSc, whereas patients with lcSSc with PAH overexpressed *IL-13RA1*, intercellular adhesion molecule-1 (*ICAM-1*), *C–C chemokine receptor type 1 protein or gene* (*CCR1*), *JAK2*, and *melanocortin receptor 1 (MCR1)* (123, 125, 126). IL-13 was also elevated to higher levels in sera of patients with lcSSc with PAH, and *MCR1* was induced on CD14^+^ monocytes suggesting monocytes are activated in lcSSc patients with PAH of an alternative (i.e., IL-4/IL-13) rather than classical [i.e., IFNγ/lipopolysaccharides (LPS)] pathway (123). The identification of multiple IFN genes having association in SSc, SSc lung disease, and mortality highlights the significance of the IFN pathway in the development and progression of SSc.

### Interleukin-1 receptor associated kinase 1

Interleukin-1 receptor associated kinase 1 (*IRAK1*) gene is located on the Xq28 and is in the same haplotypic block with methyl-CpG-binding protein 2 gene (*MECP2*). *IRAK1* encodes a serine/threonine protein kinase that regulates NFκB through T-cell receptor signaling and TLRs/IL-1R activation. It also plays a role in IFN induction. IRAK1 has been identified in SLE as a susceptibility gene (42, 72). In SSc, *IRAK1* rs1059702*TT is associated with dcSSc, SSc-related fibrosing alveolitis, and ATA positivity (42, 72). The presence of the T allele may contribute to disease severity, and presence of *MECP2* rs17435 may explain the association of *IRAK1* variant rs1059702 with this subset (42, 72).

### IL-2/IL-12 genetic susceptibility

Variants in interleukin-2 receptor α (IL-2A), *IL-12R* (*IL-12R*β**1 or *IL-12R*β*2*) have been reported to be associated with SSc. *IL-2* plays a role in immune system homeostasis and self-tolerance. It facilitates B-cell immunoglobulin production and induces natural killer cell proliferation and differentiation (65). The binding of *IL-12* to its receptors stimulates IFN production and promotes TH1 differentiation. IL-12 signals through STAT pathway and a defect in either *STAT4* or *IL-12R* could influence SSc pathogenesis. Variant SNP rs77583790 found in the intergenic region between *SCHIP1* and *IL-12A* was found to be associated with lcSSc (14). *IL-12R*β*1* and *IL-12R*β*2* recruit tyrosine kinases and activate transcription of other genes. Polymorphisms in IL-*12R*β*1* and *IL-12R*β*2* have been identified in psoriasis, Behcet’s disease, and primary biliary cirrhosis (67). Two studies were conducted to investigate the role of *IL-2* in SSc. ILR2 gene variants: rs11594656, rs2104286, and rs12722495 were associated with SSc, lcSSc, and ACA positivity. The associations are strongly dependent on ACA since removal of ACA from the analysis resulted in loss of association, and the strongest association with ACA positivity was with rs2104286, with associations of the other IL-2 RA gene variants being lost after conditioning to rs2104286 (66). Polymorphism in rs2104286 has the strongest association with ACA while rs6822844 and rs907715 have association with SSc and lcSSc (66). *IL-12R*β*1* rs2305743 and rs436857 were found to be associated with SSc (71). Polymorphisms in these receptors may affect the binding of transcription factors decreasing the expression of IL-12. *IL-12R*β*2* rs3790567 is associated with SSc. *IL-12R*β*2* gene maps close to the *IL-23* coding region, the association between rs3790567 was not found to be dependent on *IL-23* (67). *IL-2/IL-21: IL-21* affects the innate and adaptive immune response playing a role in the differentiation of B cells into plasma cells and regulation of TH17 development (65). Polymorphism in the *IL-2/IL-21* region is associated with lcSSc and global SSc. *IL-2/IL-21* variant rs682284 is strongly associated with multiple autoimmune diseases and is considered an autoimmune susceptibility locus (127). The rs907715 minor allele and rs682284 have association with SSc. Variant rs6822844 influences lcSSc and ACA positivity (65). The allelic combination of rs2069762*A–rs6822844*T–rs6835457G–rs907715*T is associated with dcSSc and lcSSc (65). The T allele for rs6822844 acts as a protective for lcSSc and ACA positivity.

### IL-23

*IL-23* promotes the expansion of TH17. *IL-17* and *IL-23* are elevated in the plasma of SSc patients (28). Polymorphism in *IL-23R* is associated with SSc and ATA positivity. *IL-23R* variant rs11209026*GG (Arg381 Gln variant) has association with ATA positivity and rs11465804*TT is associated with dcSSc and ATA positivity. The major alleles rs11209026*G and 11465804*T were decreased in patients with PAH, suggesting that the major allele is protective against PAH (28).

### Integrin αM

Integrin αM (ITGAM) β2 is a leukocyte-specific integrin that regulates neutrophil and monocyte cell activation and adhesion. It allows for phagocytosis of complemented-coated particles. Deficiency in ITGAM results in increased IL-6 production by antigen-presenting cells (APC) (128). Pooled meta-analysis, subsequent independent meta-analysis, and GWAS looking at shared risk polymorphisms for SLE and SSc confirmed *ITGAM* variant rs1143679 were associated with susceptibility to SSc (47, 84, 128).

### Juxtaposed with another zinc-finger 1

Juxtaposed with another zinc-finger 1 (*JAZF1*) encodes a nuclear protein with zinc-fingers that functions to repress transcription. It has been associated with bone morphogenesis and CI deposition (47). *JAZF1* has been identified as an SLE-associated locus, and a recent GWAS pan-meta-analysis has confirmed *JAZF1* rs1635852 association with SSc (47).

### KIAA03192L

KIAA03192L has been identified in polycystic kidney disease and dyslexia as a disease susceptibility gene. It is expressed in macrophages and natural killer cells in mice and in CD33^+^ myeloid cells and CD14^+^ monocytes in humans. *KIAA03192L* is overexpressed in PBMCs of SLE patients. In SSc, *KIAA03192L* variant rs2275247 is associated with lcSSc (47).

### Protein tyrosine phosphatase non-receptor type 22

Protein tyrosine phosphatase non-receptor type 22 (*PTPN22*) plays a critical role as a gatekeeper for T-cell receptor signaling. It encodes the protein tyrosine phosphatase lymphoid tyrosine phosphatase in T-cells and acts to inhibit T-cell signaling through dephosphorylation of substrates. Polymorphism in *PTPN22* has been associated with type 1 DM, RA, and SLE. Earlier studies looking at the relationship between *PTPN22* and SSc failed to show an association between *PTPN22* and SSc (129, 130). Larger studies in SSc patients showed association with *PTPN22* Ct/TT genotypes with both ATA and ACA positivity. The T allele associated with ATA positivity and the CC genotype with both ACA and ATA positivity (55). Meta-analysis confirmed *PTPN22* rs2476601*T and the minor allele 1858T are associated with SSc and ACA positivity (56, 57). Haplotype 1858C allele was protective in a French cohort (58).

### Paraxylene–orthoxylene (phox homology) domain containing serine/threonine kinase

Paraxylene–Orthoxylene domain containing serine/threonine kinase is a protein that plays a role in the ligand-induced internalization, degradation, and trafficking of epidermal growth factors. Variation in PXK is association with SLE susceptibility where it is found to alter B-cell receptor internalization (131). *PXK* rs2176082 and rs4681851 are associated with SSc and rs2176082 has association for ACA positivity. The association of rs2176082 is related to *DNASE1L3* (14, 47).

### Signal transducer and activator of transcription protein 4

Signal transducer and activator of transcription protein 4 is critical for T-cell signaling and differentiation (132–134). *STAT4* is involved in effecting a Th1 cytokine response by transmitting signals from IL-2, IL-12, and IL-23 receptors and in signaling after type 1-IFN engages its receptor (135, 136). The role of *STAT4* in fibrosis was assessed in scleroderma mouse models. To assess the contribution of *STAT4* to bleomycin (BLM)-induced skin fibrosis and fibrosis of skin in (tight skin) Tsk-1/+ mice, BLM was injected for 3 weeks into *STAT4^–/–^* and *STAT4*^+/+^ mice. *STAT4^–/–^* mice were crossed with Tsk-1/+ mice, and skin fibrosis was assessed (137). The deletion of *STAT4* significantly reduced skin fibrosis in the BLM model but not in the Tsk-1/+ model (137). In the BLM model, it was noted that there were decreased numbers of inflammatory cells including T cells and proliferating T cells and decreased quantity of IL-6, IL-2, TNFα, and IFNγ in lesional skin of *STAT4^–/–^* vs. *STAT4*^+/+^ mice (137).

Signal transducer and activator of transcription protein 4 is considered an autoimmunity loci since its association has been firmly confirmed in SLE, RA, primary biliary cirrhosis, and SSc (48). SNP rs7574865 is associated susceptibility to lcSSc and ACA positivity (50, 51). SNP rs7574865 and rs10168266 were associated with dcSSc, ATA positivity, and pulmonary fibrosis in a Chinese cohort (52). Variant rs7574865*T allele has an additive effect with IRF5 rs2004640 seen in fibrosing alveolitis (38). Gene–gene interactions between *STAT4* and polymorphism in the transcription factor T-bet show increased susceptibility to SSc. Transcription factor *T-bet* [(T-box expressed in T cells) (*TBX21*)] is an important transcriptional activator of Th1 differentiation effecting Th1/Th2 balance. Polymorphisms in *TBX21* have associations with RA, asthma, and type 1 DM. TT genotype of *TBX21* variant rs11650354 confers susceptibility to SSc in a recessive manner while *STAT4* variant rs11889341 A allele is associated with an increased risk of SSc in a dominant pattern. *STAT4* genotype increased the SSc risk in the presence of *TBX21 CC* genotype (53). Plasma levels of circulating IL-6 and TNF were increased in SSc patients who carry the *TBX21 CC* genotype where as those who carry the TT genotype show increased circulating IL-2 and IL-5 suggesting that patients who carry the CC genotype have a prominent pro-inflammatory cytokine profile (53). Gene expression profile from whole blood RNA of SSc patients suggest a role for type 1-IFN and pro-inflammatory cytokines in the CC genotype and of the T-cell pathway in the TT group (53).

### Tumor necrosis factor alpha-induced protein-3

Tumor necrosis factor alpha-induced protein-3 (*TNFAIP3*) encodes ubiquitin-modifying protein A20 and has a critical role in the regulation of immune signaling pathways.

Polymorphism in *TNFAIP3* is associated with SLE, RA, and celiac disease. *TNFAIP3* rs117480515, rs5029939*G allele, and rs6932056 carry an increase of susceptibility to SSc (43, 68). TNFAIP3 SNP and rs5029939*G is associated with dcSSc, fibrosing alveolitis, and PAH (43). The rs117480515*A allele is associated with SSc polyautoimmune subset (86).

### Tumor necrosis factor superfamily member 4 gene

Tumor necrosis factor superfamily member 4 gene (*TNFSF4*) encodes for the T-cell co-stimulatory molecule, OX40 ligand. *TNFSF4* has a role in B-cell proliferation and differentiation and T-cell proliferation. Ox40–OX40L promotes generation of Th2 cytokines. It has been identified as a susceptibility gene for SLE. TNFSF4 SNPs variant rs1234314, rs2205960, rs844648, rs12039904, rs1234317, and rs10912580 have been identified as susceptibility genes in SSc and are associated with lcSSc- and ACA-positive SSc patients in multiple French European studies (120–122). The minor allele rs1234314 has association for lcSSc, ACA, and ATA, while rs844648 confirmed association with dcSSc and ARA. Variant rs844648 was found to be protective in all SSc sub-groups except ARA+. In women, rs2205960*TT/GT and rs844648*AA associates with increased risk for SSc (59). These studies suggest *TNFSF4* as a susceptibility gene for SSc.

### TNFAIP3 interacting protein-1

TNFAIP3 interacting protein-1 *(TNIP1)* gene interacts with A20 binding protein (BP) and inhibits TNF-induced NFκB-dependent gene expression; thereby negatively regulating NFκB. Mutations in this gene have been associated with RA, SLE, and psoriatic arthritis. *TNIP1* gene and protein expression was reduced in lesional skin tissue and cultured fibroblasts from SSc patients. *In vitro*, *TNIP1* had inhibitory effects on inflammatory cytokine-induced CI production (73). *TN1P1* SNP rs2233287, rs4958881, and rs3792783 are associated with global SSc (74). A two-staged GWAS showed strong linkage disequilibrium in the *HLA-DQB1* gene: rs9275224, rs6457617, and rs9275245. Within the MHC region, there was association with rs3130573 located in the *PSORC1C1* gene. *PSORS1C1* also show susceptibility in global SSc except for ACA positivity patients but this association is dependent on HLA class-II (74).

### CD87 (UPAR)

Urokinase-type plasminogen activator receptor (UPAR) promotes ECM and vascular remodeling. It regulates growth factor activation and is responsible for cell adhesion, migration, and proliferation (91). *UPAR* rs344781*G allele is associated with SSc-related digital ulcers, pulmonary artery hypertension, ACA positivity, and lcSSc (91). Genotype rs344781*GG is identified as an independent risk factor for SSc-related digital ulcers and PAH (91). *CD226*: acts as a co-stimulator of T cells and plays a role in T-cell adhesion. It is expressed on NK cells, monocytes, platelets, and B and T cells (77). It has been correlated with susceptibility to SLE, type 1 diabetes, thyroid disease, and MS (78–80). In SSc, the *CD226* T allele of rs763361 may contribute to disease severity due to its association with multiple SSc subsets including dcSSc, ATA positivity, and ILD (80). *CD226* haplotype SNP rs763361, rs34794968, and rs727088 correlates with pulmonary fibrosis (77). *MIF*: Macrophage migration inhibitory factor (MIF)-173 acts upstream, activates innate immunity, and sustains cellular and inflammatory responses. MIF induces endothelial adhesion and induces fibroblast proliferation that may contribute to vasculopathy (135). MIF-173 is lower in lcSSc. *In vitro*, *C7 MIF* encoded fibroblasts produced more MIF than non-stimulated fibroblasts (75). In an American and European study that included 3,800 SSc patients, MIF was found to have higher association with dcSSc compared to controls and lcSSc (75, 138). *MMP-12*: matrix metalloproteinase-1 (MMP-1) rs2276109*AA genotype has significant association in dcSSc, lcSSc, ATA positivity, and pulmonary fibrosis in an Italian SSc population (92). *NFkB1* gene SNP rs1598859 is associated with overall SSc disease (70). *PLD4*: phospholipase D family member 4 (PLD4) was identified as a susceptibility gene for SSc in Japanese (68). *PPAR*γ: peroxisome proliferation-activated receptor gamma (*PPAR-*γ) when engaged by ligands of different types blocks transforming growth factor (TGF)-β mediated fibrotic responses *in vitro* in cultured fibroblasts and in various fibrotic animal models *in vivo* (81, 82). PPARG rs310746 is associated with SSc (83). *PSD3*: involved in signal transduction pathways and IC signaling. Polymorphism in the *PSD3* gene rs10096702 is associated with overall SSc (70). *TLR2*: subcutaneous injections of TLR ligands into the skin of SSc results in a significant inflammatory reaction resulting in SSc skin changes (90). *TLR2* *pro63* His is associated with dcSSc, PAH, and ATA positivity (90). *TLR5* and 10 expression were increased in SSc fibroblasts *in vitro* and *in vivo* (139).

#### Vascular Related Genes

Endothelin-1 is one of three isoforms and is synthesized by vascular endothelial (VE) cells, fibroblasts, bone marrow mast cells, neutrophils, macrophages, and cardiac myocytes (140). Various triggers induce synthesis of ET-1 including TGF-β and other growth factors, cold exposure, low shear stress, hypoxia, and angiotensin II (140); but its synthesis is reduced by nitric oxide (NO), natriuretic peptides, increased blood flow, and prostacyclin (141). ET-1 is also degraded by MMP-1, which is reduced in SSc (140). Two types of receptors for ET-1 (ET_α_ and ET_β_) are variably expressed on endothelial cells, vascular smooth muscle cells, adventitial fibroblasts, tissue fibroblasts, neutrophils, mast cells; and monocytes and ET receptor engagement on these cells triggers a variety of pro-inflammatory or fibrotic response, including vasoconstriction of vasculature (140). ET-1 increases surface expression of ICAM-1 on fibroblasts, stimulates CI synthesis, promotes formation of myofibroblasts, and facilitates binding of T cells to fibroblasts (140, 142). ET-1 acts as a downstream mediator of TGF-β, and its induction by TGF-β in fibroblasts is via small mother against decapentaplegic (Smad)-independent signaling that involves c-Jun N-terminal kinase (JNK) and activin receptor-like kinase (ALK)5 pathways (143). Polymorphisms of ET-1 receptors are associated with SSc. For example, there is an association of *EDNRB* polymorphisms and dcSSc and *EDNR-A* polymorphism with anti-RNA polymerase autoantibodies in SSc (140). Polymorphisms were also described in the promoter of the NOS2 gene that confers susceptibility to PAH in SSc (144). Potassium voltage-gated channel shaker-related subfamily 5 (*KCNA5*) has a role in the regulation of vascular tone. It is inhibited by hypoxic conditions leading to vasoconstriction. *KCNA5* may have a protective role against PAH-associated SSc, this protective role was identified with variant rs10744676 (145).

## MicroRNAs

MicroRNAs are translational regulators of gene expression and also destabilize messenger RNAs (mRNAs) of target genes (146). MiRNAs are tissue- and cell type-specific short, single-stranded non-coding RNAs that function to modulate gene expression (Table 4). MiRNA bind to the 3′ untranslated region of mRNA of the target gene and mediate post-transcriptional regulation. Once bound, they either cause translational repression of the target gene or induce the degradation of the gene (147–149). In SSc, several miRNAs are associated with TGF-β and CI expression. In comparison to normal skin tissue, Zhu et al. (93, 147) found that skin from patients with lc and dc SSc expressed miR-21, miR-31, miR-146, miR-503, miR-145, and miR-29b. In these patients, miR-21 was increased in both tissue and fibroblasts whereas miR-145 and -29b were decreased. These miRNAs targeted the TGF-β pathway – including Smad7, Smad3, and COL1A1. TGF-β stimulation resulted in increased miR-21 expression and decreased expression of Smad7, while the upregulation of miR-145 was associated with a downregulation of Smad3 message. These same authors found that overexpressing miR-21 in fibroblasts decreased Smad7 but knocking down the expression of miR-21 increased Smad7 expression (93). miR-21 was also found to have increased expression in BLM-induced skin fibrosis. Reporter gene assay analyses revealed that the target gene for miR-21 is Smad7, while the target gene for miR-145 is *Smad3* (93, 94).

**Table 4:** **MicroRNAs in SSc**.

Micro RNA (miR)	Implications for SSc pathogenesis	Reference
miR-21 ↑ in SSc skin and fibroblasts	↑ by TGF-β, ↓ Smad7	(93, 94)
miR-145 ↓ in SSc skin and fibroblasts	Smad3 is its target gene	(93, 94)
miR-29b ↓ in SSc skin and fibroblasts	Suppressor of fibrosis	(94)
miR-30b ↓ in SSc skin and fibroblasts	Suppressor of PDGFR-β	(95)
miR-29a ↓ in SSc sera and skin	Suppressor of CI and CIII synthesis by fibroblasts, miR-29a is reduced by TGF-β, PDGF-β, and IL-4. Lower serum miR-29a associates with PAH	(96–98)
miR-196a ↓ in dcSSc sera	Expression of miR-196a reduced by TGF-β. May regulate CI synthesis. ↓ miR-196 = ↑ MRSS, ↑ digital pitting, and scars	(99, 100)
miR-150 ↓ in SSc fibroblasts and sera	Reduces fibroblast CI, pSmad3, and integrin expression	(101)
miR-7 ↓ in localized scleroderma skin and fibroblasts	Reduces CI synthesis by fibroblasts. Regulated by TSP2	(102, 103)
miR-let-7a ↓ in SSc and localized scleroderma skin and sera	Reduces CI synthesis by fibroblasts	(104)
miR-129-5p ↓ in SSc	Suppressor of COL1A1 expression in fibroblasts	(105)
miR-142-3p ↑ SSc in sera	May regulate αV integrin, which may recruit and activate small latent complex that regulates autocrine TGF-β activity	(106)
miR-92a ↑ in SSc sera and fibroblasts	May downregulate MMP-1	(107)
miR-21 ↑ SSc in fibroblasts	miR-21 is upregulated by TGF-β and decreases expression by Smad7	(93, 94)

Ninety-five miRNAs were analyzed in the sera of SSc patients and healthy controls. This analysis revealed that miR-30b was significantly downregulated in SSc patients and the modified Rodnan skin score (MRSS) inversely correlated with the level of miR-30b (95). Downregulation was also seen in the skin of scleroderma patients and BLM-treated sclerotic skin (95). Transfection studies showed that miR-30b affects platelet-derived growth factor/receptor (PDGFR)-β expression by suppressing this receptor (95). In their evaluation of 15 SSc patients and 15 normal subjects, Koba et al. (150) found that miR-206 and miR-21 were useful in distinguishing patients with SSc from normal subjects (150).

### miRNA-targeting CI

The expression of miR-196a was investigated in SSc both *in vitro* and *in vivo*. *In vivo* miR-196a was detected in the serum of SSc patients. Patients who had measurable lower levels of miR-196a had dcSSc compared to lcSSc. Lower levels of miR-196a was also associated with higher prevalence of pitting digital scars and more fibrotic skin as measured by MRSS (99). *In vitro*, the expression of miR-196a was normalized by TGF-β small interfering RNA (siRNA) in SSc fibroblasts, and the addition of miR-196a inhibitor to these fibroblasts resulted in the downregulation of CI. When the inhibitor was added to normal fibroblasts, there was an overexpression of CI (99). These results suggest that miR-196a may regulate CI expression.

Micro-RNA-29 (miR-29) is a TGF-β associated miRNA and is linked to fibrosis likely by interaction with several extracellular genes including ELN, FBN1, COL1A, COL1A2, and COL3A1 (151, 152). TGF-β/Smad3 signaling appears to negatively regulate miR-29 (153). Support for this relationship was the finding that in BLM pulmonary fibrosis mouse model, Smad3 was upregulated while miR-29 was downregulated in contrast to results with Smad3^–/–^ mice, which were protected from BLN pulmonary fibrosis and miR-29 was upregulated (153). In addition, therapeutic delivery of miR-29 to mice using Sleeping Beauty transposon-mediated gene transfer protected mice from developing BLM-induced lung fibrosis (153). MiR-29a has the ability to bind to the *3′UTR of COL1A1* and *COL1A2* (96, 154). Maurer et al. (97) found that miR-29a was strongly downregulated in SSc fibroblasts and skin sections when compared to healthy controls (97). SSc fibroblasts, in which miR-29 was overexpressed, exhibited decreased expression and protein levels of CI and CIII, while knockdown of miR-29 in normal fibroblasts increased CI production. Levels of miR-29 were reduced in normal fibroblasts when these fibroblasts were cultured with TGF-β, PDGF-β, or IL-4 (97). These studies confirm that miR-29a directly regulates CI. Serum levels of miR-29a were investigated to determine its potential role as a biomarker in SSc. In 61 patients with SSc, approximately 40% of which had dcSSc, miR-29a was found to be upregulated and not downregulated as expected in the serum of these patients. Patients with scleroderma spectrum disorder (SSD) are those who did not fulfill the ACR diagnosis criteria for SSc but who may develop scleroderma in the future. In these patients, miR-29a was downregulated compared to healthy controls, dcSSc, and lcSSc patients (96). Decreased serum levels of miR-29a may also be associated with higher right ventricular systolic pressure and PAH (96).

MiR-150 expression is decreased in SSc fibroblasts and sera. Normal fibroblasts that were transfected with miR-150 inhibitor had induced expression of type 1 CI, pSmad3, and integrin (101). Forced expression of miR-150 in SSc fibroblasts resulted in downregulation of CI, pSmad3, and integrin (101). In patient sera, lower expression of miR-150 correlated with severe clinical disease (101).

Skin and fibroblasts from localized scleroderma showed decreased levels of miR-7 compared to keloid skin and normal skin *in vivo* and *in vitro* (102). Normal fibroblasts that were transfected with miR-7 inhibitor exhibited upregulation of COL1A2 (102).

Skin and sera from SSc and localized scleroderma patients showed a downregulation of miR let-7a when compared to normal and keloid skin (104). CI was reduced by the overexpression and inhibition of miR let-7a in human and mouse skin fibroblasts (104). Intermittent overexpression of miR let-7a by intraperitoneal injections reduced dermal fibrosis in the BLM skin model (104).

MiR-129-5p is a regulator of *COL1A1* (154) and is downregulated in SSc (105). Nakashima et al. (105) found that, in their 20 patients with SSc, IL-17A expression was increased in the involved skin and sera, but IL-17R type A was decreased in SSc fibroblasts when compared to normal (105). IL-17A reduced protein expression of type I CI α1 chain [α1(I)] and connective tissue growth factor (CTGF). IL-17A also induced the expression of miR-129-5p (105). In the presence of IL-17A, miR-129-5p is increased with α1(I) and CTGF. The authors suggest that since SSc fibroblasts have intrinsic activation of TGF-β, TGF-β suppresses IL-17A, in addition to miR-129-5p with resultant CI accumulation (105).

MicroRNA-29a and miRNA-196a are low in SSc fibroblasts and can suppress CI gene expression, suggesting the low-level expression of the miRNAs permit CI to be upregulated by TGF-β and other mediators in SSc fibrogenesis (97, 99). Levels of other miRNAs have been found to differ in patients with SSc compared to healthy controls as follows: serum miR-142-3p was higher in SSc patients than healthy controls (106); levels of miR-21 were increased, whereas levels of miR-145 and miR-29b were decreased in SSc lesional fibroblasts (94); miR-92a is more elevated in sera and SSc lesional fibroblasts than in normal healthy controls and may downregulate MMP-1 (107); and levels of miRNA-7 were found to be reduced in sera and lesional fibroblasts from patients with localized scleroderma and may regulate CI expression (102). MiR-150 regulates β3 integrin expression and was found to be downregulated in lesional SSc dermal fibroblasts compared to healthy donor fibroblasts (101); miR let-7a was found to be decreased in sera and lesional fibroblasts from patients with SSc or localized scleroderma (104); and miR-21 was found to be upregulated in SSc lesional dermal fibroblasts (93).

Discoidin domain receptor 2 (DDR2) and thrombospondin-2 (TSP2) were both found to be decreased in SSc dermal fibroblasts (103, 104). In SSc dermal fibroblasts, *DDR2* mRNA and protein levels were suppressed, but the knockdown of TGF-β in these fibroblasts resulted in increased expression of *DDR2* (104). In normal fibroblasts, *DDR2* knockdown increased miR-196a expression with resultant decrease in CI. This was not seen when *DDR2* was knocked-down in SSc fibroblasts (104). In SSc, fibroblasts, knocking down *DDR2* did not affect TGF-β signaling or miR-196a expression, suggesting that intrinsic expression of TGF-β causes the downregulation of *DDR2* in SSc fibroblasts (104).

*Thrombospondin 2* mRNA expression and protein levels are decreased in SSc fibroblasts when compared to controls but were upregulated in conditioned medium from SSc fibroblasts (103). Knockdown of *TSP2* in dermal fibroblasts caused decreased expression of CI and increased miR-7 expression (103). SSc dermal fibroblasts show an increased expression of miR-7 (103) suggesting that a negative feedback mechanism may exist between *TSP2* and miR-7 (103).

Matrix metalloproteinase-1 was downregulated when normal dermal fibroblasts were overexpressed with miR-92a (107). In 61 patients with SSc, medium serum levels of miR-92a were elevated. This upregulation was constitutively also found in SSc dermal fibroblast, but when these fibroblasts were transfected with siRNA of TGF-β, the expression of miR-92a was decreased (107). These studies suggest that miR-92a ability to affect *MMP-1* suggest that miR-92a may be a target for *MMP-1*.

### Hair miRNA

MicroRNA from the hair shaft and roots was studied. Hair-miR-196a was found to be significantly decreased in SSc patients (100). Hair miR-29a was obtained from 20 SSc patients, 5 dermatomyositis, and 13 controls to determine its usefulness as a biomarker. Hair miR-29a was significantly lower in SSc patients, and the decreased levels were associated with a higher prevalence of phalangeal contractures (98). We may see more studies using hair miRNAs to assess biomarkers and disease phenotypes.

## Immune System in SSc Pathogenesis

### Role of innate immune system

Engagement of the innate immune system depends on 13 different TLRs, which are not antigen-specific but instead recognize patterns and which segregate on the basis of the nature of the ligands they encounter such as distinct molecular patterns in particular pathogens, in endogenous cellular constituents, or in cellular products of the host [reviewed in Ref. (155)]. Considerable evidence suggests that *TLR2* and *TLR4* expressed on cells and IC *TLR3*, *7*, *8*, and *9* have particular relevance to SSc pathogenesis. For example, a rare functional polymorphism (Pro^631^ His) in TLR2 (which has bacterial peptidoglycan, lipoprotein, and lipoteichoic acid and yeast-derived zymosan as natural ligands) is associated with ATA positivity and enhanced IL-6 production by dendritic cells when engaged by a TLR2 ligand (90, 155). TLR4 endogenous ligands [including fibronectin, hyaluronan fragments, heat-shock protein (HSP) 70, HSP9, high-mobility group box-1 (HMGB-1), and S100A proteins] could engage TLR4 (which is increased in SSc skin and lungs) and synergize with TGF-β to increase fibroblast CI production (155–160). Importantly, HSP70, HMGB-1, and hyaluronan are elevated in SSc sera or tissues (161–163). Of interest, elevated HMGB-1 and soluble advanced glycation end products (sRAGE) levels in sera of patients with SSc correlated with more internal organ involvement, immunological abnormalities, and total MRSS but correlated negatively with lung function (161). Double-stranded RNA is recognized by TLR3, single-stranded RNA, and imidazoquinoline compounds by TLR7 and TLR8, whereas unmethylated CpG oligonucleotide sequences are recognized by TLR9 and some of these ligands are present in SSc (164, 165).

*Siglec-1 (CD169*, *sialoadhesin)* is a marker for macrophage activation and its expression was found to be increased CD14^+^ monocytes in peripheral blood and on macrophages in dermis of lesional skin of a subset of patients with SSc (125). Furthermore, Siglec-1 was induced in peripheral blood CD14^+^ monocytes from normal donors when cultured with IFNα, TLR3, 7, or 9 agonists but not by TLR2 or 4 (125). In the skin, activated macrophages expressing Siglec-1 may also release cytokines or growth factors that are able to stimulate fibroblasts or myofibroblasts to synthesize CIs and other matrix components (125). In addition, sera containing autoantibodies from patients with SSc induce high levels of IFNα in normal monocytes that is inhibited by pretreatment of the sera with bafilomycin and RNA-degrading enzymes, suggesting that the immune complexes in SSc sera contain RNA that can bind IC TLRs (166). While other agents (e.g., IL-4, LSP, IFNβ, IFNγ) might also induce Siglec-1 expression on monocytes/macrophages in SSc, these findings are compatible with the notion that generations of IFNα by activation of IC TLRs 3, 7, or 9 agonists might be ongoing in a subset of SSc patients (125, 167).

### Interferon signature in SSc

Interferons are multifunctional cytokines that are responsible for inducing cellular resistance to viruses. IFN-α, -β, and -ω are type 1-IFNs. There is evidence for a prominent IFN signature in SSc. For example, peripheral whole blood cells in 50% of SSc patients have increased expression of IFN-regulated genes and lung tissues from SSc patients with ILD have increased IFN and IFN-regulated gene expression (168, 169). It appears that the IFN signature in SSc discussed below may arise from activation of TLRs expressed on the surface of cells by infectious agents or by endogenous proteins, RNA, DNA, and other cellular products that can trigger IC TLRs summarized above. IFN regulatory factors (IRF) coordinate the expression of IFN and IFN-inducible genes that help regulate the innate and adaptive immune responses (169, 170). Thus far, *IRF5*, *IRF7*, and *IRF8* appear to be relevant to SSc (169) (see Table 3).

### IRFSNP associations

*IFN regulatory factor 5*, a major regulator of type 1-IFN, induces the transcription of IFN-α and other pro-inflammatory cytokines, is involved in TLR signaling, and is critical for activation of IFN-associated genes (109, 169) (see Table 3). *IRF5* has association with SLE (171–173), and multiple studies have shown SNPs of *IRF5* are associated with SSc susceptibility. *IRF5* rs2004640*TT was found to have a strong association with dcSSc, fibrosing alveolitis, antinuclear antibody (ANA), and ATA positivity in a French cohort (38). In addition to rs2004640, these same authors found an association between rs3757385 and rs10954213 variants and SSc (43). In this study, *IRF5* haplotype “R” was identified as a risk while haplotype “P” was protective (43). A Japanese case–control association study with 281 SSc and 477 controls found that rs2004640, rs10954213, and rs2280714 were all significantly associated with SSc, with rs2280714 having the strongest association with SSc, and these SNPs were significantly enriched in dcSSc and ATA-positive patients (45). Carmona et al. found that SNPs rs10488631, rs2004640, and rs4728142 showed strong associations in SSc global disease, and that association of rs20004640 was dependent on rs4728142 (174). rs728142*A-rs2004640*T haplotype explained this association suggesting that all three haplotypes provide an additive effect (174). In another study, *IRF5* SNP rs4728142 was found to be predictive of longer survival in SSc patients with ILD (41). *IRF7* is upregulated in peripheral blood cells from patients with early SSc and is associated with ACA-positive SSc (175). *IRF8* is induced by IFNγ and modulates TLR signaling (24). Polymorphism rs11642873 in the *IRF8* gene was found to be associated with lcSSc (24). *IRF8* SNP rs2280381 was found to have association with SSc in a Japanese population consisting of 415 SSc and 16,891 controls with a replication study consisting of 315 SSc (68). While associations of the above variations in IRF genes with certain manifestations do not establish cause and effect, they suggest genes that regulate IFN expression and downstream effects may play a central role in determining disease severity and specific organ involvement.

### Inflammasome and SSc

The cytoplasm of cells also contains another pattern recognition receptor (PRR) system called the nucleotide-binding and oligomerization domain (NOD)-like receptor (NLR) family that recognize IC motifs and, when activated via the “inflammasome” involves NFκB and mitogen-activated protein kinase (MAPK), which in turn stimulates production of pro-inflammatory cytokines IL-1B and IL-18. Polymorphisms of one of the NOD family members, *NLRP1*, are associated with ILD and ATA positivity in patients with SSc (176). Relevance of the NOD family to SSc was further evidenced by studies showing inhibition of inflammatory activation-reduced IL-1β and CI production by SSc lesional fibroblasts and studies in *NALP3* null mice showing they were resistant to lung fibrosis (177, 178). *NLRP3* and pro-inflammatory cytokines (IL-1β and IL-18) were found to be increased in skin biopsies of patients with dcSSc or lcSSc compared to age-matched control and correlated with MRSS (179).

### Transitioning from innate to adaptive immunity

Rather than two separate and mutually exclusive immune systems, it is being realized that there is likely an ongoing interplay between the innate and adaptive immune systems (180). Attention has focused on innate lymphoid cells (ILCs) that are involved not only in immediate immune host defense but also in maintaining homeostasis of mucosal and lymphoid tissue (180, 181). Three different types of ILCs have been described to-date: *ILC1*, *ILC2*, and *ILC3* (181). These ILCs do not express somatically rearranged antigen receptors, but express MHC Class-II and possess transcription factors and cytokine profiles reminiscent of Th cells (181, 182). ILC1s, like Th1 cells, utilize T-bet and produce IFNγ; ILC2s, like Th2 cells, utilize GATA-binding protein-3 (GATA-3) and produce IL-5, IL-9, and IL-13; and ILC3s, like Th17 cells, utilize RAR-related orphan nuclear receptor gamma transcription factor (RORγt) and produce IL-17A and IL-22 (181). ILCs express TLRs and IL-1 receptor, and ILC2s and ILC3s can act as APC similar to dendritic cells (181, 183, 184). In mouse models, ILC3s were shown to promote antigen-specific CD4^+^T cells and antigen-specific T-cell-dependent B-cell antibody production (181). What role ILCs play in innate and adaptive immunity in SSc remains to be defined and ongoing research should eventually better elucidate how ILC effect transition from innate to adaptive immunity.

Dendritic cells by using surfaces and IC PRRs play key roles in linking innate immune response to adaptive immune responses by identifying antigens from pathogen-associated or damage-associated molecular patterns (PAMPS or DAMPS) by using TLRs, NLRs, RIG-I-like receptors (RLRs), and receptors for advanced glycation end products (RAGE) (185). The identified antigens are then processed and the information is presented to T cells in the context of MHC-II/antigen complex binding the T-cell receptor, CD86/CD80 costimulation of T-cell CD28, followed by release of cytokines from dendritic cells that affect T-cell differentiation and effect Th1, Th2, Th17, and T regulatory (Treg) cell differentiation (185, 186).

### Adaptive immunity in SSc

A number of observations over several decades strongly implicate a major role for the adaptive immune system in SSc pathogenesis. These include the development of features of SSc in chronic graft-versus-host disease (cGVHD) in humans, which is largely mediated by donor T cells and reversal of fibrosis and vasculopathy after autologous hematopoietic CD34^+^ stem cell treatment of patients with SSc (187, 188).

Immunohistochemical analysis of skin of patients with SSc shows perivascular and tissue accumulations of activated *CD4^+^*T cells**, *monocytes*, and *CD4^+^*CD8*^+^*double positive T cells** that express high levels of IL-4 (189, 190). DNAX accessory molecule-1 (DNAM-1) modulates adhesion; co-stimulates T lymphocytes; expresses on most CD4^+^ and CD8^+^ T cells, NK cells, monocytes, platelets, and some B cells; and is found to be expressed on inflammatory cells in biopsies of lesional skin of patients with SSc (191).

### Autoantigens recognized by SSc T cells

Of particular significance is the finding in lesional SSc skin sites of *Vdelta1^+^*/gamma/delta T cells** that express HLA-DR and CD49d, suggesting that they have homed to these locations and expanded (192). Furthermore, analysis of T-cell repertoire in different skin locations from the same patient is compatible with clonal expansion of T cells to a widely distributed and persistent antigen (193). A variety of autoantigens that elicit T-cell responses in patients with SSc are widely distributed in tissues, have been described, and include types I, II, and V CIs (CI, CII, CV); laminin; low molecular weight (MW) *N*-sulfated heparin sulfate; 3500 MW RNA antigen; elastin; and DNA topoisomerase I (189, 194–198). Of potential relevance is the finding that the CI-specific CD25^+^CD4^+^ T cells isolated from SSc PBMC have a memory (CD45R^+^) phenotype (195). Most patients with SSc have production of IFNγ by their PBMCs when cultured with CI or constituent α1 and α2 chains, which can be reduced by inducing immune tolerance via chronic administration in a dose-dependent manner by oral bovine CI (199, 200). In a double-blind, randomized clinical trial of daily oral bovine CI or placebo for 12 months, patients with dcSSc ≥3 years duration, patients receiving oral bovine CI had a significant improvement in MRSS compared to the placebo-treated patients (201). These studies suggest CI might be a widely distributed relevant antigen in SSc.

### Microchimerism in SSc

Fetal–maternal and maternal–fetal microchimerisms have been proposed as mechanisms triggering autoimmunity in SSc and other autoimmune diseases (202–204). This microchimerism, in susceptible individuals, could initiate a type of cGVHD producing SSc with the microchimeric cells acting as effectors or as targets of an immune response (204). It is noteworthy that, in women with SSc who have given birth to male children, male offspring Th2-oriented T cells that express high levels of IL-4 are found in these women’s skin and blood (205).

### CD4^+^ regulatory T cells and CD4^+^ Th17 T cells in SSc

The dysregulation in SSc of Th17 and/or Tregs (mostly CD4^+^CD25^+^Foxp3^+^) has been reported by several groups. Different (and contradictory) results have been reported that seem to be dependent to some extent on how Tregs are defined by flow cytometry. Tregs have been found to be increased in the blood of SSc patients but have defective suppressive function (206). Papp et al. (207) reported decreased percentages and suppressive function of CD4^+^CD25^+^Tregs but increased percentage of Th17 cells in blood of SSc patients (207). Klein et al. (208) reported SSc patients had elevated CD4^+^D24^+^Foxp3^+^Tregs in lesional skin but normal percentages in the peripheral blood (208). Slobodin et al. reported an increased number of Tregs in the blood of SSc patients but no concomitant increase in TGF-β or IL-10 production by CD4^+^T cells (209). Fenoglio et al. found SSc patients had reduced frequency in blood and reduced suppressive function of CD4^+^CD25^+^Tregs and increased Th17 cell expansion after polyclonal or antigen-specific stimulation of SSc PBMC (210). Finally, Mathian et al. analyzed circulating activated (a)Tregs (CD4^+^CD45RA^(^CD25^bright^ T cells) and resting (r)Tregs (CD4^+^CD45RA^+^CD25^+^ T cells) in controls and SSc and found decreased frequency but normal suppressive function of both types of Tregs and in the lesional skin found no CD4^+^Foxp3 mRNA in SSc compared to normal donor skin (211).

Abnormalities in Treg numbers or function could facilitate development of adaptive immune responses to autoantigens in SSc. Mast cells and S1P which are increased in SSc are two potential antagonists for proper development and function of Treg cells, as both have the capacity to inhibit Tregs (212–214). Furthermore, both S1P and mast cells enhance generation of Th17 cells (213, 215). The field of Tregs is still evolving and future studies with better markers for Treg subsets will need to be performed to better characterize this role in SSc.

### Possible influence of vitamin D deficiency and lysophospholipids on immune dysregulation in SSc

Vitamin D insufficiency/deficiency has been implicated in triggering and enhancing a number of autoimmune diseases. Low serum 25(OH)D concentrations have been reported to be more common in patients with SSc than in healthy controls. Furthermore, 25(OH)D levels have been reported to negatively correlate with several laboratory and clinical parameters in European Disease Activity Score, Raynaud’s phenomenon (RP), erythrocyte sedimentation rate, systolic pulmonary artery pressure, MRSS, and positively correlate with carbon monoxide diffusion lung capacity (216–218). A number of effects of 1,25(OH)_2_D3 on immune cells have been reported that could explain its ability to decrease autoimmunity and, conversely, how VitD deficiency contributes to increased autoimmunity [these are summarized in Ref. (219)]. For example, effects of 1,25(OH)_2_D3 on APC include: (1) downregulation of MHC class-II molecule expression in APC; (2) downregulation of surface expression of co-stimulatory receptors (CD40, CD80, and CD86) and other maturation-induced proteins (CD1a, CD83); (3) inhibition of dendritic cell maturation, induction of tolerogenic DC that are able to induce Treg cells; (4) inhibition of IL-12 p70 release from DC; and (5) inhibition of pro-inflammatory cytokines in monocytes and macrophages (219). Effects of 1,25(OH)_2_D3 on T cells include: (1) inhibition of antigen-specific and lectin-stimulated T-cell activation and progression from G1a to G1b proliferation; (2) inhibition of IL-12, IFNγ, IL-2 release; (3) stimulation of IL-4, IL-5, and IL-10 production; and (4) inhibition of Fas ligand (FasL) expression by activated T cells (219). The effect of 1,25(OH)_2_D3 on B cells is to inhibit production of IgA, IgE, IgG, and IgM and in NK cells to inhibit IFNγ production (219, 220).

Administration of VitD3 in escalating daily doses of 2000 U (2000 U for the first month, then 4000 U for the second month, and 8000 U for the third month) to healthy VitD-deficient individuals induced increased frequencies of CD38^+^ B cells and reduced frequencies of CD4^+^IFNγ^+^ and CD4^+^IL-17^+^ T-helper cells (221). Treatment of SLE patients with hypovitaminosis D with 100,000 U of VitD3 weekly for 4 weeks and then monthly for 6 months resulted in an increase in naïve CD4^+^ T cells and CD3^+^CD4^+^CD25^hi^CD127^–^Foxp3^+^Tregs and decreases in CD19^+^ B cells, anti-ds DNA antibody titers, and proteinuria (222). Similar studies with high-dose VitD supplementation have not been reported in patients with SSc, but the above studies in SLE and normal hypovitaminosis individuals demonstrate the potential for immune modulation by high-dose VitD supplementation that might decrease autoimmunity in patients with SSc.

*Lysophosphatidic acid* and *S1P* levels are increased in sera of patients with SSc, suggesting they may play a role in different aspects of the disease (214) [reviewed in Ref. (223)]. Platelets, macrophages, dendritic cells, mast cells, and endothelial cells are sources of LPA and S1P, and these cells (plus T cells and B cells), NK cells, fibroblasts, and other cells express various types of LPA and S1P G-protein-coupled receptors (GPCRs) [reviewed in Ref. (223)]. *PPAR*γ**, which resides intracellularly and counters TGF-β fibrogenesis, is also an additional receptor for LPA (224). In addition to S1P being able to “disarm” Foxp3 Tregs mentioned above, S1P and LPA regulate the function, migration, and trafficking of all lymphoid cells and monocyte/macrophage/dendritic cells with S1P also being able to sequester T cells in the thymus and peripheral lymphoid organs, resulting in some instances in lymphopenia, which is frequently found in patients with SSc (225–227). By acting on APC, S1P and LPA each can suppress development of Th1 T-helper cells, but they have different effects on Th2 T-helper cells in that S1P suppresses their development while LPA fosters their development (228). Th2 T-helper cell predominance is a feature of some patients with SSc with production of IL-4 and IL-13, which facilitate development and expansion of B cells and autoantibodies that are common features of SSc. Lysophospholipids need further study in SSc, given the potential to regulate immunity.

## Vascular Abnormalities in SSc

Vascular dysfunctions and abnormalities leading to RP, digital ulcers, and nail-fold capillary abnormalities usually are among the earliest and key manifestations of SSc. The various vascular abnormalities are summarized in Table 5. Postmortem examination reveals the vascular changes in SSc are more typical of a vasculopathy than of a vasculitic process – given the paucity of inflammation in the vessel wall with widespread systemic intimal proliferation in the pulmonary, coronary, and the renal arteries (229). Patients with SSc who develop PAH and renal crisis exhibit vascular lesions characterized by classic concentric intimal proliferation, marked luminal obstruction, lymphocyte infiltration, and relative paucity of plexiform lesions (230–233).

**Table 5:** **Key vascular abnormalities of SSc**.

Presence of proliferative vasculopathy with intimal proliferation in peripheral, pulmonary, coronary, and renal arteries in the absence of inflammation is a hallmark feature of scleroderma
Endothelial cell damage is a key and early process. It precedes fibrosis and particularly involves the arterioles
Early detectable changes in the endothelial cells include disappearance of membrane-bound vesicles, vacuolization of endothelial cell cytoplasm, and gaps between endothelial cells
Defective angiogenesis is an early event in the form of drop out of capillaries and abnormal capillary architecture without a compensatory process
There are conflicting reports regarding the presence and role of circulating endothelial progenitor cells in SSc
There is dysregulation of coagulation and fibrinolysis process
Platelets show enhanced aggregability to various triggers such as type I collagen and adenosine etc…, and are activated throughout the clinical course of SSc
LPA and S1P could potentially contribute to the vasculopathy via endothelial cell activation, neointimal formation, vascular leakiness, increased vasoconstriction, cardiac fibrosis, and hypertension

Earliest signs of vascular dysfunction include impaired vascular tone and vascular permeability (234). Impaired balance of vasoconstrictor substances (e.g., ET) and vasodilator substances (e.g., NO), plays important roles in vascular dysfunction. Platelet activation and enhanced coagulation with reduced fibrinolysis also contribute to the vasculopathy in SSc. Abnormalities in the vascular system can be seen in clinically normal skin of SSc patients (235). Large gaps between endothelial cells, vacuolization of endothelial cell cytoplasm, and loss of membrane-bound storage vesicles are some of the earliest detectable changes in the endothelial cells (235–237). In a 20-year follow-up study, sequential changes can be seen in capillaries (4) in skin, which include capillary enlargement, capillary loss, and telangiectasia. Further morphologic changes in vessel wall occur including fibrosis. Such capillary changes are wide spread in internal organs (e.g., lungs, heart, kidneys, and muscles) (238). Intimal proliferation and accumulation of proteoglycans in the arterioles and small arteries are also common (239, 240). The operative mechanisms that lead to this widespread vasculopathy in SSc of unknown, but animal models and *in vitro* studies have provided some clues.

### Mechanism of vascular and endothelial cell injury in SSc

The etiology of the initial vascular damage in SSc is not known and is a topic of speculation. Infectious agents, cytotoxic T cells, NO-related free radicals, and autoantibodies against endothelial cells have all been implicated (234). Endothelial cell dysfunction, neural abnormalities, and various other intravascular defects likely contribute to the impaired vascular flow (241).

#### Endothelial Cell Injury

Evidence suggests that endothelial cell injury is an early and central event in the pathogenesis of SSc vasculopathy, and viral agents [especially human cytomegalovirus (hCMV)], cytotoxic T cells, antibody-dependent cellular cytotoxicity (ADCC), anti-endothelial cell antibodies, and ischemia-reperfusion injury are all suggested mechanisms for endothelial cell damage (234, 242). Levels of antibodies to hCMV are increased in patients with SSc which is reminiscent of the association of hCMV antibodies with vascular intimal proliferation and vasculopathy in patients with graft rejection and coronary artery bypass restenosis (243). In addition, there is evidence of binding of some ATAs to an epitope in hCMV-derived UL94 protein which happens to also show homology to MVEC surface protein tetraspan novel antigen-2 (NAG-2) (243). Apoptosis of MVEC can be effected by purified anti-UL94 peptide antibodies (244). Cytotoxic CD4^+^ T cells induce MVEC apoptosis via *in vitro* Fas-related pathway in contrast to CD8^+^ T cells, NK, and LAK cells which utilize the granzyme/perforin system (243). ADCC to MVEC is operative in many patients with SSc (243). Anti-endothelial cell antibodies are commonly found in sera from patients with SSc and are capable of inducing MVEC apoptosis directly *in vitro* (245). Ischemia and reperfusion injury (especially associated with attacks of RP) is accompanied by upregulation of expression of junctional adhesion molecules (JAMs). This upregulation indicates endothelial dysfunction and allows attachment of platelets and neutrophils to the endothelium that is thought to lead to MVEC injury through production of superoxide radicals (which limit release of vasodilation substances such as NO and prostacyclin) (243, 246, 247). The major evidence for the presence of the endothelial injury in SSc is high serum levels of circulating von Willebrand (VW) factor, ET-1, increased levels of circulating viable and dead endothelial cells, and soluble JAM-A and JAM-C (234, 247–251). Subendothelial tissue forms a nidus for platelets to aggregate and initiates fibrin deposition and intravascular thrombus formation (1). The role of endothelial apoptosis is not clear. Sgonc et al. (252) demonstrated endothelial cell apoptosis in the University of California at Davis chicken lines 200/206, which spontaneously develop an SSc-like disease (252). Apoptotic endothelial cells may contribute to tissue injury when engulfed by immature dendritic cells and macrophages, which subsequently present cellular antigens to CD8^+^ T cells, causing further tissue injury (253). These apoptotic endothelial cells can also activate the alternate complement pathway and coagulant pathway leading to vasculopathy (254, 255). Proof that there is ongoing endothelial apoptosis in SSc is thus far lacking, and Fleming and Wanless (256) failed to detect apoptotic endothelial cells in their study, although they did demonstrate loss of VE-cadherin, which regulates endothelial barrier function and found evidence of IFNα signaling (256). IFNα signaling suggests endoplasmic reticulum stress and the unfolded protein response in these cells (257, 258).

#### Defective Angiogenesis

The remarkable loss of capillaries and small vessels in patients with SSc suggests a defect in the process of angiogenesis. Tissue ischemia usually leads to the expression of angiogenic growth factors [e.g., vascular endothelial growth factor (VEGF)], which causes vasodilatation, proliferation, and migration of endothelial cells and stabilization of the lumina to form new vessels (259). Plasma levels of VEGF are elevated in SSc, and this could stimulate angiogenesis (260). Levels of other proangiogenic factors [e.g., PDGF, placental growth factor (PGF), and fibroblast growth factor 2 (FGF-2)] are also considerably elevated in the plasma of SSc patients (261). Expression of VEGF and its receptors, *VEGFR1* and *VEGFR2*, are increased in skin of SSc patients (260, 262, 263). In addition to elevated level of VEGF, other proangiogenic mediators (such as ET-1, adhesion molecules, and chemokines) are found in the circulation of SSc patients (264). Elevated levels of antiangiogenic factors such as *angiostatin*, *platelet factor-4* (also called CXCL4), thrombospondin-1 (*TSP-1*), and *IL-4* have been described in patients with SSc (264, 265).

#### Defective Vasculogenesis

The role of vasculogenesis in SSc is not clear, and there are conflicting reports regarding the presence and role of circulating endothelial progenitor cells in SSc (266). Increased levels of circulating endothelial progenitor cells have been demonstrated which supports their mobilization from bone marrow (267). However, in another study, there were substantially reduced numbers of bone marrow-derived circulating endothelial precursors compared to healthy subjects or patients with RA. The lowest number of these cells was observed in SSc patients with active fingertip ulcers, and this may suggest inadequate recruitment of these precursor cells and impaired vascular repair mechanisms (268). Atorvastatin can be effective in RP – perhaps by increasing the number of circulating endothelial progenitor cells, which suggests a role of endothelial progenitor cells in vascular dysfunction (269). Apoptosis of endothelial progenitor cells by a circulating factor has been implicated as the potential mechanism for the reduced number of circulating precursor cells in SSc (270). Mesenchymal stem cells might be another source of endothelial progenitor cells. In SSc, the angiogenic potential of these cells is reduced (271). This suggests that endothelial repair may be affected by unknown SSc disease effects on the bone marrow.

*Pericytes* mediate vascular maturation and stabilization during angiogenesis (272). They can further differentiate into vascular smooth muscle cells, fibroblasts, and myofibroblasts (273–275). Pericytes express *PDGFR-*β**, and high molecular weight melanoma-associated antigen (HMW-MAA) in vascular lesions in SSc patients with associated RP and ANA (276). Another marker of angiogenic pericytes is regulator of G protein signaling (RGS-5), which is highly expressed in SSc vasculature (277). The exact role of RGS-5 is not clear, but it can negatively regulate vessel maturation (278). Pericytes proliferate and contribute to increased vascular wall thickness, which is characteristic of SSc vasculopathy (279).

#### Endothelial to Mesenchymal Cell Transition in the Pathogenesis of SSc Vasculopathy

There is subendothelial accumulation of activated fibroblasts or myofibroblasts and production of excessive CI and ECM components in blood vessels of SSc patients (1). During this process, endothelial cells lose their specific markers such as *VE-cadherin* and *VW factor* and acquire a mesenchymal phenotype expressing α smooth muscle actin (αSMA), Vimentin, and CI. It is postulated that endothelial cells might transform into mesenchymal cells induced by local growth factors and cytokines (1). The exact molecular mechanism and the cytokines involved are not known, but TGF-β has been implicated. There are recent reports of TGF-β being involved in various disease processes such as endothelial to mesenchymal transformation (280–284). Li and Jimenez (285) further examined the role of TGF-β in the transformation process and the signaling pathways involved (285) in a murine pulmonary endothelial cell model. They concluded that TGF-β could lead to mesenchymal transformation of the endothelial cells. They further demonstrated that the transformation is associated with strong upregulation of transcriptional repressor snail-1 and is mediated by the c-abl kinase and protein kinase C-δ. Snail-1 is a zinc-finger transcription factor that forms a complex with Smad3/Smad4 (1). Snail-1 induces numerous transcriptional events that could lead to expression of a mesenchymal phenotype. Besides this, Wnt signaling as well as NOTCH signaling pathways might be involved in this endothelial–mesenchymal transformation process (1). Other potential mediators of this transformative process include PDGF (286), VEGF (287), insulin-derived growth factor (288), CTGF (289), ET-1 (290), and miRNAs (291, 292). Endothelial to mesenchymal cell transition is an interesting concept but needs further study to determine what role, if any, it plays in SSc vasculopathy.

#### Circulating Mediators of Vasculopathy

Higher levels of *ET-1* have been observed in patients with scleroderma renal crisis, lung fibrosis, PAH, and RP (293). Increased ET-1 expression is associated with increased ET-1B receptor in the skin and lung tissue of SSc patients (294).

In SSc, there is a reduction in *eNOS* gene expression and *NO release* in SSc and MVEC derived from lesional and non-lesional skin biopsies in the steady-state and after shear stress (295). This is probably associated with deficient endothelium-dependent relaxation in SSc (296). Impaired NO results in alteration of vascular tone, enhancement of platelet aggregation, and increased susceptibility of endothelial cells to oxidative injury. NO also limits cytokine-induced endothelial cell activation and monocyte adhesion and inhibits the endothelial cell release of IL-6 and IL-8, which are important inflammatory cytokines (297). Further, NO inhibits vascular smooth muscle cell proliferation through elevation of cyclic GMP and inhibition of mitogenic proteins, TGF-β and PDGF. Therefore, impaired NO production in SSc may contribute to the pathogenesis of arteriolar intimal proliferation and may have a prominent role in pathophysiology of the disease.

#### Coagulopathy in Systemic Sclerosis

*Coagulation and fibrinolysis* processes are dysregulated as evidenced by presence of microvascular thrombosis and enhanced fibrin deposition frequently seen in the vasculature of SSc patients. The loss of balance between fibrinolysis and coagulation contributes to vessel engulfment with fibrin and breakdown of vessel patency (298). The authors demonstrated impairment of fibrinolysis and activation of the coagulation pathway in a study of 29 patients (298). Activation of the coagulation system, as well as elevated levels of fibrinogen and VW factor, has been demonstrated in patients with SSc (299–302). Reduction of fibrinolysis, expressed as defective tissue t plasminogen activator (tPA) antigen release and/or elevated tPA inhibitor (PAI) antigen, supports existence of heterogeneous hypofibrinolytic pattern in SSc (303).

*Plasmin* has both pro-fibrotic and anti-fibrotic properties [pro-fibrotic by activating TGF-β and anti-fibrotic by activating both hepatocyte growth factor (HGF) and MMPs] (304, 305). Plasmin is inactivated via formation of a complex with α2-antiplasmin (α2AP), and elevated levels of plasmin-α2AP are associated with several fibrotic conditions including SSc (306). α2AP promotes fibrosis by activating phospholipase A_2_ by binding to adipose triglyceride lipase (ATGL) to generate PGF_2α_, which in turn stimulates production of TGF-β (307). Levels of α2AP are elevated in lesional BLM skin in mice, which is induced by CTGF via extracellular signal-regulated kinase 1/2 (ERK 1/2) and JNK pathways (308). α2AP induces αSMA^+^ myofibroblasts *in vitro* and mice with deletion of α zinc-finger alpha protein gene (αZAP) exhibit less infiltration of myofibroblasts at the site of BLM injections in the skin (308). Plasmin increases ECM degradation, and inhibition of plasmin of α2AP decreases ECM degradation, which could be another mechanism by which α2AP could promote fibrosis.

#### Platelet Abnormalities in SSc

Chronic activation of platelets and their released products could contribute to the vascular, immunologic, and connective tissue pathology of SSc (309). SSc platelets show *enhanced aggregation* to various triggers [e.g., CI, adenosine diphosphates, 5-hydroxytryptamine (309–311), ET-1, S1P, and LPA (223)]. ET-1 and S1P cause vasoconstriction by engaging S1P_2_ and S1P_3_ receptors (312). In the human fetal lung fibroblast line (FH-1), S1P utilizes S1P_1_ receptors to inhibit TGF-β1-induced αSMA expression while utilizing S1P_3_ receptors to stimulate αSMA expression (313). Sera from patients with SSc have elevated levels of arachidonoyl-LPA and S1P (214). LPA induces platelet aggregation, vascular smooth muscle proliferation, and neointima formation, which can induce vasospasm and RP (314–317).

The various platelet-derived factors include: *inflammatory mediators* [NO, serotonin, thromboxane A_2_, prostaglandin (PG)D_2_, PGE_2_, PGF_2_, 12-hydroxyeicosatetraeonic acid, β thromboglobulin, neutrophil-activating peptide-2, platelet factor-4, platelet activating factor, adenosine, histamine, P-selectin, CD40 ligand (CD40L), dinucleoside polyphosphates, 2-arachidonyl glyceride, MMP-27], *chemokines* [macrophage inflammatory protein (MIP-1α); monocyte chemoattractant protein-3 (MCP-3); IL-8; and regulated upon activation, normal T-cell expressed and secreted (RANTES)], *cytokines* [IL-1β and granulocyte monocyte-colony stimulating factor (GMCSF)], and *growth factors* [(PDGF) A, B, C, D, TGF-β1 and 2, epidermal growth factor, VEGF-A and C, brain-derived neurotrophic factor, insulin-like growth factor-1 (IGF-1), basic fibroblasts growth factor (bFGF), HGF, and CTGF] (309). Platelets from scleroderma patients overexpress a specific non-integrin *65-kDa receptor for CI*, phosphatidylinositol (PI)-3 secondary to increased nitrotyrosylation and increased protein kinase B (Akt) activity (309, 318). Overexpression of these mediators is induced by cytokines produced by T cells and monocytes activated by autoantigen such as CI that (in turn) changes the phenotype of megakaryocytes (318). The platelets store numerous *fibrogenic mediators* and contribute to chronic tissue fibrosis in SSc by release into tissue of TGF-β1, TGF-β2, PDGF-A, B, C, D, LPA, S1P, adenosine, bFGF, CTGF, and IGF-1. These aforementioned mediators have many biological properties and effects on a host of cells that could also facilitate and contribute to autoimmunity and fibrosis (5).

#### Animal Models Resembling SSc Vasculopathy

Animal studies in mice recapitulate some of the vasculopathy of SSc. Mice with a conditional deletion of *Fli1* develop systemic vascular lesions characterized by capillary dilation, vascular fragility, stenosis of arterioles, increased vascular permeability, micro-aneurysms, decreased expression of platelet/endothelial cell adhesion molecule (PECAM)-1, PDGF-β, and S1P type I receptor (S1P_1_) and increased endothelial cell MMP-9 expression (319).

*Caveolin-1 (cav-1)* is one of three membrane proteins that coat caveolae which are plasma membrane invaginations important in clustering together of receptors that can influence signal transmission of the specific receptor ligand (320). *Cav-1* is involved in internalization and degradation of TGF-β receptors, thereby reducing signaling by TGF-β (321, 322). There is decreased expression of *cav-1* in lesional skin and lungs of patients with SSc and in lungs of patients with idiopathic pulmonary fibrosis (IPF) (323, 324). *Cav-1* null mice develop PAH and right and left ventricular enlargement and failure (325). However, in contrast to *cav-1* null mice with PAH, in human IPAH, there is an apparent increase in cav-1 expression in the PASMC compared to healthy controls and that the over expression of *cav-1* increases capacitive Ca^++^ entry and DNA synthesis in PASMC (326). The cav-1 null mice also develop pulmonary fibrosis, raising questions regarding the etiology of the PAH in this model which is yet to be clearly defined. In a French and Italian SSc population, *Cav-1* rs959173C showed protective association with SSc and lcSSc (327). The rs959173C protective allele is associated with increased CAV-1 protein expression (327).

*Fos-related antigen-2 (Fra-2) transgenic (TG)* mice develop microvascular and proliferative vasculopathy and express *Fra-2* in vascular structures (endothelial cells and vascular smooth muscle cells) similar to its expression in skin of SSc patients (328). An early event in the *Fra-2* TG model is apoptosis of endothelial cells (328). The *Fra-2* TG mice also developed pulmonary vascular lesions resembling SSc-associated PAH and later developed dermal and pulmonary fibrosis resembling the “non-specific interstitial pneumonia” (NSIP) (328). These results suggest *Fra-2* might be involved in pathogenesis of SSc vasculopathy and to-date this is the only mouse model that manifests both vasculopathy and fibrosis with features shared by the human SSc disease.

## Pulmonary Arterial Hypertension

### Cellular stress in SSc-PAH

Patients with lcSSc, who also have PAH, have the highest expression of the endoplasmic reticulum stress/unfolded protein response genes, *Activating Transcription Factor-4al-b*, a spliced form of X-box BP, and immunoglobulin-heavy-chain BP (257). In PBMC of the lcSSc patients, HSP gene (*DNAJB1*), and IFN-regulated genes (*IFIT1*, *IFIT2*, and *IFITM1*) were upregulated, but *IRF4* was downregulated compared to healthy controls (257). Further analysis showed that the severity of PAH (as reflected in pulmonary artery pressure) positively correlated with level of *DNAJB1* expression, while endoplasmic reticulum stress marker correlated with IL-6 levels in the whole lcSSc population (257).

### Interferon signature in SSc-PAH

Type I IFNs are implicated by the association of use of IFNα in the treatment of hepatitis and of IFNβ in the treatment of MS with development of PAH (118, 119). Diseases in which there is an “IFN signature” (such as SLE, SSc, and infection with HIV) are associated with development of PAH (120–124). Furthermore, IFNα and IFNγ (added to cultures of human PASMC primed with TNFα or to cultures of human lung MVEC or human lung fibroblasts) cause release of the potent vasoconstrictor, *ET-1*, and of *IP-10* (117). In a series of 128 SSc patients with PAH and 35 patients with no PAH, the SSc patients with PAH had higher levels of IP-10 and ET-1 in their sera compared to SSc patients without PAH or compared to healthy controls; more SSc patients with PAH had detectable levels of IFNα and IFNγ in their sera than SSc patients without PAH (117). In this series of SSc patients, levels of TNFα, IL-12p70, IL-6, IL-1α, and IL-8 were significantly higher in sera in SSc patients with PAH when compared to SSc patients without PAH (117). Additional studies of this patient group revealed that serum levels of IP-10 in the SSc-PAH patients correlated with pulmonary vascular resistance, and levels of brain natriuretic peptide in serum, and serum IP-10 levels in the SSc-PAH patients inversely correlated with cardiac index and 6-min walks test (117). Sections of lung from patients with IPAH or with SSc-PAH expressed higher levels of IFNR1 in endothelium, smooth muscle layer, vascular interstitium, and in intravascular inflammatory cells as assessed by immunohistochemistry and Western blotting (117). While the above studies strongly implicated type I IFN as playing a pathogenic role in SSc-PAH and IPAH, further evidence was substantiated in the *type I IFN* α *receptor 1* knockout mouse which was found to be resistant to experimental hypoxic PAH induction. These mice did not have elevated serum levels of ET-1 when compared to WT control mice (117). Analysis of PBMC from patients with SSc revealed *Siglec-1* and other IFN-regulated genes were overexpressed in patients with dcSSc, whereas patients with lcSSc with PAH overexpressed *IL-13RA1*, *ICAM-1*, *CCR1*, *JAK2*, and *MCR1* (123, 125, 126). IL-13 was also elevated to higher levels in sera of patients with lcSSc with PAH, and *MCR1* was induced on CD14^+^ monocytes suggesting monocytes are activated in lcSSc patients with PAH of an alternative (i.e., IL-4/IL-13) rather than classical (i.e., IFNγ/LPS) pathway (123).

### Other mediators and gene polymorphisms in SSc-PAH

Polymorphisms were described in the promoter of the *NOS2* gene that confers susceptibility to PAH in SSc (144).

In another report, patients with lcSSc with PAH, had higher levels of circulating monocyte-related cytokine mediators (TNFα, IL-1β, IL-6, and ICAM-1) and vascular injury markers (VEGF, VCAM-1, and VW Factor), and their PBMCs exhibited increased expression of mRNA for ICAM-1, IL-1β, JAK2, IFNGR1, IL-13Rα1, tissue inhibitor of metalloproteinase (TIMP)-2, delta-aminolevulinate synthase 2 protein (ALAS2), CCR1, and AIF1akt (126).

*Urokinase-type plasminogen activator receptor*, *CD87*: (discussed under “Genetics of SSc”) SNP, *UPAR* rs344781G allele, is associated with SSc-related digital ulcers, pulmonary artery hypertension, ACA positivity, and lcSSc (91).

*Sphingosine 1-phosphate* and *LPA* may have effects on the vasculature in SSc that contribute to some of the abnormalities observed in the disease. For example, there is overexpression of VE-cadherin, IFNα signaling, and *Rgs-5*, which is associated with an antiangiogenic phenotype (188). Overexpression of *Rgs-5* may reduce signaling via S1P_1_ receptor and increase S1P signaling through other S1P receptors that could reduce endothelial eNOS, increase vasoconstriction, increase vascular leakiness, and reduce angiogenesis [reviewed in Ref. (223)]. Furthermore, S1P may contribute to PAH by constricting pulmonary arteries while LPA may contribute to systemic hypertension, cardiac fibrosis, endothelial cell activation, and neointima formation (via PPARγ) [reviewed in Ref. (223)].

Lysophosphatidic acid, S1P, and other chemoattractants (such as TGF-β1, TGF-β2, IL-8, MCP-3, and other mediators released from aggregated/activated platelets adhering to damaged microvascular endothelium and diffusing into perivascular tissue) could establish chemotactic gradients that would promote outward transversal migration of monocytes, dendritic cells T and B lymphocytes, and NK cells resulting in perivascular accumulation of these cells to set the stage permitting innate and adaptive immune responses that lead to autoimmunity and fibrosis (223).

## Fibrosis in SSc

### Links to the innate and adaptive immune systems

Over three decades ago, it was recognized that human lymphocytes and monocytes (when stimulated by antigen or T-cell mitogen *in vitro*) elaborate soluble mediators (lymphokines, monokines, growth factors, chemokines, and cytokines) that induced fibroblast chemotaxis or (when added to cultures of human fibroblasts) induce fibroblast growth and synthesis of collagenase (MMP-1) and CI (329–340). These studies provided tangible evidence that immune cells are fully capable of modulating chemotaxis and growth of fibroblasts, as well as regulating synthesis of CI and CIII and the major CI degradative enzyme, MMP-1, by fibroblasts.

Later studies conducted with purified recombinant or natural cytokines, chemokines, and growth factors known to be synthesized by cells of the innate and adaptive immune system have allowed fibroblasts specific functions to be assigned to certain ones. *TGF-*β*1*, which is produced by most cell types but also by CD4^+^CD25^+^Foxp3 Tregs, monocytes/macrophages, mast cells, and platelets and *IL-4*, which is produced by Th2 cells, and mast cells received early attention as being potent stimulators of CI synthesis and chemotaxis by fibroblasts (341–345).

Cells of the innate and adaptive immune system elaborate a variety of cytokines and chemokines in addition to TGF-β and IL-4 (such as *IL-6*, *PDGF*, *IL-1*, *IL-13*, *IL-17*, *IL-5*, *MCP-1*, and *CTGF*) that have been found to be increased in serum or in tissues in which excess connective tissue matrix is accumulating in SSc. These cytokines/chemokines are at the interface between the immune system and fibroblasts.

*Signal transducer and activator of transcription protein 4* is critical for T-cell signaling and differentiation (132–134). *STAT4* is involved in effecting a Th1 cytokine response by transmitting signals from IL-2, IL-12, and IL-23 receptors and in signaling after type 1 IFN engages its receptor (135, 136). The role of *STAT4* in fibrosis was assessed in scleroderma mouse models. The deletion of *STAT4* significantly reduced skin fibrosis in the BLM model but not in the Tsk-1/+ model (137). In the BLM model, it was noted that there were decreased numbers of inflammatory cells including T cells and proliferating T cells and decreased quantity of IL-6, IL-2, TNFα, and IFNγ in lesional skin of *STAT4^–/–^* vs. *STAT4*^+/+^ mice (137). In addition to having a role in SLE and RA susceptibility, *STAT4* has been identified as a susceptibility gene in SSc (50) (see Table 3).

*Macrophage migration inhibitory factor-173* acts upstream and activates innate immunity. It plays a role in sustaining cellular and inflammatory response. It causes fibroblasts proliferation and acts as an antiapoptotic (135).

### Links to vascular damage

*Endothelin-1* is one of three isoforms and is synthesized by VE cells, fibroblasts, bone marrow mast cells, neutrophils, macrophages, and cardiac myocytes (140) (See discussion under “Genetics of SSc”). ET-1 is overexpressed in skin biopsies of patients with dcSSc (179).

*Fos-related antigen-2*, reviewed above, appears to have both vasculopathic and fibrogenic properties and may be a contributor to these processes in patients with SSc.

### SSc fibroblast phenotype and myofibroblasts

Earlier studies indicated that normal human dermal fibroblasts (grown for prolonged periods of time *in vitro* in the presence of culture medium supplemented with culture supernatants obtained by activating normal human donor peripheral blood lymphocytes and monocytes with T-cell mitogen *in vitro*) acquired a “scleroderma-like phenotype” that resembled cultured lesional SSc skin fibroblasts at the ultrastructure level with respect to excessive production of glycosaminoglycans (346). A phenotypic characteristic of cultured SSc lesional skin fibroblasts is that they produce reduced levels of *MMP-1*, an enzyme necessary for degradation of triple helical CI and CIII (347). Some SSc lesional fibroblasts regain production of MMP-1 after several subpassages, and when these fibroblasts lines are then cultured for 3 weeks with *IL-13* or *PDGF-BB*, then cultured in plain medium before TNFα stimulation, the production of MMP-1 in response to TNF-α stimulation is markedly reduced compared to normal donor fibroblasts similarly treated with IL-13 or PDGF-BB (348). These studies suggest that *in vivo* chronic exposure of SSc fibroblasts to certain cytokines, derived from activated lymphocytes and monocytes either in circulation or from lymphocytes/monocytes infiltrating SSc lesional skin, can induce an SSc fibroblast phenotype that persists in the absence of the cytokines for some period of time. Platelets that are being chronically activated/aggregated in patients with SSc may also contribute some cytokines/growth factors (e.g., PDGF-BB) that could contribute to induction of the scleroderma fibroblast phenotype (309).

Fibroblasts cultured from lesional skin biopsies of patients with SSc contain increased numbers of myofibroblasts and synthesize increased amounts of CI and *TIMP-1*, in contrast to fibroblasts grown from non-lesional SSc skin or skin of healthy controls (349). This increased CI production phenotype reverts toward normal as the SSc lesional fibroblasts in culture are passaged, as shown by LeRoy (350). The myofibroblasts in SSc lesional skin contain αSMA and fibronectin ED-A splice variant, the latter being a requirement for TGF-β1 to induce myofibroblast formation (275, 351). In normal wound healing, myofibroblasts contract the newly formed ECM, and their development and function are modulated by mechanical forces and stiffness of the ECM microenvironment (352). The origin of myofibroblasts in SSc lesional skin is not completely understood, but likely candidates include resident connective tissue fibroblasts, epithelial cells, pericytes, and circulating fibrocytes. Myofibroblasts are induced by a number of cytokines, growth factors, and other agents present in SSc tissue or serum, including: *TGF-*β*1*, *TGF-*β*3*, *IL-4*, *TNF*α**, *IL-6*, *GMCSF*, *thrombin*, *bradykinin*, *histamine*, *tryptase*, *oncostatin M*, *IL-13*, *PDGF-*β**, *ET-1*, *TLR 2/1 ligands*, and the *lysophospholipids*, *S1P* and *LPA* (5, 353–355).

Levels of *IL-1*α** are elevated in sera of patients with SSc, and SSc monocytes produces more. IL-1 than normal monocytes when stimulated *in vitro* (356, 357). IL-1α and -β stimulate proliferation of human dermal fibroblasts and upregulate production of CI, TIMP, PGE_2_, MMP-1, and hyaluronan (358, 359). IL-1α and -β were observed to promote viability of cultured SSc lesional skin fibroblasts and myofibroblasts *in vitro* in the presence or absence of serum and directly induced expression of αSMA and N-cadherin (360). This suggests that IL-1 may contribute to the longevity of myofibroblasts in SSc skin.

Fibroblasts grown from SSc lesional skin biopsies constitutively overexpress IC IL-1α; and after stimulation *in vitro* with TNFα or IL-1β, both *icIL-1*α** and *icIL-1* receptor protein antagonist (*icIL-1ra*) are markedly upregulated compared to normal donor fibroblasts (361). Overexpression of icIL-1α in normal skin fibroblasts also induces expression of icIL-1ra (361). When icIL-1ra is overexpressed in cultures in normal human skin fibroblasts via transfection with a viral vector (pLXSNicIL-1ra type 1), it induces a myofibroblast phenotype characterized by increased expression of **α*SMA* and *PAI-1* (362).

Treatment of SSc lesional fibroblasts with IL-1α siRNA resulted in decreased proliferation and production of IL-6 and CI, whereas stably transfecting with icIL-1α induced proliferation and IL-6 and CI synthesis (363).

### TGF-β receptor-Smad signaling in fibroblasts

A great deal of effort has elucidated the complex receptor engagement and signaling of TGF-β and its 1, 2, and 3 isotypes that occur in mammals and which have been the topic of several recent reviews (364–367). TGF-β1, 2, and 3 are synthesized as *inactive propeptides* which have to be cleaved intracellularly by the protease, *farin*, to generate active 25 kDa MW, active TGF-β1, 2, or 3. The active TGF-β is bound by the cleaved amino terminal peptide called “latency-associated peptide” (LAP) and, in connective tissue, the latent TGF-β1-LAP complex is bound to latent TGF-β1-binding protein (LTBP), which is termed “large latent complex” (LLC) (365, 368). Latent TGF-β can be activated by interaction with *integrins* and by *several proteases* such as thrombin, plasma transglutaminase, cathepsin D, and plasmin (369). There are three classes of TGF-β receptors. *TGF-*β* receptor 1* has two forms: *ALK1* (found mainly in endothelial cells) and *ALK5* (which is present in most cells) (367). *TGF-*β* receptor 2* forms a heteromeric complex with type 1 receptors and phosphorylates it, setting in motion IC signaling via receptor-regulated Smads (R-Smads) which are type 1 receptor specific [i.e., ALK1 causes Smad1/5/8 phosphorylation while the predominant ALK5 causes Smad2/3 phosphorylation (367)]. The phosphorylated R-Smads complex with Smad4 and in the nucleus interact with co-activators [e.g., CREB-binding protein (CBP)/p300] and co-expressors (e.g., Ski/Sno) to transcriptionally activate or repress target genes (367). Inhibitory Smads (Smad 6 and 7) can bind to TGF-β type 1 receptors and to Smad4 or effect ubiquitination and proteasomal degradation (367). A coreceptor called *endoglin*, of which there are two spliced variants called short and long forms, can (under different conditions by interacting with ALK1 or ALK5) decrease or enhance TGF-β signaling, respectively (367). *Betaglyan* (“type 3” TGF-β receptor) can also act as a coreceptor by facilitating TGF-β binding/interaction with type 1 and 2 TG-β receptors (370). *CTGF* can also interact with TGF-β type 1 and 2 receptors and facilitate Smad3 signaling, which has a pro-fibrotic effect (367). Other members of the TGF-β superfamily including *Activin* (*A*, *B*, and *AB*), bone morphogenic proteins (BMPs), and growth differentiation factors utilize components of the TGF-β receptor complex (366). In addition to the canonical Smad-dependent pathway described above, TGF-β can signal through non-canonical Smad-independent Wnt, MAPK, phosphatidylinositol-3-kinase/AKT, and Rho-like GTPase pathways (366). Activating transcription factor 3 (ATF3), which regulates oxidation and cellular stress, is upregulated in SSc dermal fibroblasts by TGF-β; and ATF3 suppresses TGFG-β-induced proliferative effects via interaction with Smad3 in a c-Jun-dependent manner (371).

Recently, it was reported that the fibrogenic effect of IL-6 in fibroblasts is brought about by binding of IL-6 to soluble IL-6 receptor (IL-6R) by a JAK1 and STAT3-dependent mechanism that is mediated through *Gremlin-1*, which utilizes TGF-β type 1 and 2 receptors and the TGF-β signaling pathway dependent on Smad3 that leads to CI gene expression, but is not dependent on TGF-β protein (372).

Transforming growth factor-β induces the early response gene (*Egr-1*), via a Smad-independent pathway via MEK1/2/ERK signaling (373). Overexpression of *Egr-1* induces CI gene upregulation (374). In addition, IL-13 and insulin-like growth factor-binding protein-5 (IGF-BP-5) have been shown to induce *Egr-1* expression by MAPK signaling pathway (375). Other extracellular signals which are relevant to SSc [such as PDGF, hypoxia, HGF, or LPS (bacterial LPS), oxidative stress, thrombin, LPA, ultraviolet light, cigarette smoke, mechanical strength, ischemia-reperfusion, and T-cell receptor ligature] have been shown to increase *Egr-1* expression (373). TGF-β also induces *Egr-3* by canonical Smad3 signaling, and *Erg-3* overexpression stimulates CI gene expression (376).

### Antifibrotic mediators

*Bone morphogenic protein-7*, although a member of the TGF-β superfamily, stimulates fibroblast chemotaxis like TGF-β1, but does not induce CI, fibronectin, hyaluronan, or TIMP synthesis (377). BMP-7 also inhibits fibrogenic properties of TGF-β1 (378) and signals through a receptor complex structurally different from that of TGF-β and utilizes SMAD1/5/8 (365). *IL-10* inhibits both proliferation and CI synthesis by fibroblasts (379). Certain IL-10 genotypes have been associated with development of SSc in Caucasian and Japanese subjects (380). *TNF*α** inhibits CI, stimulates MMP-1 synthesis by fibroblasts, and is a potent chemoattractant for these cells (381). *IFN*γ** is a potent inhibitor of expression of CI and CIII mRNA and protein by cultured SSc fibroblasts *in vitro* (382). To what extent BMP-7, TNFα, IL-10, IFNγ, or other antifibrotic mediators or mechanisms try to counter the drivers of fibrosis such as TGF-β, IL-4/IL-13, IL-6/IL-6R-Gremlin-1 in SSc is unknown but provides candidates to be the focus of future studies.

Effect of blocking *TNF*α** with etanercept was assessed in the BLM scleroderma mouse model. Compared to vehicle-treated mice, the etanercept-treated mice had less dermal fibrosis and lower serum levels of TGF-β1 than controls not treated with etanercept (383). Etanercept has not been efficacious in ameliorating dermal fibrosis in patients with SSc (384) (see Table 3).

*Peroxisome proliferation-activated receptor gamma-*γ**, when engaged by ligands of different types, blocks TGF-β-mediated fibrotic responses *in vitro* in cultured fibroblasts and in various fibrotic animal models *in vivo* (81, 82). PPARG rs310746 is associated with SSc (83).

In the *cGVHD* murine model of scleroderma induced by transferring splenocytes from B10.D2 donor mice into BALB/c recipients, tolerizing the recipient BALB/c mice *by oral administration of protein extract of BALB/c spleens* for 11 days after transfer of B10.D2 splenocytes was associated by upregulation of IL-10 and downregulation of IFNγ production by T cells from the BALB/c recipients and protected the recipient BALB/c mice from dermal fibrosis and other manifestations of cGVHD (385). IL-10 was likely produced by Tregs induced by oral tolerance induction by the BALB/c spleen extract and was likely responsible for suppression development of fibrosis (379).

### Genome-wide gene expression of skin

The fibrogenic role of *TGF-*β**, *IL-13/IL-4*, and *Egr-1* in patients with SSc has been assessed by performing genome-wide gene expression studies on lesional and non-lesional skin biopsies from patients with dcSSc, lcSSc, morphea, and healthy controls. These studies show four intrinsic subsets of gene expression termed “diffuse proliferation” (further divided into diffuse1 and diffuse2) and containing only dcSSc patients; inflammatory group containing dcSSc, lcSSc, and morphea; limited group containing lcSSc; and a normal-like group containing normal, dcSSc, and lcSSc patients (386). Further comparisons were made subjecting TGF-β, IL-13/IL-4, and Egr-1-stimulated normal dermal fibroblasts in culture to gene expression microarray analysis and comparing these fibroblast microarrays to gene expression arrays of biopsies of skin from SSc, morphea, and normal donors. TGF-β responsive gene signature was found in 10 out of 17 patients with dcSSc (59%) and none of 7 lcSSc, none of 3 morphea, and none of 6 healthy controls (387). The dcSSc patients with the TGF-β-responsive signature had higher MRSS and likelihood of having ILD (387). The TGF-β signature-positive dcSSc patients also were in the diffuse-proliferation subset; however, one in the diffuse-proliferation subset did not have the TGF-β signature. This suggests that only a subset (and not all) SSc patients have the TGF-β signature. The fibroblast *Egr-1*-responsive gene signature was present in the skin biopsies from diffuse-proliferation subset of dcSSc patients, but was not present in biopsies of patients with lcSSc, morphea, or healthy controls (388). The *IL-4* response signature overlapped approximately 60% with the IL-13 response signature, which were both enriched in the SSc inflammatory subset (389). Expression in skin biopsies from SSc patients of the IL-13 pathway activation [as well as transcripts of IL-13 receptor components (*IL-13RA1* and *IL-4RA*)] correlated with MRSS (389). Expression of *CCL2* (MCP-1) transcripts also correlated with MRSS and *IL-13RA1* (389). This study also assessed gene expression profiling in skin of a sclerodermatous graft-versus-host disease (scl GVHD) model in *Rag2^–/–^* mice, which were found to also exhibit the IL-13 pathway activation resembling that in SSc patients of inflammatory subset (389). This observation is interesting, given that it has been hypothesized that fetal–maternal or maternal–fetal microchimerism might induce a cGVHD state in some patients with SSc as described above. Since IL-6 and IL-6R induces Gremlin-1 protein (which then signals through the canonical Smad-dependent pathway), it raises the question as to whether some of the TGF-β signature in the dcSSc diffuse-proliferation subset (discussed above) is actually due to *Gremlin-1*. Further studies would need to be done comparing Gremlin-1-induced gene signature in dermal fibroblasts with that of TGF-β1 to sort this out.

A more extensive genome-wide expression profiling skin biopsies involving analysis of additional pathway-specific gene signature for *PDGF*, *S1P*, *PPAR-*γ**, *TNF*α**, *IFN*α**, *NF*κ*B*, *IL-13*, *IL-4*, *poly (I-C)*, and *inomycin-phorbol 12-myristate 13-acetate (inomycin-PMA)* was recently conducted by this group (390). Results showed IFNα signaling was strongly associated with early disease, compatible with the notion that innate immune response may be a feature in early disease which was contrasted with TGF-β signaling being a feature of later disease with worse MRSS (390). Surprisingly, PDGF signaling was most strongly associated with the fibroproliferative subset (more so than TGF-β), and the inflammatory subset exhibited strong activation of innate immune pathways including enrichment of IL-4, S1P, NFκB, LPS, poly(I-C), and TGF-β gene signatures (390). The findings support an earlier hypothesis by Gabrielli et al. that a stepwise process of SSc development begins with inflammatory (e.g., IFNα signaling) and continues with fibrosis (e.g., PDGF and TGF-β signaling) and ends in atrophy (391). IL-4 pathway was significantly enriched in the inflammatory subset more than IL-13, and suggests a T_H_2 enhancement of immune response in patients within the inflammatory subset (390).

Most patients with dcSSc have some resolution with the passage of time of dermal fibrosis after the onset of their disease. This has been observed in several different studies clinically as decreases in the MRSS. In a large, single SSc center in the UK, 131 patients with dcSSc had MRSS measured repeatedly up to 36 months after onset of their disease (392). Three patterns were discernable as follows: those with high baseline MRSS that did not improve over 36 months from baseline (38%); those with high baseline MRSS that improved over 36 months from baseline (21%); and those with low baseline MRSS that improved over 36 months from baseline (35%). The reason for these three clinical trajectories of change in MRSS over time is not apparent, but could be a function of different genetic backgrounds, different triggers, or other environmental modifications that either ameliorate or contribute to perpetuation of the disease. The patients received different medications; however, clinical MRSS response or survival could not be attributed to any of the medications (392). The fact that most of these patients with dcSSc had improvement in their MRSS suggests that the myofibroblast phenotype responsible for excessive ECM deposition does not persist, that the fibrotic skin can revert toward normal, and that a normal-like homeostasis can be re-established in such patients. This study suggests that those dcSSc patients with persistently high MRSS likely have a continuous presence of a driver of dermal fibrosis that constantly stimulates the fibroblasts to maintain the myofibroblasts phenotype with maintenance of increased ECM in their dermis. Application of the genome-wide gene expression studies of skin biopsies, in a cohort such as this one in which patients have skin biopsied repeatedly over several years, may shed light on the mechanisms responsible for the three different MRSS trajectories over time, and would answer the question whether the inflammatory subset morphs into the fibroproliferative subset.

### Vitamin D and fibrosis

Vitamin D has a variety of antifibrotic actions. Studies *in vitro* have demonstrated *1,25(OH)_2_*D3** inhibits growth of murine fibroblasts (393–397), inhibits fibroblast-mediated contraction of CI gels (largely a TGF-β-stimulated function) (398), inhibits fibroblast synthesis of IL-6 and IL-8 (399, 400), and inhibits production of plasminogen activator (401). It was also observed that 1,25(OH)_2_D3 *in vitro* inhibited CI and CIII synthesis by fibroblasts grown from different human tissues including bone marrow, lung, and skin (402, 403). In mice, *in vivo* administration of 1,25(OH)_2_D3 has been shown to ameliorate renal interstitial fibrosis, glomerulosclerosis in rats, and reduce conversion of adipose tissue to fibrous tissue in mouse skin exposed to chronic UV irradiation (404, 405).

The cutaneous formation and metabolism of VitD in patients with SSc has been reported to be normal (406–408). In one report, fibroblasts grown from biopsies of lesional skin from patients with SSc and from healthy volunteers were inhibited in proliferation and CI synthesis to a similar extent by 1,25(OH)_2_D3 addition to the fibroblast culture (409). The VitD receptor (VDR) in SSc lesional skin fibroblasts is reported to be decreased, likely due to TGF-β’s ability to downregulate the VDR (410).

Studies using a mouse mesenchymal multipotent cell line revealed that 1,25(OH)_2_D3 promoted increased expression and nuclear translocation of the VDR; decreased expression of TGF-β1 and plasminogen activator inhibitor (SERPINE 1); decreased expression of CI I, III, and other CI isoforms; and increased expression of several other antifibrotic factors including *BMP-7*, *MMP-8*, and *follistatin* [an inhibitor of the pro-fibrotic factor, myostatin (411)]. Studies in rat interstitial myofibroblasts showed that 1,25(OH)_2_D3 inhibited in a dose-dependent manner (10^–9^–10^–6^ M) TGF-β1-induced *de novo* αSMA expression and suppressed CI and TSP-1 expression induced by TGF-β1, which was shown to be mediated by upregulated HGF (412).

Slominski et al. have discovered the skin and other tissues in humans synthesize other VitD derivatives [including *20(OH)D3*, *20,23(OH)_2_*D3**, and *17,20(OH)_2_*pD**] that, also like 1,20(OH)_2_D3, *in vitro* inhibit CI and hyaluronan synthesis by fibroblasts grown from normal or SSc lesion skin (144). Unlike VitD3, 25(OH)D3, or 1,25(OH)_2_D3, these novel endogenously produced VitD analogs are non-calcemic when given in high doses to mice. 20(OH)D3 also suppressed development of dermal fibrosis in the BLM, scleroderma mouse model (413). These results suggest multiple endogenous forms of VitD3 have antifibrotic properties that may prove useful in SSc as therapeutic agents in the future.

### Lysophospholipids and fibrosis

*Lysophosphatidic acid* induces fibroblast chemotaxis and proliferation (414). LPA induces αvβ6 integrin-mediated TGF-β activation by engaging LPA_2_ receptors on epithelial cells and makes fibroblasts resistant to apoptosis, which is a characteristic of SSc lesional fibroblasts that would prolong their survival (415, 416). Evidence that LPA is involved in myofibroblast formation in SSc lesional skin was suggested by the finding that fibroblasts cultured from skin of SSc patients exhibited increased LPA-activated chloride current, which is a hallmark of LPA-induced myofibroblasts (417). *AMO95*: a selective small molecule inhibitor of LPA_1_ signaling (AMO95) protected mice from developing BLM-induced skin fibrosis and increased regression of established BLM-induced skin fibrosis (418). Contrary to the results in the BLM skin fibrosis model in which LPA_2_ knockout did not affect dermal fibrosis, in the BLM lung fibrosis model, LPA_2_ knockout mice exhibited reduced lung injury, fibrosis, and fibronectin deposition in BLM-treated lungs (419). *S1P* facilitates migration of fibroblasts in response to a chemotactic gradient of fibronectin in a S1P_2_ receptor-dependent manner (420). S1P signals through the Smad pathway utilized by TGF-β1 in fibroblasts and other cell types and mimics TGF-β1 pro-fibrotic effects in that it decreases MMP-1 and increases TIMP and CI production by fibroblasts (421–423). SSc dermal fibroblasts express more S1P_3_ receptors than control donor fibroblasts and exhibit an exaggerated pro-fibrotic response to TGF-β1 (421). Furthermore, S1P levels are elevated in sera of patients with SSc (214). As mentioned above, S1P gene signature is prominent in the inflammatory subset (390). Fingolimod (*FTY720*) has both agonist and antagonist effects in different S1P receptors and modulates lymphocyte trafficking, monocyte/macrophage biology, dendritic cell biology, and enhances Treg function at marginal zone B lymphocytes (144). When administered to chronic scleroderma graft-versus-host disease (cScl-GVHD) mice, FTY720 in either preventative or therapeutic protocols reduced fibrosis, expanded splenic myeloid suppressor cells, increased Tregs and B regulatory cells (Bregs), protected against vascular damage, reduced serum S1P and E-selectin levels, reduced numbers of inflammatory cells in skin, and reduced dermal expression of mRNA for TGF-β1, MCP-1, MIP-1α, RANTES, TNFα, IFNγ, IL-6, IL-10, and IL-17A (424). FTY720 also returned phosphatase and tensin homolog (PTEN) and Smad3 phosphorylation to normal levels in cScl-GVHD mice (424). Although FTY720 is approved to treat MS, its use in SSc clinical trials has not been reported.

## The Endocannabinoid System and SSc

The ECS is an endogenous regulatory network made up of multiple GPCRs and a series of endogenous arachidonic acid derivatives, which act in an autocrine fashion and seem to play a homeostatic role affecting diverse key biologic and physiologic processes including angiogenesis, cell proliferation, apoptosis, differentiation, metabolism, immune function, and vascular tone that may have implications for SSc pathogenesis and potential therapeutic targets. The term “endocannabinoid” generally refers to the first two characterized endocannabinoids (ECs), anandamide (AEA) and 2-arachidonoyl glycerol (2-AG), though a number of endogenous cannabinoid receptor agonists have since been discovered. AEA and 2-AG may be degraded into free arachidonic acid by fatty acid amide hydrolase (FAAH) and monoacylglycerol lipase (MAGL), respectively, but may also be metabolized by lipoxygenases, cyclooxygenase 2 (COX-2), and P450 epoxygenases, and acyl transferases yielding a vast library of EC analogs with different actions and target receptors (425). Some of these metabolites engage EC receptors, while others have been demonstrated to modulate the activity of ECs via “entourage effects” at cannabinoid receptors 1 (CB1) and 2 (CB2), inhibition or potentiation of ECS degradation, activation of downstream targets such as *PPAR*γ** or metabolic interconversion (426).

Efforts to identify new cannabinoid receptors are ongoing; but CB1, CB2, and GPR55 are among the most extensively studied, and recent studies clearly demonstrate that all three of these receptors are activated by AEA and play a key role in transduction of EC signaling. Interestingly, CB1, CB2, and GPR55 have also been shown to modulate one another’s activity via heteromerization, cross-antagonism, and other strategies (427, 428). GPR18 (also known as “abnormal cannabinoid receptor”), another candidate cannabinoid receptor, may play a role in EC-mediated central blood pressure control and peripheral vascular tone (429, 430). Unlike CB1, CB2, and GPR55; however, activation of this receptor requires FAAH-mediated metabolism of AEA or 2-AG to *N*-arachidonoyl-glycine (431). It is interesting to speculate that if there is reduced FAAH in tissue-expressing GPR18 in patients with SSc as has been found in SSc dermis, then this might contribute to vasoconstriction and hypertension (432). Substantial cross talk has also been established between the ECS and a network of non-selective action channels known as the transient receptor potential vanilloid (TRPV) family, which serve to integrate mechanical and environmental stimuli with local autocrine signals to effect a variety of cell processes (433, 434).

### Endocannabinoid system as a therapeutic target in SSc

The ECS is an appealing potential target for treatment of SSc, as it modulates endothelial cell function, vascular tone (including pulmonary artery vasodilation), the innate immune response to injury, autoimmunity, and fibrogenesis (435–440).

#### Endocannabinoid Modulation of the Immune System

The general effect of cannabinoids on cells of the immune system is to act as immunosuppressive and anti-inflammatory agents. Although immune cells express more CB2 than CB1, both receptors and other non-CB receptors (such as PPARγ and GPR55) have been implicated in effecting immunomodulatory actions of cannabinoids (441, 442). CB1 mRNA and protein expression in/on immune cells is responsive to cellular activation signals, i.e., cell type, cannabinoid ligand type, and immune stimulus-dependent (443). Of relevance to SSc, IL-4 is specifically able via STAT5 pathway to induce CB1 mRNA in human T cells (444). Dendritic cells exposed to cannabinoids undergo NFκB-dependent apoptosis and reduce production of IL-12, which is important in priming Th0 cells to a Th1 orientation (445, 446). Cannabinoids induce apoptosis of T cells via CB1 and CB2 engagement and effect a Th2 polarization (e.g., increased IL-4 by T cells) while decreasing Th1 polarization (e.g., decreased IL-12 by DC) but also suppress activation, differentiation, and expansion of T cells (443, 447, 448). B cells are affected by cannabinoids in several ways, including direct effects on activation, differentiation, and proliferation but also via effects on T cells that provide help to B cells (449). The effect of cannabinoids on B cells to suppress IgM and enhance IgE production apparently is mostly via engagement of CB2 (450). Production of pro-inflammatory cytokines (including TNFα, IFNγ, IL-2, and IL-1β) is suppressed *in vitro* and *in vivo*, by engaging CB2 by cannabinoids or other CB2 agonists (451–453). Of particular relevance to SSc is that mast cell activation is inhibited by 2-AG (454). The CB2 agonist, Gp1a, was found to suppress clinical disease in the EAE mouse model with a reduction in Th1 and Th17 cells in peripheral lymphoid organs. Analysis of the CD4^+^ cells *in vivo* in the periphery revealed Gp1a-treated mice had lower levels of expression of T but also RORγt (Th17 marker) and exhibited increased Foxp3 and GATA-3 expression (455). Under polarizing conditions *in vitro*, Gp1a suppressed Th1 and Th17 development of CD4^+^ T cells (455). The role of the ECS in innate and adaptive immune dysregulation in SSc is an area for further investigation, and these results with this CD2 agonist suggest similar agents might decrease autoimmunity and autoantibody production in SSc.

#### Endocannabinoid Modulation of the Vasculature

The upregulation of *ICAM-1* and *VCAM-1* on the endothelium of human coronary arteries by treatment with TNFα or LPS is inhibited by the CB2 agonist drug, *JWH-133* (437). Engaging CB2 in rat coronary arteries by AEA induced the coronary arteries to dilate (456). Blocking CB1 on isolated human coronary smooth muscle cells by the CB1 antagonist, *rimonabant*, reduced ability of the smooth muscle cells to migrate and proliferate in response to PDGF (438). The ECs (*AEA* and *virodhamine*) were found to have a potent vasodilatory effect on preconstricted isolated human pulmonary artery rings that was endothelium-dependent and likely involved PGE_2_ (436). The effect of AEA and virodhamine was CB1- and CB2-independent but involved a third receptor termed “endothelial cannabinoid receptor” (436). These studies suggest that a target for development of treatment for PAH in SSc might be based on virodhamine-like drugs that are agonist for the endothelial cannabinoid receptor.

#### Endocannabinoid Modulation of Fibrosis

C57BL/6 mice with either *TRPV1* receptor or *calcitonin G-related peptide (CGRP)* knocked out compared to WT mice developed enhanced dermal fibrosis after repeated subcutaneous injection of BLM (434). This suggests that *TRPV1* receptor and *CGRP* have antifibrotic effects and may have relevance to patients with RP and SSc since skin biopsies from patients with SSc have reduced numbers of *CGRP*-immunoreactive C fibers and would likely have reduced vasodilatation from *CGRP* in response to stressors that trigger RP (434). It is unclear whether other *TRPV1* receptor engagement or *CGRP* effects are operative to protect against fibrosis. *TRPV4* has been implicated as a mechanosensor in endothelial cells and fibroblasts, and has been shown to stimulate myofibroblast differentiation in rat cardiac fibroblasts via integration of mechanical and soluble (autocrine) signals, and pretreatment with the TRPV4 antagonist, *AB159908*, resulted in significant inhibition of TGF-β1-induced myofibroblasts differentiation of cardiac fibroblasts (457). *5,6-EET* (generated by activation of *PAR-2* by mast cell tryptase or Factor Xa) has been implicated as the most likely autocrine mediator contributing to activation of *TRPV4*, though other autocrine signals may be involved as well, with the known *TRPV4* agonist *N*-acyl taurine being another possible candidate. It is worth noting that this system of channels seems to be dysregulated in dcSSc fibroblasts with profound downregulation of *TRPV2* (and possibly *TRPV1*) and overexpression of *TRPV4*. While the role of *TRPV1* and *TRPV2* is less clear, *TRPV4* is known to stimulate myofibroblast differentiation in response to activation by mechanical stress and arachidonic acid derivatives. In normal wound repair, release of the myofibroblast from this mechanical stress signal plays a role in inducing apoptosis or, alternatively, may help drive the myofibroblast back into a quiescent fibroblast. Given that TRPV4 remains overexpressed in the dcSSc fibroblast, this may suggest that certain autocrine signals are present, which alter the cellular milieu in favor of constitutive activation of *TRPV4*, thus rendering the myofibroblast incapable of responding appropriately to mechanostress signaling. AEA and 2-AG activation of *TRPV4* is indirect and requires hydrolysis of these compounds to free arachidonic acid, which is then converted into the potent *TRPV4* agonist 5,6-EET. The fatty acid amide *N*-acyl-taurine was recently discovered to be a potent agonist of TRPV4, as well, and is likely overexpressed in SSc owing to underexpression of FAAH (432). This compound was shown to be elevated 10-fold following experimental inactivation of FAAH, with highest levels noted in the lungs and kidneys (458). Furthermore, inaction of FAAH in mice increases dermal fibrosis in response to subcutaneous administration of BLM (432).

Serine proteases activate PARs, which have been associated with fibrosis of internal organs (459–461). *PAR-1* is expressed by keratinocytes, endothelial cells, and fibroblasts, while *PAR-2* is expressed in suprabasal keratinocytes in SSc lesional skin and in healthy donor skin (462). There is more expression of PAR-1 by fibroblasts in biopsies of SSc lesional skin than by fibroblasts in biopsies of normal donor skin, and PAR-2 was expressed only by SSc lesional skin fibroblasts and not by normal donor fibroblasts (462). A large portion of fibroblasts in samples from SSc lesional skin were myofibroblasts, staining positive for αSMA suggesting that PAR-1 and PAR-2 may be involved in fibrosis development in SSc (462). PAR-1 is increased on SSc-associated ILD myofibroblasts, and when it is inhibited by the direct thrombin inhibitor, dabigatran, there is abrogation of formation of myofibroblasts, αSMA, and production of CI (463). Agonists of PAR-2 include mast cell tryptase and Factor Xa, and mast cells have been demonstrated to stimulate human lung fibroblast proliferation via activation of PAR-2.

It is worth noting the importance of COX-2 in the metabolism of ECs – specifically regarding 2-AG, which is more readily metabolized by COX-2 than AEA and has also been shown to activate PPARγ via a mechanism that is COX-2 dependent. Recent studies suggest the COX-2 metabolite of 2-AG, 15-deoxy-PGJ2-G, activates PPARγ (464) and that inhibition of IL-2 secretion by AEA in murine splenocytes is attenuated by COX-2 inhibition and also partially antagonized by PPARγ inhibition (465). Further work should be directed at evaluating PPARγ as a downstream target of the oxygenated metabolites of AEA and 2-AG, as this nuclear receptor is known to modulate fibrogenesis (likely by being a transcriptional repressor of TGF-β), autoimmunity, and a wide range of other physiologic processes (82, 466). 2-AG is oxygenated by COX-2 and other PG synthases to produce several different glycerol-esters of the prostaglandins (PG-Gs). During the early stages of inflammation, in which microsomal prostaglandin E2 synthase (mPGES)-1 is high, one would expect to see higher levels of PGE_2_-G. Similarly, during resolution, in which PGD_2_ and its spontaneous degradation products predominate, one would expect to see higher levels of PGD_2_-G and its metabolites. Interestingly, the EC analog of 2-AG, which is known as 15-deoxy-PGJ_2_-G, also has been shown to activate PPARγ and this cyclopentanone-EC derivative may be the mediator of 2-AG’s activation of PPARγ. Further characterization of the P450-derived epoxides of AEA and 2-AG is needed, especially given the importance of P450, epoxygenase in PAR-2-mediated sustained activation of TRPV4, which may play a role in perpetuating fibroblast activation.

The efficacy of several cannabinoid agonists in attenuation of fibrosis has been demonstrated in both the BLM and hypochlorite murine models of SSc. Additionally, these compounds have been shown to counter several behavioral abnormalities of SSc fibroblasts, including reversal of myofibroblast differentiation and decreased resistance of SSc myofibroblasts to apoptosis, with the ultimate effect of decreased ECM deposition and attenuation of fibrogenesis. The exact mechanism by which these cannabinoid agonists exert their antifibrotic action is still a matter of debate, but it seems to be mediated in part by activation of CB2 and PPARγ. CB1 and CB2 are both overexpressed in dcSSc fibroblasts. Twenty-four hour incubation with the CB1/CB2 agonist, *WIN55,212-2*, resulted in agonist-induced inhibition of both CB receptors, which was reversible after agonist withdrawal (439). After 10 μM WIN55,212-2 incubation, a reduction in CI mRNA and protein was observed in both dcSSc and healthy fibroblasts (439). TGF-β and CTGF mRNA expression, as well as IL-6 levels were also substantially decreased after exposure to WIN55,212-2 (439). Analysis for αSMA by Western blotting, RT-PCR, and immunocytochemistry showed that WIN55,212-2 induced reduction in αSMA expression by 43% and increased by twofold the number of apoptotic fibroblasts from patients with dcSSc but not in fibroblasts from healthy donors (439). Pre-incubation of dcSSc and healthy fibroblasts with synthetic cannabinoid receptor antagonists *AM281* (CB1 antagonist) and *AM630* (CB2 antagonist) did not significantly reverse the effects of WIN55,212-2 on CI neosynthesis, inhibition of IL-6, or fibroblast apoptosis, indicating that the antifibrotic actions of WIN55,212-2 are mediated, in part, by pathways not involving CB1 and CB2, perhaps as the authors suggest by transducing pathways involving p-ERK (439). Incubation of dcSSc fibroblasts and healthy controls with WIN55,212-2 was noted to result in decreased phospho-ERK-1/2 protein expression in both groups (439). WIN55,212-2 was found to prevent BLM-induced dermal fibrosis in DBA/2J mice *in vivo*. Levels of phospho-Smad2/3 were analyzed and found to be significantly lower after WIN55,212-2 exposure. Subcutaneous inflammatory cell infiltration, dermal thickness, and CI content were comparable to the control group (440). BALB/c mice injected daily for 6 weeks with PBS or hypochlorite were injected intraperitoneally with PBS or with WIN55,212-2, an agonist of CB1 and CB2, or with JWH-133, a selective agonist of CB2. Both WIN55,212-2 and JWH-133 prevented development of skin and lung fibrosis, as well as fibroblast proliferation and formation of autoantibodies (467).

As mentioned above, FAAH levels are markedly reduced in biopsies of SSc lesional skin compared to skin from healthy donors, and mRNA for FAAH expression in cultured lesional skin fibroblasts from patients with SSc was also reduced from that expressed by cultured normal donor dermal fibroblasts (432). The induction of BLM skin fibrosis in *FAAH* null mice or in normal mice treated with the FAAH inhibitor, *JNJ1661010*, resulted in a marked increase in skin fibrosis at the BLM injection site (432). Furthermore, blocking CB1 receptor in BLM-treated mice with FAAH blocked by JNJ1661010 resulted in a marked increase in skin fibrosis at the BLM injection site (432). Additionally, blocking CB1 receptor in BLM-treated mice with FAAH blocked by JNJ1661010 prevented the enhanced fibrosis induced by BLM treatment, whereas blocking CB2 further enhanced skin fibrosis in BLM-treated mice with FAAH blocked by JNJ1661010, suggesting CB1 mediated fibrosis whereas CB2 dampened fibrosis as a result of increased EC present because of blocking FAAH (432).

The role of the CB1 receptors in the BLM skin fibrosis model and Tsk-1/+ mice model was assessed using *CB1*KO mice (468). WT and *CB1*KO mice were treated with BLM and with the CB1 selective agonist *N*(a-chloroethyl)-5Z, 8Z, 11Z, 14Z eicosatetraenamide (ACEA) (468). *CB1*KO mice were protected from developing BLM dermal fibrosis; however, crossing *CB1*KO mice with Tsk-1/+ mice did not prevent fibrosis (468). This suggested that fibrosis associated with inflammation was dependent on CB1 expression or leukocytes. Indeed, chimeric bone marrow studies revealed that CB1 on leukocytes was essential for leukocyte infiltration and fibrosis in the BLM skin fibrosis model induced by the CB1 agonist (468).

These preclinical studies of CB receptor agonists/antagonist provide useful information for translation of these or similar CB receptor active agents for treatment of SSc.

## Conclusion

Systemic sclerosis is one of the most complex systemic autoimmune diseases that target the vasculature and connective tissue-producing cells (namely fibroblasts/myofibroblasts) and components of the innate and adaptive immune systems – all three of which themselves interact and affect each other. The disease is heterogeneous in its clinical presentation that likely reflects different genetic background or triggering factor influences on the vasculature, connective tissue cells, and immune system. The roles played by other ubiquitous molecular entities (such as lysophospholipids, ECs, and their diverse receptors) in influencing the vasculature, immune system, and connective tissue cells are just beginning to be realized and studied and may offer new therapeutic approaches to treat SSc.

## Author Contributions

*Statement pertaining to each author’s contribution*. *D*rs. DP and AP wrote the “Introduction,” Dr. MB wrote the section on “Genetics and GWAS,” Dr. AP wrote the sections on “Immune System in SSc Pathogenesis,” Dr. DP wrote the section on “Vascular Abnormalities in SSc,” Dr. AP wrote the section on “Fibrosis in SSc” and “Animal Models of SSc/Scleroderma.” Dr. BP wrote the section on “The ECS and SSc.” All authors reviewed the final manuscript.

## Conflict of Interest Statement

The authors declare that the research was conducted in the absence of any commercial or financial relationships that could be construed as a potential conflict of interest.

## Abbreviations

2-AG, 2-arachidonoyl glycerol; α2AP, α2-antiplasmin; AA, African American; ACA, anticentromere antibodies; Acea, eicosatetraenamide; ACR, American College of Rheumatology; ADCC, antibody-dependent cellular cytotoxicity; AEA, anandamide; AFA, anti-fibrillarin antibody; AIF1, allograft inflammatory factor-1; AKT, protein kinase b; ALAS2, delta-aminolevulinate synthase 2 protein; ALK, activin receptor-like kinase; ANA, antinuclear antibodies; APC, antigen-presenting cells; ARA, anti-U3 ribonucleoprotein; αSMA, α smooth muscle cell actin; ATA, anti-DNA topoisomerase I antibody; ATGL, adipose triglyceride lipase; aTregs, activated Tregs; αZAP, α zinc-finger alpha protein gene; BANK1, B-cell scaffold protein with ankyrin repeats 1; bFGF, basic fibroblasts growth factor; BiP, immunoglobulin-heavy-chain binding protein; BLM, bleomycin; BMP, bone morphogenic protein; BP, binding protein; Bregs, B regulatory cells; Cav-1, caveolin-1; CB1/CB2, cannabinoid receptors 1 and CB2 ditto; CBP, CREB-binding protein; CCR1, C–C chemokine receptor type 1 protein or gene; CD40L, CD40 ligand; CGRP, calcitonin G-related peptide; CGS, candidate gene study; cGVHD, chronic graft-versus-host disease; CI, type I collagen; CII, type II collagen; CIII, type III collagen; COX-2, cyclooxygenase 2 (i.e., prostaglandin synthase 2); CpG, C phosphate G; cScl-GVHD, chronic scleroderma graft-versus-host disease; CSK, C-src tyrosine kinase; CTGF, connective tissue growth factor; CV, type V collagen; CXCL4, angiostatin, platelet factor-4; DAMPs, damage-associated molecular patterns; dcSSc, diffuse cutaneous systemic scleroderma; DNAM-1, DNAX accessory molecule-1 (i.e., CD226); ECM, extracellular matrix; EC(s), endocannabinoid(s); ECS, endocannabinoid system; EDNR-A or -B, gene that encodes endothelin receptor type A or B; Egr-1, early response gene 1; eNOS, endothelial nitric oxide synthase; ERK, extracellular signal-regulated kinase; ET-1, endothelin-1; EVI, evenness interrupted, in reference to cell surface multipass transmembrane protein; FAAH, fatty acid amide hydrolase; FasL, Fas ligand; FGF, fibroblast growth factor; FH-1, human fetal lung fibroblast line; Fli(1), friend leukemia integration; Fra-2, Fos-related antigen-2; FTY720, fingolimod; GATA-3, GATA binding protein-3; GMCSF, granulocyte monocyte-colony stimulating factor; GPCRs, G-protein-coupled receptors; GVHD, graft-versus-host disease; GWAS, genome-wide association studies; hCMV, human cytomegalovirus; HGF, hepatocyte growth factor; HLA, human leukocyte antigen; HMGB-1, high-mobility group box-1 protein; HMW-MAA, high molecular weight melanoma-associated antigen; HSP, heat-shock protein; IC, intracellular; ICAM-1, intercellular adhesion molecule-1; IFIT1, IFIT2, and IFITM1, interferon-regulated genes; IFN, interferon; IFNRI, type 1 interferon receptor; IGF, insulin-like growth factor; IGF-BP-5, insulin-like growth factor binding protein-5; ILCs, innate lymphoid cells; ILD, interstitial lung disease; IP, interferon-inducible protein; IPAH, idiopathic PAH; IPF, idiopathic pulmonary fibrosis; IRAK1, interleukin-1 receptor associated kinase 1; IRF, interferon regulatory factors; IRFSNP, interferon regulator factor, SNP (see entry); ITGAM, integrin αM; ITGAX, integrin αX; JAMs, junctional adhesion molecules; JNK, c-Jun N-terminal kinase; KCNA5, potassium voltage-gated channel, shaker-related subfamily, member 5 gene; kDA, kilo Dalton, measurement of; LAP, latency-associated peptides; lcSSc, limited cutaneous systemic scleroderma; LLC, large latent complex; LPA, lysophosphatidic acid; LPS, lipopolysaccharides; LTBP, latent TGF-β1-binding protein; MAGL, monoacylglycerol lipase; MAPK, mitogen-activated protein kinase; MCP-3, monocyte chemoattractant protein-3; MCR1, melanocortin receptor 1, gene found in yeast; MECP2, methyl-CpG-binding protein 2 gene; MHC, major histocompatibility complex; MIF, macrophage migration inhibitory factor; MIP, macrophage inflammatory protein; miRNA, micro RNA (miR); MMP, matrix metalloproteinase; mPGES-1, microsomal prostaglandin E2 synthase 1; mRNA, messenger RNA; MRSS, modified Rodnan skin score; MS, multiple sclerosis; MVEC, microvascular endothelial cells; MW, molecular weight; NAG-2, MVEC surface protein tetraspan novel antigen-2; NLR, a pattern recognition system (NOD)-like receptor family; NO, nitric oxide; NOD, nucleotide-binding and oligomerization domain; NOTCH, neurogenic locus notch homolog 4 protein encoded by the NOTCH4 gene, and an evolutionarily conserved pathway in multicellular organisms that regulates cell-fate determination during development and maintains adult tissue homeostasis; NSIP, non-specific interstitial pneumonia; PAI, plasminogen activator inhibitor; PAH, pulmonary arterial hypertension; PAMPs, pathogen-associated molecular patterns; PAR, protease-activated receptor; PASMC, pulmonary artery smooth muscle cells; PBMC, peripheral blood mononuclear cell; PDGF/PDGFR, platelet-derived growth factor/receptor; PECAM, platelet/endothelial cell adhesion molecule; PG, prostaglandin; PGF, placental growth factor; PG-Gs, microsomal prostaglandins; PI-3, phosphatidylinositol 3; PLD4, phospholipase D family member 4; PPARγ, peroxisome proliferation-activated receptor gamma; PRR, a pattern recognition receptor system in cell cytoplasm; PSD3, pleckstrin and Sec7 domain containing 3 gene; PTEN, phosphatase and tensin homolog; PTPN22, protein tyrosine phosphatase non-receptor type 22; PXK, *paraxylene*–*orthoxylene* (phox homology) domain containing serine/threonine kinase; RA, rheumatoid arthritis; RANTES, regulated upon activation, normal T-cell expressed and secreted; RGS-5, regulator of G protein signaling 5; RLRs, RIG-I-like receptors; RORγt, RAR-related orphan nuclear receptor gamma transcription factor; RP, Raynaud’s phenomenon; R-Smads, receptor-regulated Smads; rTregs, resting Tregs; S1P/S1P_1_, sphingosine 1-phosphate (type 1 receptor); scl GVHD, sclerodermatoses GVHD; Serpine 1, plasminogen activator inhibitor; Siglec-1, CD169, sialoadhesin; siRNA, small interfering RNA; SLE, systemic lupus erythematosus; Smad, small mother against decapentaplegic family of transcription factors; Snail-1, a protein of the C2H2-type zinc-finger family that regulates transcription; SNPs, single nucleotide polymorphisms; sRAGE, soluble advanced glycation end products; SSc, systemic scleroderma; STAT, signal transducer and activator of transcription; TG, transgenic; TGF-β, transforming growth factor beta; TIMP-1 or -2, tissue inhibition of metalloproteinase-1 or -2; TLRs, toll-like receptors; TNFAIP3, tumor necrosis factor alpha-induced protein-3; TNFSF4, tumor necrosis factor superfamily member 4 gene; TNIP1, TNFAIP3 interacting protein-1; tPA, tissue t plasminogen activator; Tregs, T regulatory cells; TRPV, transient receptor potential vanilloid; Tsk-1 (Tsk-1/+), tight skin; TSP-1 or TSP-2, thrombospondin-1 or -2; UPAR, urokinase-type plasminogen activator receptor; VDR, vitamin D receptor; VE, vascular endothelial; VEGF, vascular endothelial growth factor; VitD, Vitamin D; VW, von Willebrand Factor; Wnt, proteins that form a family of highly conserved secreted signaling molecules that regulate cell-to-cell interactions during embryogenesis; WT, wild-type.

## References

[1] Jimenez SA. Role of endothelial to mesenchymal transition in the pathogenesis of the vascular alterations in systemic sclerosis. ISRN Rheumatol (2013) 2013:835948. 10.1155/2013/835948 24175099 PMC3794556

[2] Wollheim FA. Classification of systemic sclerosis. Visions and reality. Rheumatology (2005) 44(10):1212–6. 10.1093/rheumatology/keh671 15870151

[3] Hochberg MC Silman AJ Smolen JS Weinblatt ME Weisman MH Rheumatology. 5th ed Philadelphia, PA: Mosby (2010). 2228 p.

[4] Koenig M Joyal F Fritzler MJ Roussin A Abrahamowicz M Boire G Autoantibodies and microvascular damage are independent predictive factors for the progression of Raynaud’s phenomenon to systemic sclerosis: a twenty-year prospective study of 586 patients, with validation of proposed criteria for early systemic sclerosis. Arthritis Rheum (2008) 58(12):3902–12. 10.1002/art.24038 19035499

[5] Pattanaik D Brown M Postlethwaite AE Vascular involvement in systemic sclerosis (scleroderma). J Inflamm Res (2011) 4:105–25. 10.2147/JIR.S18145 22096374 PMC3218751

[6] van den Hoogen F Khanna D Fransen J Johnson SR Baron M Tyndall A 2013 classification criteria for systemic sclerosis: an American College of Rheumatology/European league against rheumatism collaborative initiative. Arthritis Rheum (2013) 65(11):2737–47. 10.1002/art.38098 24122180 PMC3930146

[7] Arnson Y Amital H Guiducci S Matucci-Cerinic M Valentini G Barzilai O The role of infections in the immunopathogenesis of systemic sclerosis – evidence from serological studies. Ann N Y Acad Sci (2009) 1173:627–32. 10.1111/j.1749-6632.2009.04808.x 19758208

[8] Fattal I Shental N Molad Y Gabrielli A Pokroy-Shapira E Oren S Epstein-Barr virus antibodies mark systemic lupus erythematosus and scleroderma patients negative for anti-DNA. Immunology (2014) 141(2):276–85. 10.1111/imm.12200 24164500 PMC3904248

[9] Catrina AI Deane KD Scher JU. Gene, environment, microbiome and mucosal immune tolerance in rheumatoid arthritis. Rheumatology (2014). 10.1093/rheumatology/keu469 25539828 PMC4746430

[10] Arron ST Dimon MT Li Z Johnson ME A Wood T Feeney L High *Rhodotorula* sequences in skin transcriptome of patients with diffuse systemic sclerosis. J Invest Dermatol (2014) 134(8):2138–45. 10.1038/jid.2014.127 24608988 PMC4102619

[11] Luo Y Wang Y Wang Q Xiao R Lu Q Systemic sclerosis: genetics and epigenetics. J Autoimmun (2013) 41:161–7. 10.1016/j.jaut.2013.01.012 23415078

[12] Arnett FC Cho M Chatterjee S Aguilar MB Reveille JD Mayes MD. Familial occurrence frequencies and relative risks for systemic sclerosis (scleroderma) in three United States cohorts. Arthritis Rheum (2001) 44(6):1359–62. 10.1002/1529-0131(200106)44:6<1359::AID-ART228>3.0.CO;2-S 11407695

[13] Broen JC Coenen MJ Radstake TR. Genetics of systemic sclerosis: an update. Curr Rheumatol Rep (2012) 14(1):11–21. 10.1007/s11926-011-0221-7 22102179 PMC3253992

[14] Mayes MD Bossini-Castillo L Gorlova O Martin JE Zhou X Chen WV Immunochip analysis identifies multiple susceptibility loci for systemic sclerosis. Am J Hum Genet (2014) 94(1):47–61. 10.1016/j.ajhg.2013.12.002 24387989 PMC3882906

[15] Polychronakos C. Fine points in mapping autoimmunity. Nat Genet (2011) 43(12):1173–4. 10.1038/ng.1015 22120051

[16] Del Rio AP Sachetto Z Sampaio-Barros PD Marques-Neto JF Londe AC Bertolo MB. HLA markers for poor prognosis in systemic sclerosis Brazilian patients. Dis Markers (2013) 35(2):73–8. 10.1155/2013/301415 24167351 PMC3774956

[17] Gladman DD Kung TN Siannis F Pellett F Farewell VT Lee P. HLA markers for susceptibility and expression in scleroderma. J Rheumatol (2005) 32(8):1481–7. 16078323

[18] Arnett FC Howard RF Tan F Moulds JM Bias WB Durban E Increased prevalence of systemic sclerosis in a native American tribe in Oklahoma. Association with an Amerindian HLA haplotype. Arthritis Rheum (1996) 39(8):1362–70. 10.1002/art.1780390814 8702445

[19] Zhou X Tan FK Wang N Xiong M Maghidman S Reveille JD Genome-wide association study for regions of systemic sclerosis susceptibility in a Choctaw Indian population with high disease prevalence. Arthritis Rheum (2003) 48(9):2585–92. 10.1002/art.11220 13130478

[20] Santaniello A Salazar G Lenna S Antonioli R Colombo G Beretta L HLA-B35 upregulates the production of endothelin-1 in HLA-transfected cells: a possible pathogenetic role in pulmonary hypertension. Tissue Antigens (2006) 68(3):239–44. 10.1111/j.1399-0039.2006.00657.x 16948645

[21] Grigolo B Mazzetti I Meliconi R Bazzi S Scorza R Candela M Anti-topoisomerase II alpha autoantibodies in systemic sclerosis-association with pulmonary hypertension and HLA-B35. Clin Exp Immunol (2000) 121(3):539–43. 10.1046/j.1365-2249.2000.01320.x 10971522 PMC1905723

[22] Lenna S Townsend DM Tan FK Kapanadze B Markiewicz M Trojanowska M HLA-B35 upregulates endothelin-1 and downregulates endothelial nitric oxide synthase via endoplasmic reticulum stress response in endothelial cells. J Immunol (2010) 184(9):4654–61. 10.4049/jimmunol.0903188 20335527 PMC3836507

[23] Wastowski IJ Sampaio-Barros PD Amstalden EM Palomino GM Marques-Neto JF Crispim JC HLA-G expression in the skin of patients with systemic sclerosis. J Rheumatol (2009) 36(6):1230–4. 10.3899/jrheum.080552 19369464

[24] Gorlova O Martin JE Rueda B Koeleman BP Ying J Teruel M Identification of novel genetic markers associated with clinical phenotypes of systemic sclerosis through a genome-wide association strategy. PLoS Genet (2011) 7(7):e1002178. 10.1371/journal.pgen.1002178 21779181 PMC3136437

[25] Zhou X Lee JE Arnett FC Xiong M Park MY Yoo YK HLA-DPB1 and DPB2 are genetic loci for systemic sclerosis: a genome-wide association study in Koreans with replication in North Americans. Arthritis Rheum (2009) 60(12):3807–14. 10.1002/art.24982 19950302 PMC2829245

[26] Cheng Y Wang Y Li Y Deng Y Hu J Mo X A novel human gene ZNF415 with five isoforms inhibits AP-1- and p53-mediated transcriptional activity. Biochem Biophys Res Commun (2006) 351(1):33–9. 10.1016/j.bbrc.2006.09.161 17055453

[27] Arnett FC Gourh P Shete S Ahn CW Honey RE Agarwal SK Major histocompatibility complex (MHC) class II alleles, haplotypes and epitopes which confer susceptibility or protection in systemic sclerosis: analyses in 1300 Caucasian, African-American and Hispanic cases and 1000 controls. Ann Rheum Dis (2010) 69(5):822–7. 10.1136/ard.2009.111906 19596691 PMC2916702

[28] Agarwal SK Gourh P Shete S Paz G Divecha D Reveille JD Association of interleukin 23 receptor polymorphisms with anti-topoisomerase-I positivity and pulmonary hypertension in systemic sclerosis. J Rheumatol (2009) 36(12):2715–23. 10.3899/jrheum.090421 19918037 PMC2895677

[29] Azzouz DF Rak JM Fajardy I Allanore Y Tiev KP Farge-Bancel D Comparing HLA shared epitopes in French Caucasian patients with scleroderma. PLoS One (2012) 7(5):e36870. 10.1371/journal.pone.0036870 22615829 PMC3352938

[30] Beretta L Rueda B Marchini M Santaniello A Simeon CP Fonollosa V Analysis of Class II human leucocyte antigens in Italian and Spanish systemic sclerosis. Rheumatology (2012) 51(1):52–9. 10.1093/rheumatology/ker335 22087014 PMC3276293

[31] Reveille JD Fischbach M McNearney T Friedman AW Aguilar MB Lisse J Systemic sclerosis in 3 US ethnic groups: a comparison of clinical, sociodemographic, serologic, and immunogenetic determinants. Semin Arthritis Rheum (2001) 30(5):332–46. 10.1053/sarh.2001.20268 11303306

[32] Simeon CP Fonollosa V Tolosa C Palou E Selva A Solans R Association of HLA class II genes with systemic sclerosis in Spanish patients. J Rheumatol (2009) 36(12):2733–6. 10.3899/jrheum.090377 19884273

[33] Louthrenoo W Kasitanon N Wichainun R Wangkaew S Sukitawut W Ohnogi Y Association of HLA-DRB1*15:02 and DRB5*01:02 allele with the susceptibility to systemic sclerosis in Thai patients. Rheumatol Int (2013) 33(8):2069–77. 10.1007/s00296-013-2686-3 23404077

[34] Nguyen B Mayes MD Arnett FC del Junco D Reveille JD Gonzalez EB HLA-DRB1*0407 and *1304 are risk factors for scleroderma renal crisis. Arthritis Rheum (2011) 63(2):530–4. 10.1002/art.30111 21280007 PMC3048905

[35] Dieude P Wipff J Guedj M Ruiz B Melchers I Hachulla E BANK1 is a genetic risk factor for diffuse cutaneous systemic sclerosis and has additive effects with IRF5 and STAT4. Arthritis Rheum (2009) 60(11):3447–54. 10.1002/art.24885 19877059

[36] Rueda B Gourh P Broen J Agarwal SK Simeon C Ortego-Centeno N BANK1 functional variants are associated with susceptibility to diffuse systemic sclerosis in Caucasians. Ann Rheum Dis (2010) 69(4):700–5. 10.1136/ard.2009.118174 19815934 PMC2975737

[37] Dawidowicz K Dieude P Avouac J Wipff J Hachulla E Diot E Association study of B-cell marker gene polymorphisms in European Caucasian patients with systemic sclerosis. Clin Exp Rheumatol (2011) 29(5):839–42. 21961844

[38] Dieude P Guedj M Wipff J Avouac J Fajardy I Diot E Association between the IRF5 rs2004640 functional polymorphism and systemic sclerosis: a new perspective for pulmonary fibrosis. Arthritis Rheum (2009) 60(1):225–33. 10.1002/art.24183 19116937

[39] Assassi S Radstake TR Mayes MD Martin J. Genetics of scleroderma: implications for personalized medicine? BMC Med (2013) 11:9. 10.1186/1741-7015-11-9 23311619 PMC3568008

[40] Wang J Yi L Guo X Liu M Li H Zou H Association of the IRF5 SNP rs2004640 with systemic sclerosis in Han Chinese. Int J Immunopathol Pharmacol (2014) 27(4):635–8. 25572744 10.1177/039463201402700420

[41] Sharif R Mayes MD Tan FK Gorlova OY Hummers LK Shah AA IRF5 polymorphism predicts prognosis in patients with systemic sclerosis. Ann Rheum Dis (2012) 71(7):1197–202. 10.1136/annrheumdis-2011-200901 22440820 PMC3375372

[42] Carmona FD Cenit MC Diaz-Gallo LM Broen JC Simeon CP Carreira PE New insight on the Xq28 association with systemic sclerosis. Ann Rheum Dis (2013) 72(12):2032–8. 10.1136/annrheumdis-2012-202742 23444193 PMC3818491

[43] Dieude P Dawidowicz K Guedj M Legrain Y Wipff J Hachulla E Phenotype-haplotype correlation of IRF5 in systemic sclerosis: role of 2 haplotypes in disease severity. J Rheumatol (2010) 37(5):987–92. 10.3899/jrheum.091163 20231204

[44] Tang L Chen B Ma B Nie S. Association between IRF5 polymorphisms and autoimmune diseases: a meta-analysis. Genet Mol Res (2014) 13(2):4473–85. 10.4238/2014.June.16.6 25036352

[45] Ito I Kawaguchi Y Kawasaki A Hasegawa M Ohashi J Hikami K Association of a functional polymorphism in the IRF5 region with systemic sclerosis in a Japanese population. Arthritis Rheum (2009) 60(6):1845–50. 10.1002/art.24600 19479858

[46] Zochling J Newell F Charlesworth JC Leo P Stankovich J Cortes A An Immunochip-based interrogation of scleroderma susceptibility variants identifies a novel association at DNASE1L3. Arthritis Res Ther (2014) 16(5):438. 10.1186/s13075-014-0438-8 25332064 PMC4230517

[47] Martin JE Assassi S Diaz-Gallo LM Broen JC Simeon CP Castellvi I A systemic sclerosis and systemic lupus erythematosus pan-meta-GWAS reveals new shared susceptibility loci. Hum Mol Genet (2013) 22(19):4021–9. 10.1093/hmg/ddt248 23740937 PMC3766185

[48] Mayes MD. The genetics of scleroderma: looking into the postgenomic era. Curr Opin Rheumatol (2012) 24(6):677–84. 10.1097/BOR.0b013e328358575b 23026857 PMC3678376

[49] Radstake TR Gorlova O Rueda B Martin JE Alizadeh BZ Palomino-Morales R Genome-wide association study of systemic sclerosis identifies CD247 as a new susceptibility locus. Nat Genet (2010) 42(5):426–9. 10.1038/ng.565 20383147 PMC2861917

[50] Rueda B Broen J Simeon C Hesselstrand R Diaz B Suarez H The STAT4 gene influences the genetic predisposition to systemic sclerosis phenotype. Hum Mol Genet (2009) 18(11):2071–7. 10.1093/hmg/ddp119 19286670

[51] Tsuchiya N Kawasaki A Hasegawa M Fujimoto M Takehara K Kawaguchi Y Association of STAT4 polymorphism with systemic sclerosis in a Japanese population. Ann Rheum Dis (2009) 68(8):1375–6. 10.1136/ard.2009.111310 19605749

[52] Yi L Wang JC Guo XJ Gu YH Tu WZ Guo G STAT4 is a genetic risk factor for systemic sclerosis in a Chinese population. Int J Immunopathol Pharmacol (2013) 26(2):473–8. 23755762 10.1177/039463201302600220 PMC4105920

[53] Gourh P Agarwal SK Divecha D Assassi S Paz G Arora-Singh RK Polymorphisms in TBX21 and STAT4 increase the risk of systemic sclerosis: evidence of possible gene-gene interaction and alterations in Th1/Th2 cytokines. Arthritis Rheum (2009) 60(12):3794–806. 10.1002/art.24958 19950257 PMC2998060

[54] Liang YL Wu H Shen X Li PQ Yang XQ Liang L Association of STAT4 rs7574865 polymorphism with autoimmune diseases: a meta-analysis. Mol Biol Rep (2012) 39(9):8873–82. 10.1007/s11033-012-1754-1 22714917

[55] Gourh P Tan FK Assassi S Ahn CW McNearney TA Fischbach M Association of the PTPN22 R620W polymorphism with anti-topoisomerase I- and anticentromere antibody-positive systemic sclerosis. Arthritis Rheum (2006) 54(12):3945–53. 10.1002/art.22196 17133608

[56] Lee YH Choi SJ Ji JD Song GG. The association between the PTPN22 C1858T polymorphism and systemic sclerosis: a meta-analysis. Mol Biol Rep (2012) 39(3):3103–8. 10.1007/s11033-011-1074-x 21688149

[57] Diaz-Gallo LM Gourh P Broen J Simeon C Fonollosa V Ortego-Centeno N Analysis of the influence of PTPN22 gene polymorphisms in systemic sclerosis. Ann Rheum Dis (2011) 70(3):454–62. 10.1136/ard.2010.130138 21131644 PMC3170726

[58] Dieude P Guedj M Wipff J Avouac J Hachulla E Diot E The PTPN22 620W allele confers susceptibility to systemic sclerosis: findings of a large case-control study of European Caucasians and a meta-analysis. Arthritis Rheum (2008) 58(7):2183–8. 10.1002/art.23601 18576360

[59] Gourh P Arnett FC Tan FK Assassi S Divecha D Paz G Association of TNFSF4 (OX40L) polymorphisms with susceptibility to systemic sclerosis. Ann Rheum Dis (2010) 69(3):550–5. 10.1136/ard.2009.116434 19778912 PMC2927683

[60] Bossini-Castillo L Broen JC Simeon CP Beretta L Vonk MC Ortego-Centeno N A replication study confirms the association of TNFSF4 (OX40L) polymorphisms with systemic sclerosis in a large European cohort. Ann Rheum Dis (2011) 70(4):638–41. 10.1136/ard.2010.141838 21187296

[61] Coustet B Bouaziz M Dieude P Guedj M Bossini-Castillo L Agarwal S Independent replication and meta analysis of association studies establish TNFSF4 as a susceptibility gene preferentially associated with the subset of anticentromere-positive patients with systemic sclerosis. J Rheumatol (2012) 39(5):997–1003. 10.3899/jrheum.111270 22422496 PMC3687343

[62] Coustet B Dieude P Guedj M Bouaziz M Avouac J Ruiz B C8orf13-BLK is a genetic risk locus for systemic sclerosis and has additive effects with BANK1: results from a large French cohort and meta-analysis. Arthritis Rheum (2011) 63(7):2091–6. 10.1002/art.30379 21480188

[63] Gourh P Agarwal SK Martin E Divecha D Rueda B Bunting H Association of the C8orf13-BLK region with systemic sclerosis in North-American and European populations. J Autoimmun (2010) 34(2):155–62. 10.1016/j.jaut.2009.08.014 19796918 PMC2821978

[64] Ito I Kawaguchi Y Kawasaki A Hasegawa M Ohashi J Kawamoto M Association of the FAM167A-BLK region with systemic sclerosis. Arthritis Rheum (2010) 62(3):890–5. 10.1002/art.27303 20131239

[65] Diaz-Gallo LM Simeon CP Broen JC Ortego-Centeno N Beretta L Vonk MC Implication of IL-2/IL-21 region in systemic sclerosis genetic susceptibility. Ann Rheum Dis (2013) 72(7):1233–8. 10.1136/annrheumdis-2012-202357 23172754 PMC3887514

[66] Martin JE Carmona FD Broen JC Simeon CP Vonk MC Carreira P The autoimmune disease-associated IL2RA locus is involved in the clinical manifestations of systemic sclerosis. Genes Immun (2012) 13(2):191–6. 10.1038/gene.2011.72 22012429

[67] Bossini-Castillo L Martin JE Broen J Gorlova O Simeon CP Beretta L A GWAS follow-up study reveals the association of the IL12RB2 gene with systemic sclerosis in Caucasian populations. Hum Mol Genet (2012) 21(4):926–33. 10.1093/hmg/ddr522 22076442 PMC3298110

[68] Terao C Ohmura K Kawaguchi Y Nishimoto T Kawasaki A Takehara K PLD4 as a novel susceptibility gene for systemic sclerosis in a Japanese population. Arthritis Rheum (2013) 65(2):472–80. 10.1002/art.37777 23124809

[69] Dieude P Boileau C Guedj M Avouac J Ruiz B Hachulla E Independent replication establishes the CD247 gene as a genetic systemic sclerosis susceptibility factor. Ann Rheum Dis (2011) 70(9):1695–6. 10.1136/ard.2010.147009 21474487

[70] Martin JE Broen JC Carmona FD Teruel M Simeon CP Vonk MC Identification of CSK as a systemic sclerosis genetic risk factor through genome wide association study follow-up. Hum Mol Genet (2012) 21(12):2825–35. 10.1093/hmg/dds099 22407130 PMC3368627

[71] Lopez-Isac E Bossini-Castillo L Guerra SG Denton C Fonseca C Assassi S Identification of IL12RB1 as a novel systemic sclerosis susceptibility locus. Arthritis Rheumatol (2014) 66(12):3521–3. 10.1002/art.38870 25199642 PMC4245383

[72] Dieude P Bouaziz M Guedj M Riemekasten G Airo P Muller M Evidence of the contribution of the X chromosome to systemic sclerosis susceptibility: association with the functional IRAK1 196Phe/532Ser haplotype. Arthritis Rheum (2011) 63(12):3979–87. 10.1002/art.30640 21898345

[73] Allanore Y Saad M Dieude P Avouac J Distler JH Amouyel P Genome-wide scan identifies TNIP1, PSORS1C1, and RHOB as novel risk loci for systemic sclerosis. PLoS Genet (2011) 7(7):e1002091. 10.1371/journal.pgen.1002091 21750679 PMC3131285

[74] Bossini-Castillo L Martin JE Broen J Simeon CP Beretta L Gorlova OY Confirmation of TNIP1 but not RHOB and PSORS1C1 as systemic sclerosis risk factors in a large independent replication study. Ann Rheum Dis (2013) 72(4):602–7. 10.1136/annrheumdis-2012-201888 22896740 PMC3887516

[75] Wu SP Leng L Feng Z Liu N Zhao H McDonald C Macrophage migration inhibitory factor promoter polymorphisms and the clinical expression of scleroderma. Arthritis Rheum (2006) 54(11):3661–9. 10.1002/art.22179 17075815

[76] Salim PH Jobim M Bredemeier M Chies JA Brenol JC Jobim LF Interleukin-10 gene promoter and NFKB1 promoter insertion/deletion polymorphisms in systemic sclerosis. Scand J Immunol (2013) 77(2):162–8. 10.1111/sji.12020 23237063

[77] Bossini-Castillo L Simeon CP Beretta L Broen JC Vonk MC Rios-Fernandez R A multicenter study confirms CD226 gene association with systemic sclerosis-related pulmonary fibrosis. Arthritis Res Ther (2012) 14(2):R85. 10.1186/ar3809 22531499 PMC3446459

[78] Todd JA Walker NM Cooper JD Smyth DJ Downes K Plagnol V Robust associations of four new chromosome regions from genome-wide analyses of type 1 diabetes. Nat Genet (2007) 39(7):857–64. 10.1038/ng2068 17554260 PMC2492393

[79] Hafler JP Maier LM Cooper JD Plagnol V Hinks A Simmonds MJ CD226 Gly307Ser association with multiple autoimmune diseases. Genes Immun (2009) 10(1):5–10. 10.1038/gene.2008.82 18971939 PMC2635550

[80] Dieude P Guedj M Truchetet ME Wipff J Revillod L Riemekasten G Association of the CD226 Ser(307) variant with systemic sclerosis: evidence of a contribution of costimulation pathways in systemic sclerosis pathogenesis. Arthritis Rheum (2011) 63(4):1097–105. 10.1002/art.30204 21162102

[81] Wei J Bhattacharyya S Varga J. Peroxisome proliferator-activated receptor gamma: innate protection from excessive fibrogenesis and potential therapeutic target in systemic sclerosis. Curr Opin Rheumatol (2010) 22(6):671–6. 10.1097/BOR.0b013e32833de1a7 20693905 PMC4536822

[82] Wu M Melichian DS Chang E Warner-Blankenship M Ghosh AK Varga J. Rosiglitazone abrogates bleomycin-induced scleroderma and blocks profibrotic responses through peroxisome proliferator-activated receptor-gamma. Am J Pathol (2009) 174(2):519–33. 10.2353/ajpath.2009.080574 19147827 PMC2630560

[83] Lopez-Isac E Bossini-Castillo L Simeon CP Egurbide MV Alegre-Sancho JJ Callejas JL A genome-wide association study follow-up suggests a possible role for PPARG in systemic sclerosis susceptibility. Arthritis Res Ther (2014) 16(1):R6. 10.1186/ar4432 24401602 PMC3978735

[84] Anaya JM Kim-Howard X Prahalad S Chernavsky A Canas C Rojas-Villarraga A Evaluation of genetic association between an ITGAM non-synonymous SNP (rs1143679) and multiple autoimmune diseases. Autoimmun Rev (2012) 11(4):276–80. 10.1016/j.autrev.2011.07.007 21840425 PMC3224188

[85] Carmona FD Serrano A Rodriguez-Rodriguez L Castaneda S Miranda-Filloy JA Morado IC A nonsynonymous functional variant of the ITGAM gene is not involved in biopsy-proven giant cell arteritis. J Rheumatol (2011) 38(12):2598–601. 10.3899/jrheum.110685 21965647

[86] Koumakis E Giraud M Dieude P Cohignac V Cuomo G Airo P Brief report: candidate gene study in systemic sclerosis identifies a rare and functional variant of the TNFAIP3 locus as a risk factor for polyautoimmunity. Arthritis Rheum (2012) 64(8):2746–52. 10.1002/art.34490 22488580

[87] Carmona FD Gutala R Simeon CP Carreira P Ortego-Centeno N Vicente-Rabaneda E Novel identification of the IRF7 region as an anticentromere autoantibody propensity locus in systemic sclerosis. Ann Rheum Dis (2012) 71(1):114–9. 10.1136/annrheumdis-2011-200275 21926187 PMC3369428

[88] Rueda B Broen J Torres O Simeon C Ortego-Centeno N Schrijvenaars MM The interleukin 23 receptor gene does not confer risk to systemic sclerosis and is not associated with systemic sclerosis disease phenotype. Ann Rheum Dis (2009) 68(2):253–6. 10.1136/ard.2008.096719 18713787

[89] Farago B Magyari L Safrany E Csongei V Jaromi L Horvatovich K Functional variants of interleukin-23 receptor gene confer risk for rheumatoid arthritis but not for systemic sclerosis. Ann Rheum Dis (2008) 67(2):248–50. 10.1136/ard.2007.072819 17606463

[90] Broen JC Bossini-Castillo L van Bon L Vonk MC Knaapen H Beretta L A rare polymorphism in the gene for toll-like receptor 2 is associated with systemic sclerosis phenotype and increases the production of inflammatory mediators. Arthritis Rheum (2012) 64(1):264–71. 10.1002/art.33325 21905008

[91] Manetti M Allanore Y Revillod L Fatini C Guiducci S Cuomo G A genetic variation located in the promoter region of the UPAR (CD87) gene is associated with the vascular complications of systemic sclerosis. Arthritis Rheum (2011) 63(1):247–56. 10.1002/art.30101 20967855

[92] Manetti M Ibba-Manneschi L Fatini C Guiducci S Cuomo G Bonino C Association of a functional polymorphism in the matrix metalloproteinase-12 promoter region with systemic sclerosis in an Italian population. J Rheumatol (2010) 37(9):1852–7. 10.3899/jrheum.100237 20595276

[93] Zhu H Luo H Li Y Zhou Y Jiang Y Chai J MicroRNA-21 in scleroderma fibrosis and its function in TGF-beta-regulated fibrosis-related genes expression. J Clin Immunol (2013) 33(6):1100–9. 10.1007/s10875-013-9896-z 23657402

[94] Zhu H Li Y Qu S Luo H Zhou Y Wang Y MicroRNA expression abnormalities in limited cutaneous scleroderma and diffuse cutaneous scleroderma. J Clin Immunol (2012) 32(3):514–22. 10.1007/s10875-011-9647-y 22307526

[95] Tanaka S Suto A Ikeda K Sanayama Y Nakagomi D Iwamoto T Alteration of circulating miRNAs in SSc: miR-30b regulates the expression of PDGF receptor beta. Rheumatology (2013) 52(11):1963–72. 10.1093/rheumatology/ket254 23893664

[96] Kawashita Y Jinnin M Makino T Kajihara I Makino K Honda N Circulating miR-29a levels in patients with scleroderma spectrum disorder. J Dermatol Sci (2011) 61(1):67–9. 10.1016/j.jdermsci.2010.11.007 21129921

[97] Maurer B Stanczyk J Jungel A Akhmetshina A Trenkmann M Brock M MicroRNA-29, a key regulator of collagen expression in systemic sclerosis. Arthritis Rheum (2010) 62(6):1733–43. 10.1002/art.27443 20201077

[98] Takemoto R Jinnin M Wang Z Kudo H Inoue K Nakayama W Hair miR-29a levels are decreased in patients with scleroderma. Exp Dermatol (2013) 22(12):832–3. 10.1111/exd.12245 24107002

[99] Honda N Jinnin M Kajihara I Makino T Makino K Masuguchi S TGF-beta-mediated downregulation of microRNA-196a contributes to the constitutive upregulated type I collagen expression in scleroderma dermal fibroblasts. J Immunol (2012) 188(7):3323–31. 10.4049/jimmunol.1100876 22379029

[100] Wang Z Jinnin M Kudo H Inoue K Nakayama W Honda N Detection of hair-microRNAs as the novel potent biomarker: evaluation of the usefulness for the diagnosis of scleroderma. J Dermatol Sci (2013) 72(2):134–41. 10.1016/j.jdermsci.2013.06.018 23890704

[101] Honda N Jinnin M Kira-Etoh T Makino K Kajihara I Makino T miR-150 down-regulation contributes to the constitutive type I collagen overexpression in scleroderma dermal fibroblasts via the induction of integrin beta3. Am J Pathol (2013) 182(1):206–16. 10.1016/j.ajpath.2012.09.023 23159943

[102] Etoh M Jinnin M Makino K Yamane K Nakayama W Aoi J microRNA-7 down-regulation mediates excessive collagen expression in localized scleroderma. Arch Dermatol Res (2013) 305(1):9–15. 10.1007/s00403-012-1287-4 22965811

[103] Kajihara I Jinnin M Yamane K Makino T Honda N Igata T Increased accumulation of extracellular thrombospondin-2 due to low degradation activity stimulates type I collagen expression in scleroderma fibroblasts. Am J Pathol (2012) 180(2):703–14. 10.1016/j.ajpath.2011.10.030 22142808

[104] Makino K Jinnin M Hirano A Yamane K Eto M Kusano T The downregulation of microRNA let-7a contributes to the excessive expression of type I collagen in systemic and localized scleroderma. J Immunol (2013) 190(8):3905–15. 10.4049/jimmunol.1200822 23509348

[105] Nakashima T Jinnin M Yamane K Honda N Kajihara I Makino T Impaired IL-17 signaling pathway contributes to the increased collagen expression in scleroderma fibroblasts. J Immunol (2012) 188(8):3573–83. 10.4049/jimmunol.1100591 22403442

[106] Makino K Jinnin M Kajihara I Honda N Sakai K Masuguchi S Circulating miR-142-3p levels in patients with systemic sclerosis. Clin Exp Dermatol (2012) 37(1):34–9. 10.1111/j.1365-2230.2011.04158.x 21883400

[107] Sing T Jinnin M Yamane K Honda N Makino K Kajihara I microRNA-92a expression in the sera and dermal fibroblasts increases in patients with scleroderma. Rheumatology (2012) 51(9):1550–6. 10.1093/rheumatology/kes120 22661558

[108] Favalli E Ingegnoli F Zeni S Fare M Fantini F [HLA typing in systemic sclerosis]. Reumatismo (2001) 53(3):210–4. [in Italian]. 12167973 10.4081/reumatismo.2001.210

[109] Agarwal SK The genetics of systemic sclerosis. Discov Med (2010) 10(51):134–43. 20807474 PMC3803145

[110] Assassi S Del Junco D Sutter K McNearney TA Reveille JD Karnavas A Clinical and genetic factors predictive of mortality in early systemic sclerosis. Arthritis Rheum (2009) 61(10):1403–11. 10.1002/art.24734 19790132 PMC2883167

[111] Sharif R Fritzler MJ Mayes MD Gonzalez EB McNearney TA Draeger H Anti-fibrillarin antibody in African American patients with systemic sclerosis: immunogenetics, clinical features, and survival analysis. J Rheumatol (2011) 38(8):1622–30. 10.3899/jrheum.110071 21572159 PMC3149738

[112] Wang J Guo X Yi L Guo G Tu W Wu W Association of HLA-DPB1 with scleroderma and its clinical features in Chinese population. PLoS One (2014) 9(1):e87363. 10.1371/journal.pone.0087363 24498086 PMC3909094

[113] Wang J Yi L Guo X He D Li H Guo G Lack of association of the CD247 SNP rs2056626 with systemic sclerosis in Han Chinese. Open Rheumatol J (2014) 8:43–5. 10.2174/1874312901408010043 25317213 PMC4192828

[114] Assassi S Mayes MD Arnett FC Gourh P Agarwal SK McNearney TA Systemic sclerosis and lupus: points in an interferon-mediated continuum. Arthritis Rheum (2010) 62(2):589–98. 10.1002/art.27224 20112391 PMC2879587

[115] Kottyan LC Zoller EE Bene J Lu X Kelly JA Rupert AM The IRF5-TNPO3 association with systemic lupus erythematosus has two components that other autoimmune disorders variably share. Hum Mol Genet (2015) 24(2):582–96. 10.1093/hmg/ddu455 25205108 PMC4275071

[116] Nicolls MR Taraseviciene-Stewart L Rai PR Badesch DB Voelkel NF. Autoimmunity and pulmonary hypertension: a perspective. Eur Respir J (2005) 26(6):1110–8. 10.1183/09031936.05.00045705 16319344

[117] George PM Oliver E Dorfmuller P Dubois OD Reed DM Kirkby NS Evidence for the involvement of type I interferon in pulmonary arterial hypertension. Circ Res (2014) 114(4):677–88. 10.1161/CIRCRESAHA.114.302221 24334027 PMC4006084

[118] Dhillon S Kaker A Dosanjh A Japra D Vanthiel DH. Irreversible pulmonary hypertension associated with the use of interferon alpha for chronic hepatitis C. Dig Dis Sci (2010) 55(6):1785–90. 10.1007/s10620-010-1220-7 20411421 PMC2882564

[119] Ledinek AH Jazbec SS Drinovec I Rot U. Pulmonary arterial hypertension associated with interferon beta treatment for multiple sclerosis: a case report. Mult Scler (2009) 15(7):885–6. 10.1177/1352458509104593 19465452

[120] Speich R Jenni R Opravil M Pfab M Russi EW Primary pulmonary hypertension in HIV infection. Chest (1991) 100(5):1268–71. 10.1378/chest.100.5.1268 1935280

[121] Mukerjee D St George D Coleiro B Knight C Denton CP Davar J Prevalence and outcome in systemic sclerosis associated pulmonary arterial hypertension: application of a registry approach. Ann Rheum Dis (2003) 62(11):1088–93. 10.1136/ard.62.11.1088 14583573 PMC1754353

[122] Eloranta ML Franck-Larsson K Lovgren T Kalamajski S Ronnblom A Rubin K Type I interferon system activation and association with disease manifestations in systemic sclerosis. Ann Rheum Dis (2010) 69(7):1396–402. 10.1136/ard.2009.121400 20472592

[123] Christmann RB Hayes E Pendergrass S Padilla C Farina G Affandi AJ Interferon and alternative activation of monocyte/macrophages in systemic sclerosis-associated pulmonary arterial hypertension. Arthritis Rheum (2011) 63(6):1718–28. 10.1002/art.30318 21425123 PMC4030759

[124] Marion TN Postlethwaite AE. Chance, genetics, and the heterogeneity of disease and pathogenesis in systemic lupus erythematosus. Semin Immunopathol (2014) 36(5):495–517. 10.1007/s00281-014-0440-x 25102991

[125] York MR Nagai T Mangini AJ Lemaire R van Seventer JM Lafyatis R. A macrophage marker, Siglec-1, is increased on circulating monocytes in patients with systemic sclerosis and induced by type I interferons and toll-like receptor agonists. Arthritis Rheum (2007) 56(3):1010–20. 10.1002/art.22382 17328080

[126] Pendergrass SA Hayes E Farina G Lemaire R Farber HW Whitfield ML Limited systemic sclerosis patients with pulmonary arterial hypertension show biomarkers of inflammation and vascular injury. PLoS One (2010) 5(8):e12106. 10.1371/journal.pone.0012106 20808962 PMC2923145

[127] Maiti AK Kim-Howard X Viswanathan P Guillen L Rojas-Villarraga A Deshmukh H Confirmation of an association between rs6822844 at the Il2-Il21 region and multiple autoimmune diseases: evidence of a general susceptibility locus. Arthritis Rheum (2010) 62(2):323–9. 10.1002/art.27222 20112382 PMC3028384

[128] Carmona FD Simeon CP Beretta L Carreira P Vonk MC Rios-Fernandez R Association of a non-synonymous functional variant of the ITGAM gene with systemic sclerosis. Ann Rheum Dis (2011) 70(11):2050–2. 10.1136/ard.2010.148874 21571730

[129] Balada E Simeon-Aznar CP Serrano-Acedo S Martinez-Lostao L Selva-O’Callaghan A Fonollosa-Pla V Lack of association of the PTPN22 gene polymorphism R620W with systemic sclerosis. Clin Exp Rheumatol (2006) 24(3):321–4. 16870103

[130] Wipff J Allanore Y Kahan A Meyer O Mouthon L Guillevin L Lack of association between the protein tyrosine phosphatase non-receptor 22 (PTPN22)*620W allele and systemic sclerosis in the French Caucasian population. Ann Rheum Dis (2006) 65(9):1230–2. 10.1136/ard.2005.048181 16464986 PMC1798267

[131] Vaughn SE Foley C Lu X Patel ZH Zoller EE Magnusen AF Lupus risk variants in the PXK locus alter B-cell receptor internalization. Front Genet (2014) 5:450. 10.3389/fgene.2014.00450 25620976 PMC4288052

[132] Lim CP Cao X. Structure, function, and regulation of STAT proteins. Mol Biosyst (2006) 2(11):536–50. 10.1039/b606246f 17216035

[133] Watford WT Hissong BD Bream JH Kanno Y Muul L O’Shea JJ. Signaling by IL-12 and IL-23 and the immunoregulatory roles of STAT4. Immunol Rev (2004) 202:139–56. 10.1111/j.0105-2896.2004.00211.x 15546391

[134] O’Shea JJ Notarangelo LD Johnston JA Candotti F. Advances in the understanding of cytokine signal transduction: the role of Jaks and STATs in immunoregulation and the pathogenesis of immunodeficiency. J Clin Immunol (1997) 17(6):431–47. 10.1023/A:1027388508570 9418183

[135] Kaplan MH Sun YL Hoey T Grusby MJ. Impaired IL-12 responses and enhanced development of Th2 cells in Stat4-deficient mice. Nature (1996) 382(6587):174–7. 10.1038/382174a0 8700209

[136] Kaplan MH. STAT4: a critical regulator of inflammation in vivo. Immunol Res (2005) 31(3):231–42. 10.1385/IR:31:3:231 15888914

[137] Avouac J Furnrohr BG Tomcik M Palumbo K Zerr P Horn A Inactivation of the transcription factor STAT-4 prevents inflammation-driven fibrosis in animal models of systemic sclerosis. Arthritis Rheum (2011) 63(3):800–9. 10.1002/art.30171 21360510

[138] Bossini-Castillo L Simeon CP Beretta L Vonk MC Callejas-Rubio JL Espinosa G Confirmation of association of the macrophage migration inhibitory factor gene with systemic sclerosis in a large European population. Rheumatology (2011) 50(11):1976–81. 10.1093/rheumatology/ker259 21875883

[139] Sakoguchi A Nakayama W Jinnin M Wang Z Yamane K Aoi J The expression profile of the toll-like receptor family in scleroderma dermal fibroblasts. Clin Exp Rheumatol (2014) 32(6 Suppl 86):S–4–9. 24959869

[140] Shiwen X Leask A Abraham DJ Fonseca C. Endothelin receptor selectivity: evidence from in vitro and pre-clinical models of scleroderma. Eur J Clin Invest (2009) 39(Suppl 2):19–26. 10.1111/j.1365-2362.2009.02117.x 19335743

[141] Ortega Mateo A de Artinano AA. Highlights on endothelins: a review. Pharmacol Res (1997) 36(5):339–51. 10.1006/phrs.1997.0246 9441724

[142] Sakkas LI Chikanza IC Platsoucas CD. Mechanisms of disease: the role of immune cells in the pathogenesis of systemic sclerosis. Nat Clin Pract Rheumatol (2006) 2(12):679–85. 10.1038/ncprheum0346 17133253

[143] Shi-Wen X Rodriguez-Pascual F Lamas S Holmes A Howat S Pearson JD Constitutive ALK5-independent c-Jun N-terminal kinase activation contributes to endothelin-1 overexpression in pulmonary fibrosis: evidence of an autocrine endothelin loop operating through the endothelin A and B receptors. Mol Cell Biol (2006) 26(14):5518–27. 10.1128/MCB.00625-06 16809784 PMC1592704

[144] Kawaguchi Y Tochimoto A Hara M Kawamoto M Sugiura T Katsumata Y NOS2 polymorphisms associated with the susceptibility to pulmonary arterial hypertension with systemic sclerosis: contribution to the transcriptional activity. Arthritis Res Ther (2006) 8(4):R104. 10.1186/ar1984 16813666 PMC1779390

[145] Bossini-Castillo L Simeon CP Beretta L Broen J Vonk MC Callejas JL KCNA5 gene is not confirmed as a systemic sclerosis-related pulmonary arterial hypertension genetic susceptibility factor. Arthritis Res Ther (2012) 14(6):R273. 10.1186/ar4124 23270786 PMC3674598

[146] Inui M Martello G Piccolo S MicroRNA control of signal transduction. Nat Rev Mol Cell Biol (2010) 11(4):252–63. 10.1038/nrm2868 20216554

[147] Zhu S Pan W Qian Y. MicroRNA in immunity and autoimmunity. J Mol Med (2013) 91(9):1039–50. 10.1007/s00109-013-1043-z 23636510

[148] Bhattacharyya M Das M Bandyopadhyay S. miRT: a database of validated transcription start sites of human microRNAs. Genomics Proteomics Bioinformatics (2012) 10(5):310–6. 10.1016/j.gpb.2012.08.005 23200141 PMC5054196

[149] Altorok N Almeshal N Wang Y Kahaleh B. Epigenetics, the holy grail in the pathogenesis of systemic sclerosis. Rheumatology (2014). 10.1093/rheumatology/keu155 24740406

[150] Koba S Jinnin M Inoue K Nakayama W Honda N Makino K Expression analysis of multiple microRNAs in each patient with scleroderma. Exp Dermatol (2013) 22(7):489–91. 10.1111/exd.12173 23800063

[151] Cushing L Kuang PP Qian J Shao F Wu J Little F miR-29 is a major regulator of genes associated with pulmonary fibrosis. Am J Respir Cell Mol Biol (2011) 45(2):287–94. 10.1165/rcmb.2010-0323OC 20971881 PMC3175558

[152] van Rooij E Sutherland LB Thatcher JE DiMaio JM Naseem RH Marshall WS Dysregulation of microRNAs after myocardial infarction reveals a role of miR-29 in cardiac fibrosis. Proc Natl Acad Sci U S A (2008) 105(35):13027–32. 10.1073/pnas.0805038105 18723672 PMC2529064

[153] Xiao J Meng XM Huang XR Chung AC Feng YL Hui DS miR-29 inhibits bleomycin-induced pulmonary fibrosis in mice. Mol Ther (2012) 20(6):1251–60. 10.1038/mt.2012.36 22395530 PMC3369297

[154] Lewis BP Burge CB Bartel DP. Conserved seed pairing, often flanked by adenosines, indicates that thousands of human genes are microRNA targets. Cell (2005) 120(1):15–20. 10.1016/j.cell.2004.12.035 15652477

[155] O’Reilly S Innate immunity in systemic sclerosis pathogenesis. Clin Sci (2014) 126(5):329–37. 10.1042/CS20130367 24219159

[156] Poltorak A He X Smirnova I Liu MY Van Huffel C Du X Defective LPS signaling in C3H/HeJ and C57BL/10ScCr mice: mutations in Tlr4 gene. Science (1998) 282(5396):2085–8. 10.1126/science.282.5396.2085 9851930

[157] Okamura Y Watari M Jerud ES Young DW Ishizaka ST Rose J The extra domain A of fibronectin activates toll-like receptor 4. J Biol Chem (2001) 276(13):10229–33. 10.1074/jbc.M100099200 11150311

[158] Park JS Svetkauskaite D He Q Kim JY Strassheim D Ishizaka A Involvement of toll-like receptors 2 and 4 in cellular activation by high mobility group box 1 protein. J Biol Chem (2004) 279(9):7370–7. 10.1074/jbc.M306793200 14660645

[159] Vogl T Tenbrock K Ludwig S Leukert N Ehrhardt C van Zoelen MA Mrp8 and Mrp14 are endogenous activators of toll-like receptor 4, promoting lethal, endotoxin-induced shock. Nat Med (2007) 13(9):1042–9. 10.1038/nm1638 17767165

[160] Bhattacharyya S Kelley K Melichian DS Tamaki Z Fang F Su Y Toll-like receptor 4 signaling augments transforming growth factor-beta responses: a novel mechanism for maintaining and amplifying fibrosis in scleroderma. Am J Pathol (2013) 182(1):192–205. 10.1016/j.ajpath.2012.09.007 23141927 PMC3538029

[161] Yoshizaki A Komura K Iwata Y Ogawa F Hara T Muroi E Clinical significance of serum HMGB-1 and sRAGE levels in systemic sclerosis: association with disease severity. J Clin Immunol (2009) 29(2):180–9. 10.1007/s10875-008-9252-x 18825489

[162] Tomcik M Zerr P Pitkowski J Palumbo-Zerr K Avouac J Distler O Heat shock protein 90 (Hsp90) inhibition targets canonical TGF-beta signalling to prevent fibrosis. Ann Rheum Dis (2014) 73(6):1215–22. 10.1136/annrheumdis-2012-203095 23661493

[163] Engstrom-Laurent A Feltelius N Hallgren R Wasteson A. Raised serum hyaluronate levels in scleroderma: an effect of growth factor induced activation of connective tissue cells? Ann Rheum Dis (1985) 44(9):614–20. 10.1136/ard.44.9.614 3876080 PMC1001720

[164] Akira S Takeda K Toll-like receptor signalling. Nat Rev Immunol (2004) 4(7):499–511. 10.1038/nri1391 15229469

[165] Akira S Uematsu S Takeuchi O Pathogen recognition and innate immunity. Cell (2006) 124(4):783–801. 10.1016/j.cell.2006.02.015 16497588

[166] Kim D Peck A Santer D Patole P Schwartz SM Molitor JA Induction of interferon-alpha by scleroderma sera containing autoantibodies to topoisomerase I: association of higher interferon-alpha activity with lung fibrosis. Arthritis Rheum (2008) 58(7):2163–73. 10.1002/art.23486 18576347

[167] van den Berg TK van Die I de Lavalette CR Dopp EA Smit LD van der Meide PH Regulation of sialoadhesin expression on rat macrophages. Induction by glucocorticoids and enhancement by IFN-beta, IFN-gamma, IL-4, and lipopolysaccharide. J Immunol (1996) 157(7):3130–8. 8816424

[168] Christmann RB Sampaio-Barros P Stifano G Borges CL de Carvalho CR Kairalla R Association of Interferon- and transforming growth factor beta-regulated genes and macrophage activation with systemic sclerosis-related progressive lung fibrosis. Arthritis Rheumatol (2014) 66(3):714–25. 10.1002/art.38288 24574232 PMC4439004

[169] Wu D Hiroshima K Matsumoto S Nabeshima K Yusa T Ozaki D Diagnostic usefulness of p16/CDKN2A FISH in distinguishing between sarcomatoid mesothelioma and fibrous pleuritis. Am J Clin Pathol (2013) 139(1):39–46. 10.1309/AJCPT94JVWIHBKRD 23270897

[170] Tamura T Yanai H Savitsky D Taniguchi T. The IRF family transcription factors in immunity and oncogenesis. Annu Rev Immunol (2008) 26:535–84. 10.1146/annurev.immunol.26.021607.090400 18303999

[171] Martin JE Bossini-Castillo L Martin J. Unraveling the genetic component of systemic sclerosis. Hum Genet (2012) 131(7):1023–37. 10.1007/s00439-011-1137-z 22218928

[172] Graham RR Kozyrev SV Baechler EC Reddy MV Plenge RM Bauer JW A common haplotype of interferon regulatory factor 5 (IRF5) regulates splicing and expression and is associated with increased risk of systemic lupus erythematosus. Nat Genet (2006) 38(5):550–5. 10.1038/ng1782 16642019

[173] Graham RR Kyogoku C Sigurdsson S Vlasova IA Davies LR Baechler EC Three functional variants of IFN regulatory factor 5 (IRF5) define risk and protective haplotypes for human lupus. Proc Natl Acad Sci U S A (2007) 104(16):6758–63. 10.1073/pnas.0701266104 17412832 PMC1847749

[174] Carmona FD Martin JE Beretta L Simeon CP Carreira PE Callejas JL The systemic lupus erythematosus IRF5 risk haplotype is associated with systemic sclerosis. PLoS One (2013) 8(1):e54419. 10.1371/journal.pone.0054419 23372721 PMC3553151

[175] Tan FK Zhou X Mayes MD Gourh P Guo X Marcum C Signatures of differentially regulated interferon gene expression and vasculotrophism in the peripheral blood cells of systemic sclerosis patients. Rheumatology (2006) 45(6):694–702. 10.1093/rheumatology/kei244 16418202

[176] Dieude P Guedj M Wipff J Ruiz B Riemekasten G Airo P NLRP1 influences the systemic sclerosis phenotype: a new clue for the contribution of innate immunity in systemic sclerosis-related fibrosing alveolitis pathogenesis. Ann Rheum Dis (2011) 70(4):668–74. 10.1136/ard.2010.131243 21149496

[177] Artlett CM Sassi-Gaha S Rieger JL Boesteanu AC Feghali-Bostwick CA Katsikis PD. The inflammasome activating caspase 1 mediates fibrosis and myofibroblast differentiation in systemic sclerosis. Arthritis Rheum (2011) 63(11):3563–74. 10.1002/art.30568 21792841

[178] Gasse P Riteau N Charron S Girre S Fick L Petrilli V Uric acid is a danger signal activating NALP3 inflammasome in lung injury inflammation and fibrosis. Am J Respir Crit Care Med (2009) 179(10):903–13. 10.1164/rccm.200808-1274OC 19218193

[179] Martinez-Godinez MA Cruz-Dominguez MP Jara LJ Dominguez-Lopez A Jarillo-Luna RA Vera-Lastra O Expression of NLRP3 inflammasome, cytokines and vascular mediators in the skin of systemic sclerosis patients. Isr Med Assoc J (2015) 17(1):5–10. 25739168

[180] Maizels RM Withers DR. MHC-II: a mutual support system for ILCs and T cells? Immunity (2014) 41(2):174–6. 10.1016/j.immuni.2014.07.006 25148019

[181] von Burg N Chappaz S Baerenwaldt A Horvath E Bose Dasgupta S Ashok D Activated group 3 innate lymphoid cells promote T-cell-mediated immune responses. Proc Natl Acad Sci U S A (2014) 111(35):12835–40. 10.1073/pnas.1406908111 25136120 PMC4156721

[182] Spits H Artis D Colonna M Diefenbach A Di Santo JP Eberl G Innate lymphoid cells – a proposal for uniform nomenclature. Nat Rev Immunol (2013) 13(2):145–9. 10.1038/nri3365 23348417

[183] Oliphant CJ Hwang YY Walker JA Salimi M Wong SH Brewer JM MHCII-mediated dialog between group 2 innate lymphoid cells and CD4(+) T cells potentiates type 2 immunity and promotes parasitic helminth expulsion. Immunity (2014) 41(2):283–95. 10.1016/j.immuni.2014.06.016 25088770 PMC4148706

[184] Sonnenberg GF Artis D. Innate lymphoid cell interactions with microbiota: implications for intestinal health and disease. Immunity (2012) 37(4):601–10. 10.1016/j.immuni.2012.10.003 23084357 PMC3495160

[185] Mbongue J Nicholas D Firek A Langridge W. The role of dendritic cells in tissue-specific autoimmunity. J Immunol Res (2014) 2014:857143. 10.1155/2014/857143 24877157 PMC4022068

[186] Yamane H Paul WE. Early signaling events that underlie fate decisions of naive CD4(+) T cells toward distinct T-helper cell subsets. Immunol Rev (2013) 252(1):12–23. 10.1111/imr.12032 23405892 PMC3578301

[187] Miniati I Guiducci S Conforti ML Rogai V Fiori G Cinelli M Autologous stem cell transplantation improves microcirculation in systemic sclerosis. Ann Rheum Dis (2009) 68(1):94–8. 10.1136/ard.2007.082495 18308744

[188] Fleming JN Nash RA McLeod DO Fiorentino DF Shulman HM Connolly MK Capillary regeneration in scleroderma: stem cell therapy reverses phenotype? PLoS One (2008) 3(1):e1452. 10.1371/journal.pone.0001452 18197262 PMC2175530

[189] Postlethwaite AE. Role of T cells and cytokines in effecting fibrosis. Int Rev Immunol (1995) 12(2–4):247–58. 10.3109/08830189509056716 7650423

[190] Parel Y Aurrand-Lions M Scheja A Dayer JM Roosnek E Chizzolini C. Presence of CD4+CD8+ double-positive T cells with very high interleukin-4 production potential in lesional skin of patients with systemic sclerosis. Arthritis Rheum (2007) 56(10):3459–67. 10.1002/art.22927 17907151

[191] Shibuya A Campbell D Hannum C Yssel H Franz-Bacon K McClanahan T DNAM-1, a novel adhesion molecule involved in the cytolytic function of T lymphocytes. Immunity (1996) 4(6):573–81. 10.1016/S1074-7613(00)70060-4 8673704

[192] Giacomelli R Matucci-Cerinic M Cipriani P Ghersetich I Lattanzio R Pavan A Circulating Vdelta1+ T cells are activated and accumulate in the skin of systemic sclerosis patients. Arthritis Rheum (1998) 41(2):327–34. 10.1002/1529-0131(199802)41:2<327::AID-ART17>3.3.CO;2-J 9485091

[193] Sakkas LI Xu B Artlett CM Lu S Jimenez SA Platsoucas CD. Oligoclonal T cell expansion in the skin of patients with systemic sclerosis. J Immunol (2002) 168(7):3649–59. 10.4049/jimmunol.168.7.3649 11907131

[194] Stuart JM Postlethwaite AE Kang AH. Evidence for cell-mediated immunity to collagen in progressive systemic sclerosis. J Lab Clin Med (1976) 88(4):601–7. 965811

[195] Warrington KJ Nair U Carbone LD Kang AH Postlethwaite AE. Characterisation of the immune response to type I collagen in scleroderma. Arthritis Res Ther (2006) 8(4):R136. 10.1186/ar2025 16879746 PMC1779396

[196] Hu PQ Oppenheim JJ Medsger TA Jr Wright TM. T cell lines from systemic sclerosis patients and healthy controls recognize multiple epitopes on DNA topoisomerase I. J Autoimmun (2006) 26(4):258–67. 10.1016/j.jaut.2006.03.004 16735104

[197] Daskalova M Taskov H Dimitrova E Baydanoff S. Humoral and cellular immune response to elastin in patients with systemic sclerosis. Autoimmunity (1997) 25(4):233–41. 10.3109/08916939708994732 9344331

[198] Huffstutter JE DeLustro FA LeRoy EC. Cellular immunity to collagen and laminin in scleroderma. Arthritis Rheum (1985) 28(7):775–80. 10.1002/art.1780280708 4015724

[199] McKown KM Carbone LD Bustillo J Seyer JM Kang AH Postlethwaite AE. Induction of immune tolerance to human type I collagen in patients with systemic sclerosis by oral administration of bovine type I collagen. Arthritis Rheum (2000) 43(5):1054–61. 10.1002/1529-0131(200005)43:5<1054::AID-ANR14>3.0.CO;2-W 10817559

[200] Carbone LD McKown K Pugazhenthi M Barrow KD Warrington K Somes G Dosage effects of orally administered bovine type I collagen on immune function in patients with systemic sclerosis. Arthritis Rheum (2004) 50(8):2713–5. 10.1002/art.20361 15334493

[201] Postlethwaite AE Wong WK Clements P Chatterjee S Fessler BJ Kang AH A multicenter, randomized, double-blind, placebo-controlled trial of oral type I collagen treatment in patients with diffuse cutaneous systemic sclerosis: I. oral type I collagen does not improve skin in all patients, but may improve skin in late-phase disease. Arthritis Rheum (2008) 58(6):1810–22. 10.1002/art.23501 18512816 PMC4511098

[202] Nelson JL Microchimerism and scleroderma. Curr Rheumatol Rep (1999) 1(1):15–21. 10.1007/s11926-999-0019-z 11123009

[203] Giacomelli R Cipriani P Fulminis A Nelson JL Matucci-Cerinic M. Gamma/delta T cells in placenta and skin: their different functions may support the paradigm of microchimerism in systemic sclerosis. Clin Exp Rheumatol (2004) 22(3 Suppl 33):S28–30. 15344594

[204] Adams Waldorf KM Nelson JL. Autoimmune disease during pregnancy and the microchimerism legacy of pregnancy. Immunol Invest (2008) 37(5):631–44. 10.1080/08820130802205886 18716941 PMC2709983

[205] Scaletti C Vultaggio A Bonifacio S Emmi L Torricelli F Maggi E Th2-oriented profile of male offspring T cells present in women with systemic sclerosis and reactive with maternal major histocompatibility complex antigens. Arthritis Rheum (2002) 46(2):445–50. 10.1002/art.10049 11840447

[206] Radstake TR van Bon L Broen J Hussiani A Hesselstrand R Wuttge DM The pronounced Th17 profile in systemic sclerosis (SSc) together with intracellular expression of TGFbeta and IFNgamma distinguishes SSc phenotypes. PLoS One (2009) 4(6):e5903. 10.1371/journal.pone.0005903 19536281 PMC2691991

[207] Papp G Horvath IF Barath S Gyimesi E Sipka S Szodoray P Altered T-cell and regulatory cell repertoire in patients with diffuse cutaneous systemic sclerosis. Scand J Rheumatol (2011) 40(3):205–10. 10.3109/03009742.2010.528021 21366383

[208] Klein S Kretz CC Ruland V Stumpf C Haust M Hartschuh W Reduction of regulatory T cells in skin lesions but not in peripheral blood of patients with systemic scleroderma. Ann Rheum Dis (2011) 70(8):1475–81. 10.1136/ard.2009.116525 21097800

[209] Slobodin G Ahmad MS Rosner I Peri R Rozenbaum M Kessel A Regulatory T cells (CD4(+)CD25(bright)FoxP3(+)) expansion in systemic sclerosis correlates with disease activity and severity. Cell Immunol (2010) 261(2):77–80. 10.1016/j.cellimm.2009.12.009 20096404

[210] Fenoglio D Battaglia F Parodi A Stringara S Negrini S Panico N Alteration of Th17 and Treg cell subpopulations co-exist in patients affected with systemic sclerosis. Clin Immunol (2011) 139(3):249–57. 10.1016/j.clim.2011.01.013 21419712

[211] Mathian A Parizot C Dorgham K Trad S Arnaud L Larsen M Activated and resting regulatory T cell exhaustion concurs with high levels of interleukin-22 expression in systemic sclerosis lesions. Ann Rheum Dis (2012) 71(7):1227–34. 10.1136/annrheumdis-2011-200709 22696687

[212] Liu G Burns S Huang G Boyd K Proia RL Flavell RA The receptor S1P1 overrides regulatory T cell-mediated immune suppression through Akt-mTOR. Nat Immunol (2009) 10(7):769–77. 10.1038/ni.1743 19483717 PMC2732340

[213] Piconese S Gri G Tripodo C Musio S Gorzanelli A Frossi B Mast cells counteract regulatory T-cell suppression through interleukin-6 and OX40/OX40L axis toward Th17-cell differentiation. Blood (2009) 114(13):2639–48. 10.1182/blood-2009-05-220004 19643985

[214] Tokumura A Carbone LD Yoshioka Y Morishige J Kikuchi M Postlethwaite A Elevated serum levels of arachidonoyl-lysophosphatidic acid and sphingosine 1-phosphate in systemic sclerosis. Int J Med Sci (2009) 6(4):168–76. 10.7150/ijms.6.168 19521548 PMC2695151

[215] Liao JJ Huang MC Goetzl EJ. Cutting edge: alternative signaling of Th17 cell development by sphingosine 1-phosphate. J Immunol (2007) 178(9):5425–8. 10.4049/jimmunol.178.9.5425 17442922

[216] Arnson Y Amital H Agmon-Levin N Alon D Sanchez-Castanon M Lopez-Hoyos M Serum 25-OH vitamin D concentrations are linked with various clinical aspects in patients with systemic sclerosis: a retrospective cohort study and review of the literature. Autoimmun Rev (2011) 10(8):490–4. 10.1016/j.autrev.2011.02.002 21320645

[217] Caramaschi P Dalla Gassa A Ruzzenente O Volpe A Ravagnani V Tinazzi I Very low levels of vitamin D in systemic sclerosis patients. Clin Rheumatol (2010) 29(12):1419–25. 10.1007/s10067-010-1478-3 20454816

[218] Vacca A Cormier C Piras M Mathieu A Kahan A Allanore Y. Vitamin D deficiency and insufficiency in 2 independent cohorts of patients with systemic sclerosis. J Rheumatol (2009) 36(9):1924–9. 10.3899/jrheum.081287 19648299

[219] Szodoray P Nakken B Gaal J Jonsson R Szegedi A Zold E The complex role of vitamin D in autoimmune diseases. Scand J Immunol (2008) 68(3):261–9. 10.1111/j.1365-3083.2008.02127.x 18510590

[220] Rolf L Muris AH Hupperts R Damoiseaux J. Vitamin D effects on B cell function in autoimmunity. Ann N Y Acad Sci (2014) 1317:84–91. 10.1111/nyas.12440 24761763

[221] Drozdenko G Heine G Worm M. Oral vitamin D increases the frequencies of CD38+ human B cells and ameliorates IL-17-producing T cells. Exp Dermatol (2014) 23(2):107–12. 10.1111/exd.12300 24313624

[222] Terrier B Derian N Schoindre Y Chaara W Geri G Zahr N Restoration of regulatory and effector T cell balance and B cell homeostasis in systemic lupus erythematosus patients through vitamin D supplementation. Arthritis Res Ther (2012) 14(5):R221. 10.1186/ar4060 23075451 PMC3580532

[223] Pattanaik D Postlethwaite AE A role for lysophosphatidic acid and sphingosine 1-phosphate in the pathogenesis of systemic sclerosis. Discov Med (2010) 10(51):161–7. 20807477

[224] Gendaszewska-Darmach E. Lysophosphatidic acids, cyclic phosphatidic acids and autotaxin as promising targets in therapies of cancer and other diseases. Acta Biochim Pol (2008) 55(2):227–40. 18560605

[225] Rivera J Proia RL Olivera A. The alliance of sphingosine-1-phosphate and its receptors in immunity. Nat Rev Immunol (2008) 8(10):753–63. 10.1038/nri2400 18787560 PMC2600775

[226] Sensken SC Bode C Nagarajan M Peest U Pabst O Graler MH. Redistribution of sphingosine 1-phosphate by sphingosine kinase 2 contributes to lymphopenia. J Immunol (2010) 184(8):4133–42. 10.4049/jimmunol.0903358 20220090

[227] Hughes P Holt S Rowell NR Dodd J. Thymus-dependent (T) lymphocyte deficiency in progressive systemic sclerosis. Br J Dermatol (1976) 95(5):469–73. 10.1111/j.1365-2133.1976.tb00855.x 1086678

[228] Leslie DS Dascher CC Cembrola K Townes MA Hava DL Hugendubler LC Serum lipids regulate dendritic cell CD1 expression and function. Immunology (2008) 125(3):289–301. 10.1111/j.1365-2567.2008.02842.x 18445008 PMC2669133

[229] D’Angelo WA Fries JF Masi AT Shulman LE Pathologic observations in systemic sclerosis (scleroderma). A study of fifty-eight autopsy cases and fifty-eight matched controls. Am J Med (1969) 46(3):428–40. 10.1016/0002-9343(69)90044-8 5780367

[230] Cool CD Kennedy D Voelkel NF Tuder RM. Pathogenesis and evolution of plexiform lesions in pulmonary hypertension associated with scleroderma and human immunodeficiency virus infection. Hum Pathol (1997) 28(4):434–42. 10.1016/S0046-8177(97)90032-0 9104943

[231] Dorfmuller P Humbert M Perros F Sanchez O Simonneau G Muller KM Fibrous remodeling of the pulmonary venous system in pulmonary arterial hypertension associated with connective tissue diseases. Hum Pathol (2007) 38(6):893–902. 10.1016/j.humpath.2006.11.022 17376507

[232] Nagai Y Yamanaka M Hashimoto C Nakano A Hasegawa A Tanaka Y Autopsy case of systemic sclerosis with severe pulmonary hypertension. J Dermatol (2007) 34(11):769–72. 10.1111/j.1346-8138.2007.00381.x 17973818

[233] Cannon PJ Hassar M Case DB Casarella WJ Sommers SC LeRoy EC The relationship of hypertension and renal failure in scleroderma (progressive systemic sclerosis) to structural and functional abnormalities of the renal cortical circulation. Medicine (1974) 53(1):1–46. 10.1097/00005792-197401000-00001 4808710

[234] Kahaleh B. Vascular disease in scleroderma: mechanisms of vascular injury. Rheum Dis Clin North Am (2008) 34(1):57–71. 10.1016/j.rdc.2007.12.004 18329532

[235] Prescott RJ Freemont AJ Jones CJ Hoyland J Fielding P. Sequential dermal microvascular and perivascular changes in the development of scleroderma. J Pathol (1992) 166(3):255–63. 10.1002/path.1711660307 1517881

[236] Fleischmajer R Perlish JS Shaw KV Pirozzi DJ. Skin capillary changes in early systemic scleroderma. Electron microscopy and “in vitro” autoradiography with tritiated thymidine. Arch Dermatol (1976) 112(11):1553–7. 10.1001/archderm.112.11.1553 984860

[237] Freemont AJ Jones CJ Bromley M Andrews P. Changes in vascular endothelium related to lymphocyte collections in diseased synovia. Arthritis Rheum (1983) 26(12):1427–33. 10.1002/art.1780261203 6651893

[238] Grassi W Medico PD Izzo F Cervini C. Microvascular involvement in systemic sclerosis: capillaroscopic findings. Semin Arthritis Rheum (2001) 30(6):397–402. 10.1053/sarh.2001.20269 11404822

[239] Norton WL Nardo JM Vascular disease in progressive systemic sclerosis (scleroderma). Ann Intern Med (1970) 73(2):317–24. 10.7326/0003-4819-73-2-317 4916764

[240] Fleischmajer R Perlish JS Capillary alterations in scleroderma. J Am Acad Dermatol (1980) 2(2):161–70. 10.1016/S0190-9622(80)80396-3 7364973

[241] Herrick AL Pathogenesis of Raynaud’s phenomenon. Rheumatology (2005) 44(5):587–96. 10.1093/rheumatology/keh552 15741200

[242] Kahaleh B Meyer O Scorza R Assessment of vascular involvement. Clin Exp Rheumatol (2003) 21(3 Suppl 29):S9–14. 12889215

[243] Kahaleh B. The microvascular endothelium in scleroderma. Rheumatology (2008) 47(Suppl 5):v14–5. 10.1093/rheumatology/ken279 18784128

[244] Lunardi C Bason C Navone R Millo E Damonte G Corrocher R Systemic sclerosis immunoglobulin G autoantibodies bind the human cytomegalovirus late protein UL94 and induce apoptosis in human endothelial cells. Nat Med (2000) 6(10):1183–6. 10.1038/80533 11017152

[245] Ahmed SS Tan FK Arnett FC Jin L Geng YJ. Induction of apoptosis and fibrillin 1 expression in human dermal endothelial cells by scleroderma sera containing anti-endothelial cell antibodies. Arthritis Rheum (2006) 54(7):2250–62. 10.1002/art.21952 16802364

[246] Yoshizaki A Yanaba K Ogawa A Iwata Y Ogawa F Takenaka M The specific free radical scavenger edaravone suppresses fibrosis in the bleomycin-induced and tight skin mouse models of systemic sclerosis. Arthritis Rheum (2011) 63(10):3086–97. 10.1002/art.30470 21618208

[247] Manetti M Guiducci S Romano E Rosa I Ceccarelli C Mello T Differential expression of junctional adhesion molecules in different stages of systemic sclerosis. Arthritis Rheum (2013) 65(1):247–57. 10.1002/art.37712 23001478

[248] Blann AD Illingworth K Jayson MI. Mechanisms of endothelial cell damage in systemic sclerosis and Raynaud’s phenomenon. J Rheumatol (1993) 20(8):1325–30. 8230013

[249] Schachna L Wigley FM. Targeting mediators of vascular injury in scleroderma. Curr Opin Rheumatol (2002) 14(6):686–93. 10.1097/00002281-200211000-00010 12410092

[250] Kuwana M Okazaki Y. Quantification of circulating endothelial progenitor cells in systemic sclerosis: a direct comparison of protocols. Ann Rheum Dis (2012) 71(4):617–20. 10.1136/annrheumdis-2011-200713 22258488

[251] Del Papa N Colombo G Fracchiolla N Moronetti LM Ingegnoli F Maglione W Circulating endothelial cells as a marker of ongoing vascular disease in systemic sclerosis. Arthritis Rheum (2004) 50(4):1296–304. 10.1002/art.20116 15077314

[252] Sgonc R Gruschwitz MS Dietrich H Recheis H Gershwin ME Wick G. Endothelial cell apoptosis is a primary pathogenetic event underlying skin lesions in avian and human scleroderma. J Clin Invest (1996) 98(3):785–92. 10.1172/JCI118851 8698871 PMC507489

[253] Albert ML Jegathesan M Darnell RB. Dendritic cell maturation is required for the cross-tolerization of CD8+ T cells. Nat Immunol (2001) 2(11):1010–7. 10.1038/ni722 11590405

[254] Greeno EW Bach RR Moldow CF. Apoptosis is associated with increased cell surface tissue factor procoagulant activity. Lab Invest (1996) 75(2):281–9. 8765328

[255] Tsuji S Kaji K Nagasawa S. Activation of the alternative pathway of human complement by apoptotic human umbilical vein endothelial cells. J Biochem (1994) 116(4):794–800. 7883753 10.1093/oxfordjournals.jbchem.a124598

[256] Fleming KE Wanless IR. Glutamine synthetase expression in activated hepatocyte progenitor cells and loss of hepatocellular expression in congestion and cirrhosis. Liver Int (2013) 33(4):525–34. 10.1111/liv.12099 23362937

[257] Lenna S Farina AG Martyanov V Christmann RB Wood TA Farber HW Increased expression of endoplasmic reticulum stress and unfolded protein response genes in peripheral blood mononuclear cells from patients with limited cutaneous systemic sclerosis and pulmonary arterial hypertension. Arthritis Rheum (2013) 65(5):1357–66. 10.1002/art.37891 23400395 PMC3636187

[258] Gargalovic PS Gharavi NM Clark MJ Pagnon J Yang WP He A The unfolded protein response is an important regulator of inflammatory genes in endothelial cells. Arterioscler Thromb Vasc Biol (2006) 26(11):2490–6. 10.1161/01.ATV.0000242903.41158.a1 16931790

[259] Distler O Del Rosso A Giacomelli R Cipriani P Conforti ML Guiducci S Angiogenic and angiostatic factors in systemic sclerosis: increased levels of vascular endothelial growth factor are a feature of the earliest disease stages and are associated with the absence of fingertip ulcers. Arthritis Res (2002) 4(6):R11. 10.1186/ar547 12453314 PMC153841

[260] Distler O Distler JH Scheid A Acker T Hirth A Rethage J Uncontrolled expression of vascular endothelial growth factor and its receptors leads to insufficient skin angiogenesis in patients with systemic sclerosis. Circ Res (2004) 95(1):109–16. 10.1161/01.RES.0000134644.89917.96 15178641

[261] Hummers LK. Microvascular damage in systemic sclerosis: detection and monitoring with biomarkers. Curr Rheumatol Rep (2006) 8(2):131–7. 10.1007/s11926-006-0053-z 16569372

[262] Davies CA Jeziorska M Freemont AJ Herrick AL. The differential expression of VEGF, VEGFR-2, and GLUT-1 proteins in disease subtypes of systemic sclerosis. Hum Pathol (2006) 37(2):190–7. 10.1016/j.humpath.2005.10.007 16426919

[263] Mackiewicz Z Sukura A Povilenaité D Ceponis A Virtanen I Hukkanen M Increased but imbalanced expression of VEGF and its receptors has no positive effect on angiogenesis in systemic sclerosis skin. Clin Exp Rheumatol (2002) 20(5):641–6. 12412194

[264] Koch AE Kronfeld-Harrington LB Szekanecz Z Cho MM Haines GK Harlow LA In situ expression of cytokines and cellular adhesion molecules in the skin of patients with systemic sclerosis. Their role in early and late disease. Pathobiology (1993) 61(5–6):239–46. 10.1159/000163802 7507681

[265] Mulligan-Kehoe MJ Drinane MC Mollmark J Casciola-Rosen L Hummers LK Hall A Antiangiogenic plasma activity in patients with systemic sclerosis. Arthritis Rheum (2007) 56(10):3448–58. 10.1002/art.22861 17907150

[266] Distler JH Allanore Y Avouac J Giacomelli R Guiducci S Moritz F EULAR scleroderma trials and research group statement and recommendations on endothelial precursor cells. Ann Rheum Dis (2009) 68(2):163–8. 10.1136/ard.2008.091918 18653485

[267] Avouac J Juin F Wipff J Couraud PO Chiocchia G Kahan A Circulating endothelial progenitor cells in systemic sclerosis: association with disease severity. Ann Rheum Dis (2008) 67(10):1455–60. 10.1136/ard.2007.082131 18174219

[268] Kuwana M Okazaki Y Yasuoka H Kawakami Y Ikeda Y Defective vasculogenesis in systemic sclerosis. Lancet (2004) 364(9434):603–10. 10.1016/S0140-6736(04)16853-0 15313361

[269] Kuwana M Kaburaki J Okazaki Y Yasuoka H Kawakami Y Ikeda Y Increase in circulating endothelial precursors by atorvastatin in patients with systemic sclerosis. Arthritis Rheum (2006) 54(6):1946–51. 10.1002/art.21899 16729283

[270] Zhu S Evans S Yan B Povsic TJ Tapson V Goldschmidt-Clermont PJ Transcriptional regulation of Bim by FOXO3a and Akt mediates scleroderma serum-induced apoptosis in endothelial progenitor cells. Circulation (2008) 118(21):2156–65. 10.1161/CIRCULATIONAHA.108.787200 18981303 PMC3719010

[271] Cipriani P Guiducci S Miniati I Cinelli M Urbani S Marrelli A Impairment of endothelial cell differentiation from bone marrow-derived mesenchymal stem cells: new insight into the pathogenesis of systemic sclerosis. Arthritis Rheum (2007) 56(6):1994–2004. 10.1002/art.22698 17530639

[272] Jain RK Booth MF. What brings pericytes to tumor vessels? J Clin Invest (2003) 112(8):1134–6. 10.1172/JCI20087 14561696 PMC213497

[273] Hirschi KK D’Amore PA Pericytes in the microvasculature. Cardiovasc Res (1996) 32(4):687–98. 10.1016/S0008-6363(96)00063-6 8915187

[274] Sundberg C Ivarsson M Gerdin B Rubin K. Pericytes as collagen-producing cells in excessive dermal scarring. Lab Invest (1996) 74(2):452–66. 8780163

[275] Rajkumar VS Howell K Csiszar K Denton CP Black CM Abraham DJ. Shared expression of phenotypic markers in systemic sclerosis indicates a convergence of pericytes and fibroblasts to a myofibroblast lineage in fibrosis. Arthritis Res Ther (2005) 7(5):R1113–23. 10.1186/ar1790 16207328 PMC1257439

[276] Rajkumar VS Sundberg C Abraham DJ Rubin K Black CM. Activation of microvascular pericytes in autoimmune Raynaud’s phenomenon and systemic sclerosis. Arthritis Rheum (1999) 42(5):930–41. 10.1002/1529-0131(199905)42:5<930::AID-ANR11>3.0.CO;2-1 10323448

[277] Fleming JN Schwartz SM. The pathology of scleroderma vascular disease. Rheum Dis Clin North Am (2008) 34(1):41–55. 10.1016/j.rdc.2008.01.001 18329531

[278] Manzur M Ganss R. Regulator of G protein signaling 5: a new player in vascular remodeling. Trends Cardiovasc Med (2009) 19(1):26–30. 10.1016/j.tcm.2009.04.002 19467451

[279] Helmbold P Fiedler E Fischer M Marsch W. Hyperplasia of dermal microvascular pericytes in scleroderma. J Cutan Pathol (2004) 31(6):431–40. 10.1111/j.0303-6987.2004.00203.x 15186431

[280] Piera-Velazquez S Jimenez SA. Molecular mechanisms of endothelial to mesenchymal cell transition (EndoMT) in experimentally induced fibrotic diseases. Fibrogenesis Tissue Repair (2012) 5(Suppl 1):S7. Proceedings of Fibroproliferative disorders: from biochemical analysis to targeted therapies Petro E Petrides and David Brenner. 10.1186/1755-1536-5-S1-S7 23259736 PMC3368755

[281] Goumans MJ Liu Z ten Dijke P TGF-beta signaling in vascular biology and dysfunction. Cell Res (2009) 19(1):116–27. 10.1038/cr.2008.326 19114994

[282] Medici D Potenta S Kalluri R. Transforming growth factor-beta2 promotes Snail-mediated endothelial-mesenchymal transition through convergence of Smad-dependent and Smad-independent signalling. Biochem J (2011) 437(3):515–20. 10.1042/BJ20101500 21585337 PMC4457510

[283] van Meeteren LA ten Dijke P. Regulation of endothelial cell plasticity by TGF-beta. Cell Tissue Res (2012) 347(1):177–86. 10.1007/s00441-011-1222-6 21866313 PMC3250609

[284] Zeisberg EM Tarnavski O Zeisberg M Dorfman AL McMullen JR Gustafsson E Endothelial-to-mesenchymal transition contributes to cardiac fibrosis. Nat Med (2007) 13(8):952–61. 10.1038/nm1613 17660828

[285] Li Z Jimenez SA. Protein kinase Cdelta and c-Abl kinase are required for transforming growth factor beta induction of endothelial-mesenchymal transition in vitro. Arthritis Rheum (2011) 63(8):2473–83. 10.1002/art.30317 21425122 PMC3134600

[286] Trojanowska M Role of PDGF in fibrotic diseases and systemic sclerosis. Rheumatology (2008) 47(Suppl 5):v2–4. 10.1093/rheumatology/ken265 18784131

[287] Bielecki M Kowal K Lapinska A Chwiesko-Minarowska S Chyczewski L Kowal-Bielecka O. Peripheral blood mononuclear cells from patients with systemic sclerosis spontaneously secrete increased amounts of vascular endothelial growth factor (VEGF) already in the early stage of the disease. Adv Med Sci (2011) 56(2):255–63. 10.2478/v10039-011-0025-z 21983449

[288] Krein PM Winston BW. Roles for insulin-like growth factor I and transforming growth factor-beta in fibrotic lung disease. Chest (2002) 122(6 Suppl):289S–93S. 10.1378/chest.122.6_suppl.289S 12475802

[289] Serrati S Chilla A Laurenzana A Margheri F Giannoni E Magnelli L Systemic sclerosis endothelial cells recruit and activate dermal fibroblasts by induction of a connective tissue growth factor (CCN2)/transforming growth factor beta-dependent mesenchymal-to-mesenchymal transition. Arthritis Rheum (2013) 65(1):258–69. 10.1002/art.37705 22972461

[290] Widyantoro B Emoto N Nakayama K Anggrahini DW Adiarto S Iwasa N Endothelial cell-derived endothelin-1 promotes cardiac fibrosis in diabetic hearts through stimulation of endothelial-to-mesenchymal transition. Circulation (2010) 121(22):2407–18. 10.1161/CIRCULATIONAHA.110.938217 20497976

[291] Ghosh AK Nagpal V Covington JW Michaels MA Vaughan DE. Molecular basis of cardiac endothelial-to-mesenchymal transition (EndMT): differential expression of microRNAs during EndMT. Cell Signal (2012) 24(5):1031–6. 10.1016/j.cellsig.2011.12.024 22245495 PMC3298765

[292] Kumarswamy R Volkmann I Jazbutyte V Dangwal S Park DH Thum T. Transforming growth factor-beta-induced endothelial-to-mesenchymal transition is partly mediated by microRNA-21. Arterioscler Thromb Vasc Biol (2012) 32(2):361–9. 10.1161/ATVBAHA.111.234286 22095988

[293] Yamane K Kashiwagi H Suzuki N Miyauchi T Yanagisawa M Goto K Elevated plasma levels of endothelin-1 in systemic sclerosis. Arthritis Rheum (1991) 34(2):243–4. 10.1002/art.1780340220 1994925

[294] Vancheeswaran R Magoulas T Efrat G Wheeler-Jones C Olsen I Penny R Circulating endothelin-1 levels in systemic sclerosis subsets – a marker of fibrosis or vascular dysfunction? J Rheumatol (1994) 21(10):1838–44. 7837147

[295] Tomita M Fan P Santoro T Kahaleh B Impaired response to mechanical fluid shear-stress (MFSS) by scleroderma (SSC) microvascular endothelial-cells (MVEC) from involved and uninvolved skin. Arthritis Rheum (1997) 40(9 Suppl):1599.

[296] Anderson ME Moore TL Hollis S Clark S Jayson MI Herrick AL Endothelial-dependent vasodilation is impaired in patients with systemic sclerosis, as assessed by low dose iontophoresis. Clin Exp Rheumatol (2003) 21(3):403. 12846066

[297] Berk BC Abe JI Min W Surapisitchat J Yan C. Endothelial atheroprotective and anti-inflammatory mechanisms. Ann N Y Acad Sci (2001) 947:93–109. 10.1111/j.1749-6632.2001.tb03932.x 11795313

[298] Cerinic MM Valentini G Sorano GG D’Angelo S Cuomo G Fenu L Blood coagulation, fibrinolysis, and markers of endothelial dysfunction in systemic sclerosis. Semin Arthritis Rheum (2003) 32(5):285–95. 10.1053/sarh.2002.50011 12701039

[299] Kahaleh MB Osborn I LeRoy EC. Increased factor VIII/von Willebrand factor antigen and von Willebrand factor activity in scleroderma and in Raynaud’s phenomenon. Ann Intern Med (1981) 94(4 Pt 1):482–4. 10.7326/0003-4819-94-4-482 6782927

[300] Greaves M Malia RG Milford Ward A Moult J Holt CM Lindsey N Elevated von Willebrand factor antigen in systemic sclerosis: relationship to visceral disease. Br J Rheumatol (1988) 27(4):281–5. 10.1093/rheumatology/27.4.281 3136816

[301] Herrick AL Illingworth K Blann A Hay CR Hollis S Jayson MI. Von Willebrand factor, thrombomodulin, thromboxane, beta-thromboglobulin and markers of fibrinolysis in primary Raynaud’s phenomenon and systemic sclerosis. Ann Rheum Dis (1996) 55(2):122–7. 10.1136/ard.55.2.122 8712862 PMC1010106

[302] Goodfield MJ Orchard MA Rowell NR. Whole blood platelet aggregation and coagulation factors in patients with systemic sclerosis. Br J Haematol (1993) 84(4):675–80. 10.1111/j.1365-2141.1993.tb03145.x 8217827

[303] Ames PR Lupoli S Alves J Atsumi T Edwards C Iannaccone L The coagulation/fibrinolysis balance in systemic sclerosis: evidence for a haematological stress syndrome. Br J Rheumatol (1997) 36(10):1045–50. 10.1093/rheumatology/36.10.1045 9374919

[304] Hattori N Mizuno S Yoshida Y Chin K Mishima M Sisson TH The plasminogen activation system reduces fibrosis in the lung by a hepatocyte growth factor-dependent mechanism. Am J Pathol (2004) 164(3):1091–8. 10.1016/S0002-9440(10)63196-3 14982862 PMC1614722

[305] Woessner JF Jr Ch. 1: The matrix metalloproteinases family. 1st ed In: Parks WC Meecham RP, editors. Matrix Metalloproteinases. Biology of Extracellular Matrix Series. San Diego, CA: Academic Press (1998). p. 1–14.

[306] Jinnin M Ihn H Yamane K Asano Y Yazawa N Tamaki K. Plasma plasmin-alpha2-plasmin inhibitor complex levels are increased in systemic sclerosis patients with pulmonary hypertension. Rheumatology (2003) 42(2):240–3. 10.1093/rheumatology/keg071 12595617

[307] Kanno Y Kawashita E Kokado A Okada K Ueshima S Matsuo O Alpha2-antiplasmin regulates the development of dermal fibrosis in mice by prostaglandin F(2alpha) synthesis through adipose triglyceride lipase/calcium-independent phospholipase A(2). Arthritis Rheum (2013) 65(2):492–502. 10.1002/art.37767 23124680

[308] Kanno Y Kawashita E Minamida M Kaneiwa A Okada K Ueshima S Alpha2-antiplasmin is associated with the progression of fibrosis. Am J Pathol (2010) 176(1):238–45. 10.2353/ajpath.2010.090150 20008146 PMC2797886

[309] Postlethwaite AE Chiang TM. Platelet contributions to the pathogenesis of systemic sclerosis. Curr Opin Rheumatol (2007) 19(6):574–9. 10.1097/BOR.0b013e3282eeb3a4 17917538

[310] Friedhoff LT Kim E Priddle M Sonenberg M The effect of altered transmembrane ion gradients on membrane potential and aggregation of human platelets in blood plasma. Biochem Biophys Res Commun (1981) 102(3):832–7. 10.1016/0006-291X(81)91613-2 7306189

[311] Chiang TM Beachey EH Kang AH Binding of collagen alpha1 chains to human platelets. J Clin Invest (1977) 59(3):405–11. 10.1172/JCI108653 838857 PMC333375

[312] Igarashi J Michel T. Sphingosine-1-phosphate and modulation of vascular tone. Cardiovasc Res (2009) 82(2):212–20. 10.1093/cvr/cvp064 19233865 PMC2674011

[313] Kawashima T Yamazaki R Matsuzawa Y Yamaura E Takabatake M Otake S Contrary effects of sphingosine-1-phosphate on expression of alpha-smooth muscle actin in transforming growth factor beta1-stimulated lung fibroblasts. Eur J Pharmacol (2012) 696(1–3):120–9. 10.1016/j.ejphar.2012.09.038 23041148

[314] Cremers B Kelsch B Clever YP Hattangadi N Mahnkopf D Speck U Inhibition of neointimal proliferation after bare metal stent implantation with low-pressure drug delivery using a paclitaxel-coated balloon in porcine coronary arteries. Clin Res Cardiol (2012) 101(5):385–91. 10.1007/s00392-011-0408-y 22237489

[315] Zhang C Baker DL Yasuda S Makarova N Balazs L Johnson LR Lysophosphatidic acid induces neointima formation through PPARgamma activation. J Exp Med (2004) 199(6):763–74. 10.1084/jem.20031619 15007093 PMC2212723

[316] Cheng Y Makarova N Tsukahara R Guo H Shuyu E Farrar P Lysophosphatidic acid-induced arterial wall remodeling: requirement of PPARgamma but not LPA1 or LPA2 GPCR. Cell Signal (2009) 21(12):1874–84. 10.1016/j.cellsig.2009.08.003 19709640 PMC2760670

[317] Kandabashi T Shimokawa H Mukai Y Matoba T Kunihiro I Morikawa K Involvement of rho-kinase in agonists-induced contractions of arteriosclerotic human arteries. Arterioscler Thromb Vasc Biol (2002) 22(2):243–8. 10.1161/hq0202.104274 11834523

[318] Chiang TM Postlethwaite AE. A cell model system to study regulation of phosphatidylinositol 3-kinase and protein kinase B activity by cytokines/growth factors produced by type I collagen stimulated immune cells from patients with systemic sclerosis. Biochim Biophys Acta (2007) 1770(8):1181–6. 10.1016/j.bbagen.2007.04.003 17524560 PMC2083118

[319] Asano Y Stawski L Hant F Highland K Silver R Szalai G Endothelial Fli1 deficiency impairs vascular homeostasis: a role in scleroderma vasculopathy. Am J Pathol (2010) 176(4):1983–98. 10.2353/ajpath.2010.090593 20228226 PMC2843486

[320] Del Galdo F Lisanti MP Jimenez SA. Caveolin-1, transforming growth factor-beta receptor internalization, and the pathogenesis of systemic sclerosis. Curr Opin Rheumatol (2008) 20(6):713–9. 10.1097/BOR.0b013e3283103d27 18949888 PMC2732362

[321] Kabouridis PS. Lipid rafts in T cell receptor signalling. Mol Membr Biol (2006) 23(1):49–57. 10.1080/09687860500453673 16611580 PMC2596298

[322] Di Guglielmo GM Le Roy C Goodfellow AF Wrana JL. Distinct endocytic pathways regulate TGF-beta receptor signalling and turnover. Nat Cell Biol (2003) 5(5):410–21. 10.1038/ncb975 12717440

[323] Del Galdo F Sotgia F de Almeida CJ Jasmin JF Musick M Lisanti MP Decreased expression of caveolin 1 in patients with systemic sclerosis: crucial role in the pathogenesis of tissue fibrosis. Arthritis Rheum (2008) 58(9):2854–65. 10.1002/art.23791 18759267 PMC2770094

[324] Wang XM Zhang Y Kim HP Zhou Z Feghali-Bostwick CA Liu F Caveolin-1: a critical regulator of lung fibrosis in idiopathic pulmonary fibrosis. J Exp Med (2006) 203(13):2895–906. 10.1084/jem.20061536 17178917 PMC1850940

[325] Zhao YY Liu Y Stan RV Fan L Gu Y Dalton N Defects in caveolin-1 cause dilated cardiomyopathy and pulmonary hypertension in knockout mice. Proc Natl Acad Sci U S A (2002) 99(17):11375–80. 10.1073/pnas.172360799 12177436 PMC123264

[326] Patel HH Zhang S Murray F Suda RY Head BP Yokoyama U Increased smooth muscle cell expression of caveolin-1 and caveolae contribute to the pathophysiology of idiopathic pulmonary arterial hypertension. FASEB J (2007) 21(11):2970–9. 10.1096/fj.07-8424com 17470567

[327] Manetti M Allanore Y Saad M Fatini C Cohignac V Guiducci S Evidence for caveolin-1 as a new susceptibility gene regulating tissue fibrosis in systemic sclerosis. Ann Rheum Dis (2012) 71(6):1034–41. 10.1136/annrheumdis-2011-200986 22402147

[328] Maurer B Distler JH Distler O The Fra-2 transgenic mouse model of systemic sclerosis. Vascul Pharmacol (2013) 58(3):194–201. 10.1016/j.vph.2012.12.001 23232070

[329] Johnson RL Ziff M Lymphokine stimulation of collagen accumulation. J Clin Invest (1976) 58(1):240–52. 10.1172/JCI108455 932208 PMC333175

[330] Kondo H Rabin BS Rodnan GP. Cutaneous antigen-stimulating lymphokine production by lymphocytes of patients with progressive systemic sclerosis (scleroderma). J Clin Invest (1976) 58(6):1388–94. 10.1172/JCI108594 791970 PMC333310

[331] Postlethwaite AE Snyderman R Kang AH. The chemotactic attraction of human fibroblasts to a lymphocyte-derived factor. J Exp Med (1976) 144(5):1188–203. 10.1084/jem.144.5.1188 825607 PMC2190454

[332] Postlethwaite AE Kang AH. Characterization of guinea pig lymphocyte-derived chemotactic factor for fibroblasts. J Immunol (1980) 124(3):1462–6. 7358987

[333] Postlethwaite AE Kang AH Characterization of fibroblast proliferation factors elaborated by antigen- and mitogen-stimulated guinea pig lymph node cells: differentiation from lymphocyte-derived chemotactic factor for fibroblasts, lymphocyte mitogenic factor, and interleukin 1. Cell Immunol (1982) 73(1):169–78. 10.1016/0008-8749(82)90445-2 6217901

[334] Hibbs MS Postlethwaite AE Mainardi CL Seyer JM Kang AH. Alterations in collagen production in mixed mononuclear leukocyte-fibroblast cultures. J Exp Med (1983) 157(1):47–59. 10.1084/jem.157.1.47 6549655 PMC2186892

[335] Postlethwaite AE Kang AH. Induction of fibroblast proliferation by human mononuclear leukocyte-derived proteins. Arthritis Rheum (1983) 26(1):22–7. 10.1002/art.1780260104 6600611

[336] Postlethwaite AE Smith GN Mainardi CL Seyer JM Kang AH. Lymphocyte modulation of fibroblast function in vitro: stimulation and inhibition of collagen production by different effector molecules. J Immunol (1984) 132(5):2470–7. 6609200

[337] Jimenez SA Rosenbloom J. Production of collagen synthesis inhibitory lymphokine by human leukemic T-lymphocyte cell lines. Immunol Lett (1985) 11(2):69–74. 10.1016/0165-2478(85)90145-2 3878830

[338] Jimenez SA McArthur W Rosenbloom J. Inhibition of collagen synthesis by mononuclear cell supernates. J Exp Med (1979) 150(6):1421–31. 10.1084/jem.150.6.1421 229188 PMC2185728

[339] Neilson EG Jimenez SA Phillips SM Cell-mediated immunity in interstitial nephritis. III. T lymphocyte-mediated fibroblast proliferation and collagen synthesis: an immune mechanism for renal fibrogenesis. J Immunol (1980) 125(4):1708–14. 6967914

[340] Wahl SM Wahl LM McCarthy JB. Lymphocyte-mediated activation of fibroblast proliferation and collagen production. J Immunol (1978) 121(3):942–6. 80435

[341] Roberts AB Sporn MB Assoian RK Smith JM Roche NS Wakefield LM Transforming growth factor type beta: rapid induction of fibrosis and angiogenesis in vivo and stimulation of collagen formation in vitro. Proc Natl Acad Sci U S A (1986) 83(12):4167–71. 10.1073/pnas.83.12.4167 2424019 PMC323692

[342] Varga J Jimenez SA. Stimulation of normal human fibroblast collagen production and processing by transforming growth factor-beta. Biochem Biophys Res Commun (1986) 138(2):974–80. 10.1016/S0006-291X(86)80591-5 3461787

[343] Ignotz RA Massague J. Transforming growth factor-beta stimulates the expression of fibronectin and collagen and their incorporation into the extracellular matrix. J Biol Chem (1986) 261(9):4337–45. 3456347

[344] Raghow R Postlethwaite AE Keski-Oja J Moses HL Kang AH. Transforming growth factor-beta increases steady state levels of type I procollagen and fibronectin messenger RNAs posttranscriptionally in cultured human dermal fibroblasts. J Clin Invest (1987) 79(4):1285–8. 10.1172/JCI112950 3470308 PMC424335

[345] Postlethwaite AE Keski-Oja J Moses HL Kang AH. Stimulation of the chemotactic migration of human fibroblasts by transforming growth factor beta. J Exp Med (1987) 165(1):251–6. 10.1084/jem.165.1.251 3491869 PMC2188256

[346] Worrall JG Whiteside TL Prince RK Buckingham RB Stachura I Rodnan GP. Persistence of scleroderma-like phenotype in normal fibroblasts after prolonged exposure to soluble mediators from mononuclear cells. Arthritis Rheum (1986) 29(1):54–64. 10.1002/art.1780290108 3947417

[347] Tsou PS Balogh B Pinney AJ Zakhem G Lozier A Amin M Lipoic acid plays a role in scleroderma: insights obtained from scleroderma dermal fibroblasts. Arthritis Res Ther (2014) 16(5):411. 10.1186/s13075-014-0411-6 25123250 PMC4558991

[348] Brown M Postlethwaite AE Myers LK Hasty KA. Supernatants from culture of type I collagen-stimulated PBMC from patients with cutaneous systemic sclerosis versus localized scleroderma demonstrate suppression of MMP-1 by fibroblasts. Clin Rheumatol (2012) 31(6):973–81. 10.1007/s10067-012-1962-z 22367096 PMC3362697

[349] Kirk TZ Mark ME Chua CC Chua BH Mayes MD. Myofibroblasts from scleroderma skin synthesize elevated levels of collagen and tissue inhibitor of metalloproteinase (TIMP-1) with two forms of TIMP-1. J Biol Chem (1995) 270(7):3423–8. 10.1074/jbc.270.7.3423 7852429

[350] LeRoy EC. Increased collagen synthesis by scleroderma skin fibroblasts in vitro: a possible defect in the regulation or activation of the scleroderma fibroblast. J Clin Invest (1974) 54(4):880–9. 10.1172/JCI107827 4430718 PMC301627

[351] Serini G Bochaton-Piallat ML Ropraz P Geinoz A Borsi L Zardi L The fibronectin domain ED-A is crucial for myofibroblastic phenotype induction by transforming growth factor-beta1. J Cell Biol (1998) 142(3):873–81. 10.1083/jcb.142.3.873 9700173 PMC2148176

[352] Hinz B Gabbiani G. Cell-matrix and cell-cell contacts of myofibroblasts: role in connective tissue remodeling. Thromb Haemost (2003) 90(6):993–1002. 10.1267/THRO03061002 14652629

[353] Postlethwaite AE Harris LJ Raza SH Kodura S Akhigbe T Pharmacotherapy of systemic sclerosis. Expert Opin Pharmacother (2010) 11(5):789–806. 10.1517/14656561003592177 20210685 PMC2837533

[354] Yin Z Tong Y Zhu H Watsky MA. ClC-3 is required for LPA-activated Cl-current activity and fibroblast-to-myofibroblast differentiation. Am J Physiol Cell Physiol (2008) 294(2):C535–42. 10.1152/ajpcell.00291.2007 18077605

[355] Hamidi S Schafer-Korting M Weindl G. TLR2/1 and sphingosine 1-phosphate modulate inflammation, myofibroblast differentiation and cell migration in fibroblasts. Biochim Biophys Acta (2014) 1841(4):484–94. 10.1016/j.bbalip.2014.01.008 24440818

[356] Maekawa T Jinnin M Ohtsuki M Ihn H. Serum levels of interleukin-1alpha in patients with systemic sclerosis. J Dermatol (2013) 40(2):98–101. 10.1111/1346-8138.12011 23078215

[357] Umehara H Kumagai S Murakami M Suginoshita T Tanaka K Hashida S Enhanced production of interleukin-1 and tumor necrosis factor alpha by cultured peripheral blood monocytes from patients with scleroderma. Arthritis Rheum (1990) 33(6):893–7. 10.1002/art.1780330619 2363741

[358] Postlethwaite AE Raghow R Stricklin GP Poppleton H Seyer JM Kang AH. Modulation of fibroblast functions by interleukin 1: increased steady-state accumulation of type I procollagen messenger RNAs and stimulation of other functions but not chemotaxis by human recombinant interleukin 1 alpha and beta. J Cell Biol (1988) 106(2):311–8. 10.1083/jcb.106.2.311 2828381 PMC2114989

[359] Postlethwaite AE Smith GN Jr Lachman LB Endres RO Poppleton HM Hasty KA Stimulation of glycosaminoglycan synthesis in cultured human dermal fibroblasts by interleukin 1. Induction of hyaluronic acid synthesis by natural and recombinant interleukin 1s and synthetic interleukin 1 beta peptide 163-171. J Clin Invest (1989) 83(2):629–36. 10.1172/JCI113927 2783590 PMC303724

[360] Kirk TZ Mayes MD. IL-1 rescues scleroderma myofibroblasts from serum-starvation-induced cell death. Biochem Biophys Res Commun (1999) 255(1):129–32. 10.1006/bbrc.1999.0155 10082667

[361] Higgins GC Wu Y Postlethwaite AE. Intracellular IL-1 receptor antagonist is elevated in human dermal fibroblasts that overexpress intracellular precursor IL-1 alpha. J Immunol (1999) 163(7):3969–75. 10490999

[362] Kanangat S Postlethwaite AE Higgins GC Hasty KA. Novel functions of intracellular IL-1ra in human dermal fibroblasts: implications in the pathogenesis of fibrosis. J Invest Dermatol (2006) 126(4):756–65. 10.1038/sj.jid.5700097 16456536

[363] Kawaguchi Y McCarthy SA Watkins SC Wright TM. Autocrine activation by interleukin 1alpha induces the fibrogenic phenotype of systemic sclerosis fibroblasts. J Rheumatol (2004) 31(10):1946–54. 15468358

[364] Robertson IB Rifkin DB. Unchaining the beast; insights from structural and evolutionary studies on TGFbeta secretion, sequestration, and activation. Cytokine Growth Factor Rev (2013) 24(4):355–72. 10.1016/j.cytogfr.2013.06.003 23849989 PMC3780968

[365] Lafyatis R. Transforming growth factor beta-at the centre of systemic sclerosis. Nat Rev Rheumatol (2014) 10(12):706–19. 10.1038/nrrheum.2014.137 25136781

[366] Valluru M Staton CA Reed MW Brown NJ. Transforming growth factor-beta and endoglin signaling orchestrate wound healing. Front Physiol (2011) 2:89. 10.3389/fphys.2011.00089 22164144 PMC3230065

[367] Maring JA Trojanowska M ten Dijke P Role of endoglin in fibrosis and scleroderma. Int Rev Cell Mol Biol (2012) 297:295–308. 10.1016/B978-0-12-394308-8.00008-X 22608563 PMC3824608

[368] Buscemi L Ramonet D Klingberg F Formey A Smith-Clerc J Meister JJ The single-molecule mechanics of the latent TGF-beta1 complex. Curr Biol (2011) 21(24):2046–54. 10.1016/j.cub.2011.11.037 22169532

[369] Chizzolini C. Update on pathophysiology of scleroderma with special reference to immunoinflammatory events. Ann Med (2007) 39(1):42–53. 10.1080/07853890601098152 17364450

[370] Lopez-Casillas F Wrana JL Massague J. Betaglycan presents ligand to the TGF beta signaling receptor. Cell (1993) 73(7):1435–44. 10.1016/0092-8674(93)90368-Z 8391934

[371] Mallano T Palumbo-Zerr K Zerr P Ramming A Zeller B Beyer C Activating transcription factor 3 regulates canonical TGFbeta signalling in systemic sclerosis. Ann Rheum Dis (2015). 10.1136/annrheumdis-2014-206214 25589515

[372] O’Reilly S Ciechomska M Cant R van Laar JM. Interleukin-6 (IL-6) trans signaling drives a STAT3-dependent pathway that leads to hyperactive transforming growth factor-beta (TGF-beta) signaling promoting SMAD3 activation and fibrosis via Gremlin protein. J Biol Chem (2014) 289(14):9952–60. 10.1074/jbc.M113.545822 24550394 PMC3975039

[373] Bhattacharyya S Fang F Tourtellotte W Varga J. Egr-1: new conductor for the tissue repair orchestra directs harmony (regeneration) or cacophony (fibrosis). J Pathol (2013) 229(2):286–97. 10.1002/path.4131 23132749 PMC3965177

[374] Chen SJ Ning H Ishida W Sodin-Semrl S Takagawa S Mori Y The early-immediate gene EGR-1 is induced by transforming growth factor-beta and mediates stimulation of collagen gene expression. J Biol Chem (2006) 281(30):21183–97. 10.1074/jbc.M603270200 16702209

[375] Yasuoka H Hsu E Ruiz XD Steinman RA Choi AM Feghali-Bostwick CA. The fibrotic phenotype induced by IGFBP-5 is regulated by MAPK activation and egr-1-dependent and -independent mechanisms. Am J Pathol (2009) 175(2):605–15. 10.2353/ajpath.2009.080991 19628764 PMC2716960

[376] Fang F Shangguan AJ Kelly K Wei J Gruner K Ye B Early growth response 3 (Egr-3) is induced by transforming growth factor-beta and regulates fibrogenic responses. Am J Pathol (2013) 183(4):1197–208. 10.1016/j.ajpath.2013.06.016 23906810 PMC3791870

[377] Postlethwaite AE Raghow R Stricklin G Ballou L Sampath TK. Osteogenic protein-1, a bone morphogenic protein member of the TGF-beta superfamily, shares chemotactic but not fibrogenic properties with TGF-beta. J Cell Physiol (1994) 161(3):562–70. 10.1002/jcp.1041610320 7962137

[378] Xu Y Wan J Jiang D Wu X. BMP-7 counteracts TGF-beta1-induced epithelial-to-mesenchymal transition in human renal proximal tubular epithelial cells. J Nephrol (2009) 22(3):403–10. 19557718

[379] Yamamoto T Eckes B Krieg T. Effect of interleukin-10 on the gene expression of type I collagen, fibronectin, and decorin in human skin fibroblasts: differential regulation by transforming growth factor-beta and monocyte chemoattractant protein-1. Biochem Biophys Res Commun (2001) 281(1):200–5. 10.1006/bbrc.2001.4321 11178980

[380] Hudson LL Rocca KM Kuwana M Pandey JP. Interleukin-10 genotypes are associated with systemic sclerosis and influence disease-associated autoimmune responses. Genes Immun (2005) 6(3):274–8. 10.1038/sj.gene.6364180 15772682

[381] Postlethwaite AE Seyer JM. Stimulation of fibroblast chemotaxis by human recombinant tumor necrosis factor alpha (TNF-alpha) and a synthetic TNF-alpha 31-68 peptide. J Exp Med (1990) 172(6):1749–56. 10.1084/jem.172.6.1749 2258704 PMC2188741

[382] Rosenbloom J Feldman G Freundlich B Jimenez SA. Inhibition of excessive scleroderma fibroblast collagen production by recombinant gamma-interferon. Association with a coordinate decrease in types I and III procollagen messenger RNA levels. Arthritis Rheum (1986) 29(7):851–6. 10.1002/art.1780290706 3091039

[383] Koca SS Isik A Ozercan IH Ustundag B Evren B Metin K. Effectiveness of etanercept in bleomycin-induced experimental scleroderma. Rheumatology (2008) 47(2):172–5. 10.1093/rheumatology/kem344 18174229

[384] Lam GK Hummers LK Woods A Wigley FM Efficacy and safety of etanercept in the treatment of scleroderma-associated joint disease. J Rheumatol (2007) 34(7):1636–7. 17611970

[385] Ilan Y Gotsman I Pines M Beinart R Zeira M Ohana M Induction of oral tolerance in splenocyte recipients toward pretransplant antigens ameliorates chronic graft versus host disease in a murine model. Blood (2000) 95(11):3613–9. 10828052

[386] Pendergrass SA Lemaire R Francis IP Mahoney JM Lafyatis R Whitfield ML. Intrinsic gene expression subsets of diffuse cutaneous systemic sclerosis are stable in serial skin biopsies. J Invest Dermatol (2012) 132(5):1363–73. 10.1038/jid.2011.472 22318389 PMC3326181

[387] Sargent JL Milano A Bhattacharyya S Varga J Connolly MK Chang HY A TGFbeta-responsive gene signature is associated with a subset of diffuse scleroderma with increased disease severity. J Invest Dermatol (2010) 130(3):694–705. 10.1038/jid.2009.318 19812599 PMC3867816

[388] Bhattacharyya S Sargent JL Du P Lin S Tourtellotte WG Takehara K Egr-1 induces a profibrotic injury/repair gene program associated with systemic sclerosis. PLoS One (2011) 6(9):e23082. 10.1371/journal.pone.0023082 21931594 PMC3172216

[389] Greenblatt MB Sargent JL Farina G Tsang K Lafyatis R Glimcher LH Interspecies comparison of human and murine scleroderma reveals IL-13 and CCL2 as disease subset-specific targets. Am J Pathol (2012) 180(3):1080–94. 10.1016/j.ajpath.2011.11.024 22245215 PMC3349888

[390] Johnson ME Mahoney JM Taroni J Sargent JL Marmarelis E Wu MR Experimentally-derived fibroblast gene signatures identify molecular pathways associated with distinct subsets of systemic sclerosis patients in three independent cohorts. PLoS One (2015) 10(1):e0114017. 10.1371/journal.pone.0114017 25607805 PMC4301872

[391] Gabrielli A Avvedimento EV Krieg T Scleroderma. N Engl J Med (2009) 360(19):1989–2003. 10.1056/NEJMra0806188 19420368

[392] Shand L Lunt M Nihtyanova S Hoseini M Silman A Black CM Relationship between change in skin score and disease outcome in diffuse cutaneous systemic sclerosis: application of a latent linear trajectory model. Arthritis Rheum (2007) 56(7):2422–31. 10.1002/art.22721 17599771

[393] Dosquet-Bernard C Wilhelm F Lomri N Tobelem G Caen JP. 1 alpha, 25-dihydroxyvitamin D3 modulates the growth of 3T3 cells and human skin fibroblasts stimulated by platelet-derived growth factor. Cell Biol Int Rep (1986) 10(12):931–8. 10.1016/0309-1651(86)90113-X 3026660

[394] Hyodo H Kimura A Nakata Y Ohta H Kuramoto A. 1 alpha-hydroxyvitamin D3 in the treatment of primary myelofibrosis: in vitro effect of vitamin D3 metabolites on the bone marrow fibroblasts. Int J Hematol (1993) 57(2):131–7. 8388271

[395] Lunghi B Meacci E Stio M Celli A Bruni P Nassi P 1,25-dihydroxyvitamin D3 inhibits proliferation of IMR-90 human fibroblasts and stimulates pyruvate kinase activity in confluent-phase cells. Mol Cell Endocrinol (1995) 115(2):141–8. 10.1016/0303-7207(95)03681-4 8824889

[396] Morimoto S Imanaka S Koh E Shiraishi T Nabata T Kitano S Comparison of the inhibitions of proliferation of normal and psoriatic fibroblasts by 1 alpha,25-dihydroxyvitamin D3 and synthetic analogues of vitamin D3 with an oxygen atom in their side chain. Biochem Int (1989) 19(5):1143–9. 2561444

[397] Wang JC. In vitro effect of 1,25-dihydroxyvitamin D3 on proliferation and collagen synthesis by bone marrow fibroblasts. Acta Haematol (1992) 88(1):27–31. 10.1159/000204591 1414158

[398] Greiling D Thieroff-Ekerdt R. 1alpha,25-dihydroxyvitamin D3 rapidly inhibits fibroblast-induced collagen gel contraction. J Invest Dermatol (1996) 106(6):1236–41. 10.1111/1523-1747.ep12348928 8752663

[399] Srviastava MD DeLuca H Ambrus JL. Inhibition of IL-6 and IL-8 production in human fibroblast cell lines by 1,25 (OH)2 vitamin D3 and two of its analogs with lower calcemic activity. Res Commun Chem Pathol Pharmacol (1994) 83(2):145–50. 8202627

[400] Rostkowska-Nadolska B Sliupkas-Dyrda E Potyka J Kusmierz D Fraczek M Krecicki T Vitamin D derivatives: calcitriol and tacalcitol inhibits interleukin-6 and interleukin-8 expression in human nasal polyp fibroblast cultures. Adv Med Sci (2010) 55(1):86–92. 10.2478/v10039-010-0012-9 20439185

[401] Koli K Keski-Oja J. Vitamin D3 regulation of transforming growth factor-beta system in epithelial and fibroblastic cells – relationships to plasminogen activation. J Investig Dermatol Symp Proc (1996) 1(1):33–8. 9627689

[402] Ramirez AM Wongtrakool C Welch T Steinmeyer A Zugel U Roman J. Vitamin D inhibition of pro-fibrotic effects of transforming growth factor beta1 in lung fibroblasts and epithelial cells. J Steroid Biochem Mol Biol (2010) 118(3):142–50. 10.1016/j.jsbmb.2009.11.004 19931390 PMC2821704

[403] Bottomley WW Jutley J Wood EJ Goodfield MD. The effect of calcipotriol on lesional fibroblasts from patients with active morphea. Acta Derm Venereol (1995) 75(5):364–6. 8615053 10.2340/000155557575364366

[404] Tan X Li Y Liu Y. Therapeutic role and potential mechanisms of active Vitamin D in renal interstitial fibrosis. J Steroid Biochem Mol Biol (2007) 103(3–5):491–6. 10.1016/j.jsbmb.2006.11.011 17207995 PMC2661621

[405] Koshiishi I Mitani H Sumita T Imanari T. 1,25-dihydroxyvitamin D(3) prevents the conversion of adipose tissue into fibrous tissue in skin exposed to chronic UV irradiation. Toxicol Appl Pharmacol (2001) 173(2):99–104. 10.1006/taap.2001.9178 11384211

[406] Matsuoka LY Dannenberg MJ Wortsman J Hollis BW Jimenez SA Varga J. Cutaneous vitamin D3 formation in progressive systemic sclerosis. J Rheumatol (1991) 18(8):1196–8. 1658323

[407] Serup J Hagdrup H. Vitamin D metabolites in generalized scleroderma. Evidence of a normal cutaneous and intestinal supply with vitamin D. Acta Derm Venereol (1985) 65(4):343–5. 2413693

[408] Hulshof MM Bouwes Bavinck JN Bergman W Masclee AA Heickendorff L Breedveld FC Double-blind, placebo-controlled study of oral calcitriol for the treatment of localized and systemic scleroderma. J Am Acad Dermatol (2000) 43(6):1017–23. 10.1067/mjd.2000.108369 11100017

[409] Boelsma E Pavel S Ponec M. Effects of calcitriol on fibroblasts derived from skin of scleroderma patients. Dermatology (1995) 191(3):226–33. 10.1159/000246550 8534941

[410] Zerr P Vollath S Palumbo-Zerr K Tomcik M Huang J Distler A Vitamin D receptor regulates TGF-beta signalling in systemic sclerosis. Ann Rheum Dis (2015) 74(3):e20. 10.1136/annrheumdis-2013-204378 24448349

[411] Artaza JN Norris KC. Vitamin D reduces the expression of collagen and key profibrotic factors by inducing an antifibrotic phenotype in mesenchymal multipotent cells. J Endocrinol (2009) 200(2):207–21. 10.1677/JOE-08-0241 19036760 PMC3787314

[412] Li Y Spataro BC Yang J Dai C Liu Y. 1,25-dihydroxyvitamin D inhibits renal interstitial myofibroblast activation by inducing hepatocyte growth factor expression. Kidney Int (2005) 68(4):1500–10. 10.1111/j.1523-1755.2005.00562.x 16164627

[413] Slominski A Janjetovic Z Tuckey RC Nguyen MN Bhattacharya KG Wang J 20S-hydroxyvitamin D3, noncalcemic product of CYP11A1 action on vitamin D3, exhibits potent antifibrogenic activity in vivo. J Clin Endocrinol Metab (2013) 98(2):E298–303. 10.1210/jc.2012-3074 23295467 PMC3565109

[414] Cerutis DR Dreyer A Cordini F McVaney TP Mattson JS Parrish LC Lysophosphatidic acid modulates the regenerative responses of human gingival fibroblasts and enhances the actions of platelet-derived growth factor. J Periodontol (2004) 75(2):297–305. 10.1902/jop.2004.75.2.297 15068119

[415] Xu MY Porte J Knox AJ Weinreb PH Maher TM Violette SM Lysophosphatidic acid induces alphavbeta6 integrin-mediated TGF-beta activation via the LPA2 receptor and the small G protein G alpha(q). Am J Pathol (2009) 174(4):1264–79. 10.2353/ajpath.2009.080160 19147812 PMC2671359

[416] Fang X Yu S LaPushin R Lu Y Furui T Penn LZ Lysophosphatidic acid prevents apoptosis in fibroblasts via G(i)-protein-mediated activation of mitogen-activated protein kinase. Biochem J (2000) 352(Pt 1):135–43. 10.1042/0264-6021:3520135 11062066 PMC1221440

[417] Yin Z Carbone LD Gotoh M Postlethwaite A Bolen AL Tigyi GJ Lysophosphatidic acid-activated Cl-current activity in human systemic sclerosis skin fibroblasts. Rheumatology (2010) 49(12):2290–7. 10.1093/rheumatology/keq260 20823096 PMC2981513

[418] Castelino FV Seiders J Bain G Brooks SF King CD Swaney JS Amelioration of dermal fibrosis by genetic deletion or pharmacologic antagonism of lysophosphatidic acid receptor 1 in a mouse model of scleroderma. Arthritis Rheum (2011) 63(5):1405–15. 10.1002/art.30262 21305523 PMC3086986

[419] Huang LS Fu P Patel P Harijith A Sun T Zhao Y Lysophosphatidic acid receptor-2 deficiency confers protection against bleomycin-induced lung injury and fibrosis in mice. Am J Respir Cell Mol Biol (2013) 49(6):912–22. 10.1165/rcmb.2013-0070OC 23808384 PMC3931116

[420] Hashimoto M Wang X Mao L Kobayashi T Kawasaki S Mori N Sphingosine 1-phosphate potentiates human lung fibroblast chemotaxis through the S1P2 receptor. Am J Respir Cell Mol Biol (2008) 39(3):356–63. 10.1165/rcmb.2006-0427OC 18367729 PMC2542450

[421] Bu S Asano Y Bujor A Highland K Hant F Trojanowska M. Dihydrosphingosine 1-phosphate has a potent antifibrotic effect in scleroderma fibroblasts via normalization of phosphatase and tensin homolog levels. Arthritis Rheum (2010) 62(7):2117–26. 10.1002/art.27463 20309867 PMC3034368

[422] Xin C Ren S Kleuser B Shabahang S Eberhardt W Radeke H Sphingosine 1-phosphate cross-activates the Smad signaling cascade and mimics transforming growth factor-beta-induced cell responses. J Biol Chem (2004) 279(34):35255–62. 10.1074/jbc.M312091200 15192102

[423] Yamanaka M Shegogue D Pei H Bu S Bielawska A Bielawski J Sphingosine kinase 1 (SPHK1) is induced by transforming growth factor-beta and mediates TIMP-1 up-regulation. J Biol Chem (2004) 279(52):53994–4001. 10.1074/jbc.M410144200 15485866

[424] Huu DL Matsushita T Jin G Hamaguchi Y Hasegawa M Takehara K FTY720 ameliorates murine sclerodermatous chronic graft-versus-host disease by promoting expansion of splenic regulatory cells and inhibiting immune cell infiltration into skin. Arthritis Rheum (2013) 65(6):1624–35. 10.1002/art.37933 23508350

[425] Rouzer CA Marnett LJ Endocannabinoid oxygenation by cyclooxygenases, lipoxygenases, and cytochromes P450: cross-talk between the eicosanoid and endocannabinoid signaling pathways. Chem Rev (2011) 111(10):5899–921. 10.1021/cr2002799 21923193 PMC3191732

[426] Mechoulam R Fride E Di Marzo V. Endocannabinoids. Eur J Pharmacol (1998) 359(1):1–18. 10.1016/S0014-2999(98)00649-9 9831287

[427] Martinez-Pinilla E Reyes-Resina I Onatibia-Astibia A Zamarbide M Ricobaraza A Navarro G CB and GPR55 receptors are co-expressed and form heteromers in rat and monkey striatum. Exp Neurol (2014) 261C:44–52. 10.1016/j.expneurol.2014.06.017 24967683

[428] Balenga NA Martinez-Pinilla E Kargl J Schroder R Peinhaupt M Platzer W Heteromerization of GPR55 and cannabinoid CB receptors modulates signalling. Br J Pharmacol (2014) 171(23):5387–406. 10.1111/bph.12850 25048571 PMC4294047

[429] Penumarti A Abdel-Rahman AA The novel endocannabinoid receptor GPR18 is expressed in the rostral ventrolateral medulla and exerts tonic restraining influence on blood pressure. J Pharmacol Exp Ther (2014) 349(1):29–38. 10.1124/jpet.113.209213 24431468 PMC3965889

[430] MacIntyre J Dong A Straiker A Zhu J Howlett SE Bagher A Cannabinoid and lipid-mediated vasorelaxation in retinal microvasculature. Eur J Pharmacol (2014) 735:105–14. 10.1016/j.ejphar.2014.03.055 24751709

[431] McHugh D Hu SS Rimmerman N Juknat A Vogel Z Walker JM N-arachidonoyl glycine, an abundant endogenous lipid, potently drives directed cellular migration through GPR18, the putative abnormal cannabidiol receptor. BMC Neurosci (2010) 11:44 10.1186/1471-2202-11-44 20346144 PMC2865488

[432] Palumbo-Zerr K Horn A Distler A Zerr P Dees C Beyer C Inactivation of fatty acid amide hydrolase exacerbates experimental fibrosis by enhanced endocannabinoid-mediated activation of CB1. Ann Rheum Dis (2012) 71(12):2051–4. 10.1136/annrheumdis-2012-201823 22915616

[433] Hwang SW Cho H Kwak J Lee SY Kang CJ Jung J Direct activation of capsaicin receptors by products of lipoxygenases: endogenous capsaicin-like substances. Proc Natl Acad Sci U S A (2000) 97(11):6155–60. 10.1073/pnas.97.11.6155 10823958 PMC18574

[434] Szabo A Czirjak L Sandor Z Helyes Z Laszlo T Elekes K Investigation of sensory neurogenic components in a bleomycin-induced scleroderma model using transient receptor potential vanilloid 1 receptor- and calcitonin gene-related peptide-knockout mice. Arthritis Rheum (2008) 58(1):292–301. 10.1002/art.23168 18163477

[435] Kozela E Juknat A Kaushansky N Rimmerman N Ben-Nun A Vogel Z. Cannabinoids decrease the th17 inflammatory autoimmune phenotype. J Neuroimmune Pharmacol (2013) 8(5):1265–76. 10.1007/s11481-013-9493-1 23892791

[436] Kozlowska H Baranowska M Schlicker E Kozlowski M Laudanski J Malinowska B. Virodhamine relaxes the human pulmonary artery through the endothelial cannabinoid receptor and indirectly through a COX product. Br J Pharmacol (2008) 155(7):1034–42. 10.1038/bjp.2008.371 18806815 PMC2597267

[437] Rajesh M Mukhopadhyay P Batkai S Hasko G Liaudet L Huffman JW CB2-receptor stimulation attenuates TNF-alpha-induced human endothelial cell activation, transendothelial migration of monocytes, and monocyte-endothelial adhesion. Am J Physiol Heart Circ Physiol (2007) 293(4):H2210–8. 10.1152/ajpheart.00688.2007 17660390 PMC2229632

[438] Rajesh M Mukhopadhyay P Hasko G Pacher P. Cannabinoid CB1 receptor inhibition decreases vascular smooth muscle migration and proliferation. Biochem Biophys Res Commun (2008) 377(4):1248–52. 10.1016/j.bbrc.2008.10.159 18996082 PMC2646252

[439] Garcia-Gonzalez E Selvi E Balistreri E Lorenzini S Maggio R Natale MR Cannabinoids inhibit fibrogenesis in diffuse systemic sclerosis fibroblasts. Rheumatology (2009) 48(9):1050–6. 10.1093/rheumatology/kep189 19589890

[440] Balistreri E Garcia-Gonzalez E Selvi E Akhmetshina A Palumbo K Lorenzini S The cannabinoid WIN55, 212-2 abrogates dermal fibrosis in scleroderma bleomycin model. Ann Rheum Dis (2011) 70(4):695–9. 10.1136/ard.2010.137539 21177293

[441] Bouaboula M Rinaldi M Carayon P Carillon C Delpech B Shire D Cannabinoid-receptor expression in human leukocytes. Eur J Biochem (1993) 214(1):173–80. 10.1111/j.1432-1033.1993.tb17910.x 8508790

[442] Galiegue S Mary S Marchand J Dussossoy D Carriere D Carayon P Expression of central and peripheral cannabinoid receptors in human immune tissues and leukocyte subpopulations. Eur J Biochem (1995) 232(1):54–61. 10.1111/j.1432-1033.1995.tb20780.x 7556170

[443] Kaplan BL. The role of CB1 in immune modulation by cannabinoids. Pharmacol Ther (2013) 137(3):365–74. 10.1016/j.pharmthera.2012.12.004 23261520

[444] Borner C Hollt V Sebald W Kraus J. Transcriptional regulation of the cannabinoid receptor type 1 gene in T cells by cannabinoids. J Leukoc Biol (2007) 81(1):336–43. 10.1189/jlb.0306224 17041005

[445] Do Y McKallip RJ Nagarkatti M Nagarkatti PS. Activation through cannabinoid receptors 1 and 2 on dendritic cells triggers NF-kappaB-dependent apoptosis: novel role for endogenous and exogenous cannabinoids in immunoregulation. J Immunol (2004) 173(4):2373–82. 10.4049/jimmunol.173.4.2373 15294950

[446] Lu T Newton C Perkins I Friedman H Klein TW. Role of cannabinoid receptors in delta-9-tetrahydrocannabinol suppression of IL-12p40 in mouse bone marrow-derived dendritic cells infected with *Legionella pneumophila*. Eur J Pharmacol (2006) 532(1–2):170–7. 10.1016/j.ejphar.2005.12.040 16443217

[447] Springs AE Karmaus PW Crawford RB Kaplan BL Kaminski NE. Effects of targeted deletion of cannabinoid receptors CB1 and CB2 on immune competence and sensitivity to immune modulation by delta9-tetrahydrocannabinol. J Leukoc Biol (2008) 84(6):1574–84. 10.1189/jlb.0508282 18791168 PMC2614598

[448] McKallip RJ Lombard C Martin BR Nagarkatti M Nagarkatti PS. Delta(9)-tetrahydrocannabinol-induced apoptosis in the thymus and spleen as a mechanism of immunosuppression in vitro and in vivo. J Pharmacol Exp Ther (2002) 302(2):451–65. 10.1124/jpet.102.033506 12130702

[449] Basu S Ray A Dittel BN. Cannabinoid receptor 2 is critical for the homing and retention of marginal zone B lineage cells and for efficient T-independent immune responses. J Immunol (2011) 187(11):5720–32. 10.4049/jimmunol.1102195 22048769 PMC3226756

[450] Agudelo M Newton C Widen R Sherwood T Nong L Friedman H Cannabinoid receptor 2 (CB2) mediates immunoglobulin class switching from IgM to IgE in cultures of murine-purified B lymphocytes. J Neuroimmune Pharmacol (2008) 3(1):35–42. 10.1007/s11481-007-9088-9 18247126 PMC2423231

[451] Cencioni MT Chiurchiu V Catanzaro G Borsellino G Bernardi G Battistini L Anandamide suppresses proliferation and cytokine release from primary human T-lymphocytes mainly via CB2 receptors. PLoS One (2010) 5(1):e8688. 10.1371/journal.pone.0008688 20098669 PMC2809084

[452] Rettori E De Laurentiis A Zorrilla Zubilete M Rettori V Elverdin JC. Anti-inflammatory effect of the endocannabinoid anandamide in experimental periodontitis and stress in the rat. Neuroimmunomodulation (2012) 19(5):293–303. 10.1159/000339113 22777139

[453] Suarez-Pinilla P Lopez-Gil J Crespo-Facorro B. Immune system: a possible nexus between cannabinoids and psychosis. Brain Behav Immun (2014) 40:269–82. 10.1016/j.bbi.2014.01.018 24509089

[454] Vannacci A Giannini L Passani MB Di Felice A Pierpaoli S Zagli G The endocannabinoid 2-arachidonylglycerol decreases the immunological activation of Guinea pig mast cells: involvement of nitric oxide and eicosanoids. J Pharmacol Exp Ther (2004) 311(1):256–64. 10.1124/jpet.104.068635 15187170

[455] Kong W Li H Tuma RF Ganea D. Selective CB2 receptor activation ameliorates EAE by reducing Th17 differentiation and immune cell accumulation in the CNS. Cell Immunol (2014) 287(1):1–17. 10.1016/j.cellimm.2013.11.002 24342422 PMC3906668

[456] Mair KM Robinson E Kane KA Pyne S Brett RR Pyne NJ Interaction between anandamide and sphingosine-1-phosphate in mediating vasorelaxation in rat coronary artery. Br J Pharmacol (2010) 161(1):176–92. 10.1111/j.1476-5381.2010.00878.x 20718749 PMC2962826

[457] Adapala RK Thoppil RJ Luther DJ Paruchuri S Meszaros JG Chilian WM TRPV4 channels mediate cardiac fibroblast differentiation by integrating mechanical and soluble signals. J Mol Cell Cardiol (2013) 54:45–52. 10.1016/j.yjmcc.2012.10.016 23142541 PMC3935769

[458] Saghatelian A McKinney MK Bandell M Patapoutian A Cravatt BF. A FAAH-regulated class of N-acyl taurines that activates TRP ion channels. Biochemistry (2006) 45(30):9007–15. 10.1021/bi0608008 16866345

[459] Martinelli A Knapp S Anstee Q Worku M Tommasi A Zucoloto S Effect of a thrombin receptor (protease-activated receptor 1, PAR-1) gene polymorphism in chronic hepatitis C liver fibrosis. J Gastroenterol Hepatol (2008) 23(9):1403–9. 10.1111/j.1440-1746.2007.05220.x 18005014

[460] Materazzi S Pellerito S Di Serio C Paglierani M Naldini A Ardinghi C Analysis of protease-activated receptor-1 and -2 in human scar formation. J Pathol (2007) 212(4):440–9. 10.1002/path.2197 17597495

[461] Eddy AA. Serine proteases, inhibitors and receptors in renal fibrosis. Thromb Haemost (2009) 101(4):656–64. 19350108 PMC3136815

[462] Cevikbas F Seeliger S Fastrich M Hinte H Metze D Kempkes C Role of protease-activated receptors in human skin fibrosis and scleroderma. Exp Dermatol (2011) 20(1):69–71. 10.1111/j.1600-0625.2010.01184.x 21158940

[463] Bogatkevich GS Ludwicka-Bradley A Silver RM. Dabigatran, a direct thrombin inhibitor, demonstrates antifibrotic effects on lung fibroblasts. Arthritis Rheum (2009) 60(11):3455–64. 10.1002/art.24935 19877031 PMC2837365

[464] Raman P Kaplan BL Thompson JT Vanden Heuvel JP Kaminski NE. 15-Deoxy-delta12,14-prostaglandin J2-glycerol ester, a putative metabolite of 2-arachidonyl glycerol, activates peroxisome proliferator activated receptor gamma. Mol Pharmacol (2011) 80(1):201–9. 10.1124/mol.110.070441 21511917 PMC3127542

[465] Rockwell CE Kaminski NE. A cyclooxygenase metabolite of anandamide causes inhibition of interleukin-2 secretion in murine splenocytes. J Pharmacol Exp Ther (2004) 311(2):683–90. 10.1124/jpet.104.065524 15284281

[466] Mutlu GM Budinger GR Wu M Lam AP Zirk A Rivera S Proteasomal inhibition after injury prevents fibrosis by modulating TGF-beta(1) signalling. Thorax (2012) 67(2):139–46. 10.1136/thoraxjnl-2011-200717 21921091 PMC3595535

[467] Servettaz A Kavian N Nicco C Deveaux V Chereau C Wang A Targeting the cannabinoid pathway limits the development of fibrosis and autoimmunity in a mouse model of systemic sclerosis. Am J Pathol (2010) 177(1):187–96. 10.2353/ajpath.2010.090763 20508030 PMC2893662

[468] Marquart S Zerr P Akhmetshina A Palumbo K Reich N Tomcik M Inactivation of the cannabinoid receptor CB1 prevents leukocyte infiltration and experimental fibrosis. Arthritis Rheum (2010) 62(11):3467–76. 10.1002/art.27642 20617520

